# Aromatic oligoesters as novel helix mimetic scaffolds

**DOI:** 10.1016/j.bmc.2023.117311

**Published:** 2023-05-03

**Authors:** Muhammed Haque, Theo Flack, Ravi Singh, Archie Wall, Guilherme Vieira de Castro, Lishen Jiang, Andrew J.P. White, Anna Barnard

**Affiliations:** Department of Chemistry, Molecular Sciences Research Hub, Imperial College London, London W12 0BZ, UK

**Keywords:** Helix mimetic, Protein-protein interactions, Aromatic oligoester

## Abstract

The design, synthesis, and conformational analysis of a novel aromatic oligoester helix mimetic scaffold is reported. A range of amino acid-type side-chain functionality can be readily incorporated into monomer building blocks over three facile synthetic steps. Analysis of representative dimers revealed a stable conformer capable of effective mimicry of a canonical α-helix and the scaffold was found to be surprisingly stable to degradation in aqueous solutions at acidic and neutral pH.

## Graphical abstract

**Figure:**
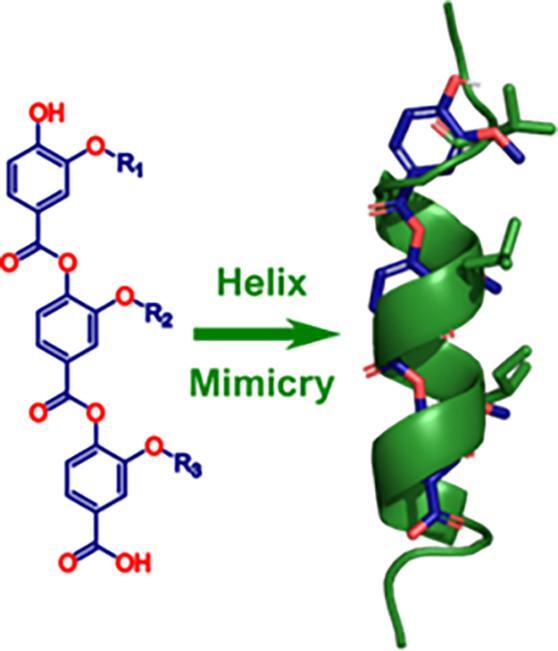

## 1 Introduction

**Fig. 1:**
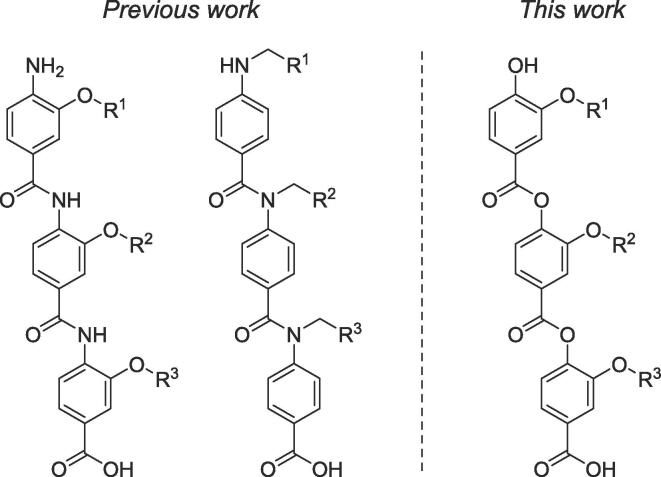
Previously published 3-O-alkylated^21^ and N-alkylated^22^ aromatic oligoamide α-helix mimetics and our novel aromatic oligoester scaffold.

The interactions between protein binding partners critically mediate almost all biological processes, with the size of the human protein–protein interaction (PPI) interactome thought to be ∼ 650,000 pair-wise interactions.^1^ As such, considerable effort has been directed towards PPI modulation and inhibition over recent decades with α-helix mediated interactions garnering the most attention.^2^ Multiple strategies for targeting helix-mediated PPIs have emerged including small molecule screening,^3^ peptidic^4^, ^5^ and peptidomimetic (or proteomimetic) approaches.^6^, ^7^, ^8^ The latter involves the development of molecules able to replicate the spacial projection of the binding amino acid side chains on an α-helix from a central scaffold. Many such helix-mimetic scaffolds have been developed since the initial terphenyls^9^, ^10^, ^11^, ^12^ to now encompass oxopiperazines,^13^ oligoureas^14^ and triazine-piperazine-triazine scaffolds,^15^ among others.^16^, ^17^, ^18^ However, it is aromatic oligoamides^19^, ^8^ which have dominated this space owing to their synthetic tractability and amenability to automated solid-phase synthesis^20^, ^21^, ^22^, ^23^ with examples showing promising competitive inhibition of disease relevant PPIs.^24^, ^25^, ^26^, ^27^, ^28^, ^29^, ^30^, ^31^, ^32^ Despite their dominance, aromatic oligoamide scaffolds are not without drawbacks; the poor nucleophilicity of anilines in combination with weakly electrophilic benzoic acids necessitates harsh coupling conditions^23^ and substituted anilines do not always adopt a mimetic conformation without internal hydrogen bonding interactions which reduce aqueous solubility.^33^, ^34^ We reasoned that perhaps some of these issues could be circumvented by replacing the central amide bond with an ester linkage (Fig. 1). This would enable coupling between monomers with more reactive phenol nucleophiles (vs aniline) and would take advantage of the conformational preference of esters for an extended, mimetic *s-cis* orientation. Herein, we describe the design, synthesis, and analysis of a novel 3-*O*-alkylated aromatic oligoester helix mimetic scaffold capable of effective mimicry of a canonical α-helix and complete stability to degradation at biologically relevant pH.

## 2 Results and discussion

### 2.1 Monomer synthesis

**Scheme 1:**
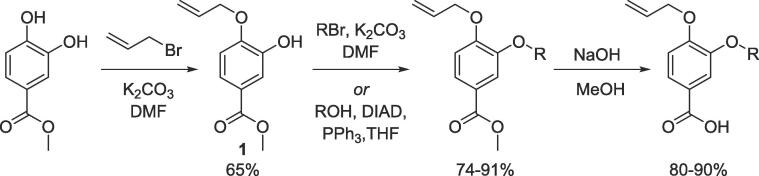
Synthesis of Allyl-protected monomers from 3,4-dihydroxybenzoate.

Our oligoester scaffold was designed to minimise synthetic steps, avoid complex chemistry and enable efficient generation of a range of different ‘side chain’ functionalities. Taking inspiration from established routes to oligoamide scaffolds,^21^ we chose to start from methyl 3,4-dihydroxybenzoate and install a protecting group *para* to the methyl ester and subsequently introduce side-chains at the *meta*-hydroxy to afford a library of protected monomer building blocks (Scheme 1). We chose to incorporate a *para*-allyl protecting group based on its low steric hindrance, minimal electronic effects and, primarily, acid and base stability allowing for orthogonal deprotection of the methyl ester and any acid-labile side chain protecting groups. Importantly, this leaves open the possibility of expansion of our methodology in future to automated solid-phase synthesis allowing for future global side chain deprotection and resin cleavage. To install the allyl protecting group, two sets of conditions were trialled: substitution with allyl bromide and Mitsunobu conditions with the former offering superior yields and more straightforward purification. Despite the electron-withdrawing effect of the ester group, at larger scales di-allyl protection was observed. Therefore, conditions were optimised through adjustment of the quantity of base to minimise this but the undesired bis(allyloxy) by-product was still formed necessitating purification. Nevertheless, the reaction could still be performed on a large (50 mmol) scale affording gram quantities of a common starting material for all monomer building blocks. This provides an advantage over oligoamides in which the Fmoc protecting group at the equivalent position is installed after the side chain is introduced.^21^ With the singly protected monomer obtained, NOESY NMR was used to confirm isolation of the *para*-allyl protected compound **1** (Fig. S1).

Side chain functionality could then be introduced at the *meta* position using a range of bromides or alcohols employing substitution or Mitsunobu chemistry, respectively. Fourteen of the twenty natural amino acid side chains were initially considered: glycine and alanine were discounted as they do not form typically form meaningful interactions, glutamic acid and glutamine were considered represented with aspartic acid and asparagine mimics respectively, cysteine derivatives could prove problematic given their disulphide bond forming ability and proline is not commonly found within helical protein regions, acting instead as a helix breaker. Unfortunately, problems were encountered when attempting to introduce mimics of both tyrosine and arginine. Both *tert*butyl (4-hydroxylmethyl)phenyl) carbonate and di-Boc protected 1-(3-hydroxypropyl)guanidine were unreactive under Mitsunobu conditions and generation of the equivalent bromide derivatives also proved unsuccessful. However, using our optimised conditions, it was possible to generate a library of monomers mimicking twelve of the proteogenic amino acids (Table 1).

**Table 1:** 3-*O*-alkylated ester monomers synthesised.

**R**	**Ester compound no. (yield)**	**Acid compound no. (yield)**
	**2** (91%)	**3** (84%)
	**4** (85%)	**5** (88%)
	**6** (84%)	**7** (80%)
	**8** (77%)	**9** (82%)
	**10** (85%)	**11** (90%)
	**12** (74%)	**13** (90%) *tBu ester deprotected
	**14** (83%)	**15** (90%)
	**16** (80%)	**17** (88%)
	**18** (84%)	**19** (88%)
	**20** (78%)	**21** (84%)
	**22** (74%)	N/A
	**23** (85%)	N/A

The final step in the synthesis of the aromatic oligoester monomers was ester hydrolysis which was readily achieved for most cases using either strong (NaOH) or weak (LiOH) base selected based on side-chain sensitivity. However, in the case of aspartic acid mimic **12** this resulted in side chain deprotection and for tryptophan mimic **22** side chain cleavage, likely via Boc deprotection in a similar manner to that previously reported for oligoamide helix mimetics.^22^ For threonine derivative **23** no reaction occurred.

### 2.2 Dimer synthesis

**Scheme 2:**
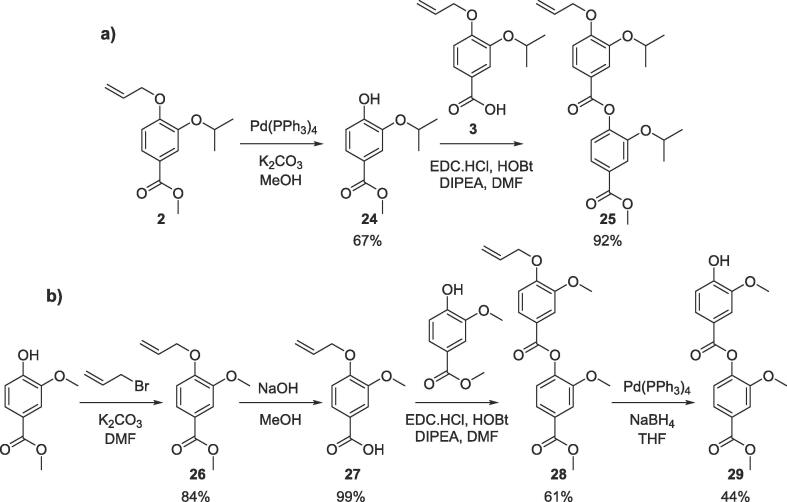
Synthesis of representative dimers **25**, **28** and **29**.

With a library of monomer building blocks in hand, we sought to develop conditions for the synthesis of a representative dimer which could be readily applied for the generation of longer oligomers both in solution and on solid phase. Initially a dimer was made based on monomers furnished with isopropyl side chains. First, the allyl group on monomer **2** was removed using Pd(PPh_3_)_4_ and K_2_CO_3_ affording phenol **24** which could then be coupled to carboxylate **3** using ester coupling conditions (EDC, HOBt and DIPEA) to afford the target dimer (**25**) in excellent yields (Scheme 2). To facilitate conformational analysis, a second dimer based on methyl side chains was also generated starting from commercially available methyl 4-hydroxy-3-methoxybenzoate which was subjected to *para*-allyl protection and subsequent methyl ester deprotection before being coupled using the same conditions established for dimer **25** to afford methyl functionalised dimer **28**. Interestingly, when we attempted to remove the allyl protecting group from this dimer (**28**) using the conditions established for monomer **24,** a transesterification with the methanol solvent occurred. We therefore switched to using the same palladium catalyst (Pd(PPh_3_)_4_) but in combination with NaBH_4_ as a reducing agent to synthesise dimer **29.**

### 2.3 Conformational analysis

To study the conformational preferences of the oligoester scaffold dimers **25** and **28** were subjected to a combination of solution and solid-state investigations since both contain all the structural features common to any extended oligomers: (i) a central ester bond and (ii) the potential for *syn*/*anti* conformers with regards to the *O*-alkyl side chains. Clearly, an extended ester and side chain *syn* conformation is optimal for effective mimicry of the binding groups along a single face of an α-helix.

**Fig. 2:**
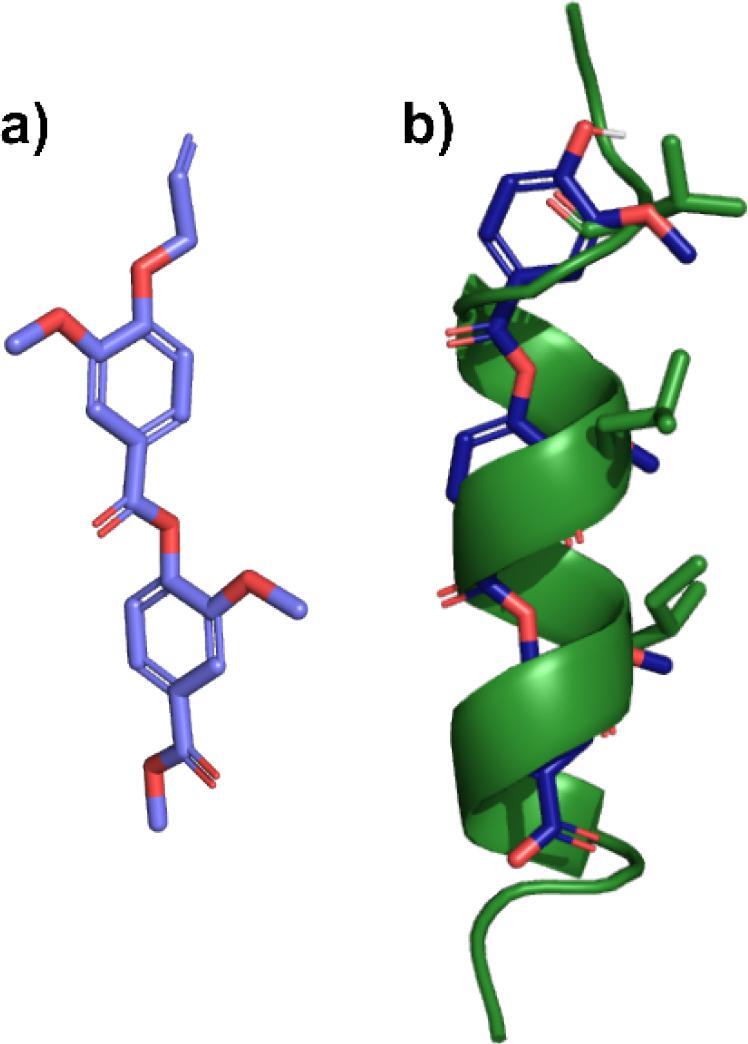
A) solid state structure of dimer **28** and b) overlay of a computationally generated trimer with the Bak peptide helix showing good side chain alignment (0.83 Å).

Single crystals suitable for X-ray diffraction were obtained for dimer **28** from slow evaporation of ethyl acetate (Figure 2a). A single conformer was observed in the solid state with, pleasingly, the *s-cis*-isomer about the central ester bond. This is to be expected due to the stabilising influence of an anomeric-type effect (*n*O_sp2_ → σ*_C_—_O_), reduction in steric strain and the smaller dipole moment of the *s-cis*-isomer. Single crystals of **25** could not be obtained so a variable temperature (VT) NMR experiment was carried out to determine whether multiple conformations of the scaffold could exist in solution. Over a temperature range of 218–328 K in CDCl_3_ only a single conformer was observed for this dimer (Figure S2). Based on the solid-state structure of **28** and the known conformational preferences of esters we concluded that our oligoester scaffold exists in a single stable extended conformation likely capable of effective helix mimicry. The solid-state structure also showed the side chains of **28** in an *anti-*conformation which would require rotation about the central aryl-C(O) bond to form a ‘mimetic’ arrangement of binding groups. We therefore calculated the barrier to rotation about this bond and confirmed that rapid bond rotation at room temperature is feasible (6.22 kcal/mol). This is also confirmed by our observation of a single conformer by VT NMR suggesting that this bond rotation is fast on the NMR timescale at all temperatures studied.

To confirm the ability of our scaffold to present side chains at similar orientations to those on a canonical α-helix we overlayed a computationally generated trimer structure with the i*, i + 4* and *i +* 7 residues of the published crystal structure of the Bak peptide helix^35^ and obtained an RMSD of 0.83 Å demonstrating good agreement (Figure 2b). These values are also similar to those obtained for oligoamide helix mimetics (∼0.49 Å)^27^, ^36^ suggesting that similar PPI inhibition behaviour would be possible for our oligoester analogues.

### 2.4 Aqueous stability

**Fig. 3:**
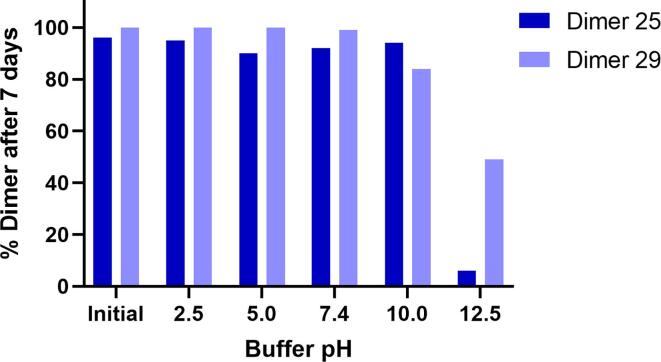
Dimers **25** and **29** were incubated for 7 days in a range of buffered pH solutions and % dimer remaining measured by integration of UV peaks from an HPLC Diode array trace.

A vital property any biologically active molecule must possess is aqueous stability. Human serum pH is tightly regulated around 7.4, with intracellular pH controlled between pH 6.0–7.2, and gastric pH between 1.4–3.5.^37^ Thus, for oligoesters to serve as effective PPI inhibitors they must demonstrate sufficient stability at such pH ranges, to be capable of reaching the target without suffering from degradation that ultimately eliminates any activity. Given the prevalence of ester linkages in pro-drug structures^38^ and the facile hydrolysis of esters in aqueous media, the stability of our scaffold was a concern. We therefore investigated the stability of dimers **25** and **29** over extended periods of time in aqueous solution from pH2.5–12.5. To monitor dimer stability over time, 10 mM pH buffer solutions of: 2.5(acetate), 5.0(acetate), 7.4 (phosphate-buffered saline), 10.0 (bicarbonate), and 12.5 (bicarbonate) were selected, to obtain a pH range similar to those present within the human body. Since both dimers are insoluble in water, they were firstly dissolved in acetonitrile, before addition to the buffered solutions to form a 5 mg/mL dimer solution (50% MeCN: buffer). Degradation was monitored via TLC over time, and the final solution composition was determined by LCMS after 7 days (Fig 3 *and* Figure S3).

Promisingly, we detected no significant degradation of either dimer in the pH range 2.5 –10.0, even after 7 days, suggesting the ester bond has strong pH stability with dimer % remaining consistently over 90% (i.e., less than 10% ester hydrolysis) when determined from the corresponding HPLC UV traces. Strong stability at pH 7.4 is particularly important, indicating the ester bond would be capable of travelling within human serum without suffering from degradation. Additionally, pH 2.5 stability means the ester bond should be capable of surviving the acidic conditions of the stomach without succumbing to hydrolysis. Such results should also be applicable to trimers or longer oligoesters since the core nature of the ester bond would remain un-changed even in extended chains. However, dimer degradation, via ester bond cleavage, does occur at pH 12.5 after 7 days, with dimer % overall decreasing to 6% for **25** and 49% for **29**. Some drift to lower pH from 12.5 was observed for **29**, suspected to be the result of deprotonation of the phenol, which accounts for the higher stability observed for this dimer. Further analysis of dimer **25** (Figure S3) confirmed that the one original UV-active peak corresponding to starting dimer, degraded to two monomer peaks with *m*/*z* appropriate for the phenol and carboxylic acid by-products resulting from ester hydrolysis. Nonetheless, this hydrolysis occurs only after 5 days at pH 12.5, an acceptable duration of ester bond stability particularly at this extreme basicity, beyond the range any inhibitor would encounter in a biological environment.

## 3 Conclusions

We have described the design, synthesis, and analysis of a novel aromatic oligoester scaffold capable of mimicry of the side chains *i*, *i + 3/4* and *i + 7* of a canonical α-helix. We have developed a facile synthetic route to access a range of monomer building blocks covering a variety of side chain functionalisation similar to the diversity of those found in natural amino acids and, importantly, our methodology is readily expandable to encompass the vast chemical space not sampled in native proteins. Conformational analysis demonstrated a favourable extended, mimetic conformation for the ester scaffold and good agreement between the spacial projection of side chains on a model α-helix and those projected from our scaffold backbone. The ester linkage was found to be surprisingly stable over a range of pH values (2.5–10) making the application of this class of helix mimetic in a biologically relevant environment highly feasible. With further development of higher-throughput automated solid-phase synthetic methods, oligoester scaffolds could potentially offer a viable alternative to current oligoamide analogues overcoming some of the issues with their synthetic accessibility and non-mimetic conformational preferences.

## 4 Experimental

### 4.1 General considerations

All reagents were obtained from commercial sources unless otherwise stated. All solvents used were anhydrous. All water used was of distilled (dH_2_O) or Milli-Q quality. Solvents were removed using Büchi rotary evaporators. Reactions were conducted under inert (nitrogen) atmosphere. Column chromatography purification was performed using silica gel (40–63 μM, Geduran) with the solvent ratios specified. Reaction monitoring was done using Thin Layer Chromatography (TLC) on silica Merck 60 F254 aluminium plates, using Ultraviolet (UV) 254/365 nm for visualization. ^1^H, ^13^C, COSY, and NOESY spectra were all measured on a Bruker DRX400, with chemical shifts reported in parts per million (ppm) downfield from trimethylsilane (TMS) as the internal reference, coupling constants are reported in hertz (Hz). Variable temperature NMR was conducted between 218 K and 328 K on a Bruker DRX500. Multiplicity is reported as singlet (s), doublet (d), triplet (t), quartet (q), multiplet (m), or combinations of these. Infrared spectra were obtained using a Perkin-Elmer Spectrum 100 FTIR spectrometer, reported in cm^−1^, and obtained in solid-state. High-resolution mass spectrometry (HRMS) of products were obtained with an in-house high-resolution mass spectrometer (Waters LCT Premier Electrospray Time of Flight spectrometer), using an electron spray ionization (ESI) technique. Melting points were obtained using a Büchi 510 melting point machine, with a gradient of 1 °C per minute. X-ray crystallography was performed by the Imperial College X-ray Crystallography Service. Single crystal X-ray data were collected using an Agilent Xcalibur 3 E diffractometer with Mo-K(alpha) radiation at 173 K.

### 4.2 Monomer and dimer synthesis

#### 4.2.1 Compound 1 – Methyl 4-(allyloxy)-3-hydroxybenzoate

**Figure:**
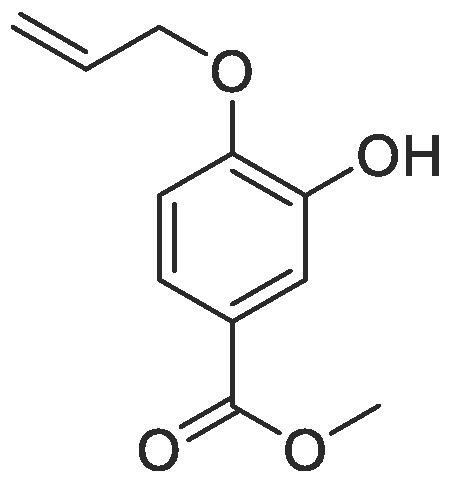

Methyl 3,4-dihydroxybenzoate (8.41 g, 50.0 mmol), allyl bromide (4.32 mL, 50.0 mmol), and K_2_CO_3_ (8.29 g, 60.0 mmol) were added to anhydrous DMF (50 mL) and stirred at r.t. for 6 h. The resulting yellow–brown precipitate was extracted using ethyl acetate (2 × 200 mL) and the combined organic layers washed with H_2_O (3 × 100 mL) and brine (3 × 100 mL), dried over MgSO_4_, filtered and concentrated *in vacuo*. The crude mixture was purified by column chromatography (1:5 EtOAc:*n*-Pentane) affording the title compound as a white crystalline solid. (6.78 g, 32.5 mmol, 65%); *R_f_* = 0.33 (1:5 EtOAc:*n*-Pentane); mp = 67–68 °C; ^1^H NMR (400 MHz, CDCl_3_) δ_H_: 7.60–7.59 (dd, Ar***H***, *J* = 4.0, 2.0, 1H), 7.57 (d, Ar***H***, *J* = 2.0, 1H), 6.86 (d, Ar***H***, *J* = 8.0, 1H), 6.12–6.00 (m, C***H*** = CH_2_, 1H), 5.41 (dq, C***H_2_*** = CH, *J* = 17, 1.5, 1H), 5.34 (dq, C***H_2_*** = CH, *J* = 10.5, 1.2, 1H), 4.65 (dt, C***H_2_***OAr, *J* = 8.0, 4.0, 2H), 3.88 (s, COOC***H_3_***, 3H); ^13^C NMR (125 MHz, CDCl_3_) δ_C_: 166.9 (***C***=O), 149.7 (***C***OCH_2_), 145.5 (***C***OH), 132.2 (***C***H = CH_2_), 123.2 (***C***COOCH_3_), 122.5 (Ar***C***), 118.5 (***C***H_2_ = CH), 116.0 (Ar***C***), 111.3 (Ar***C***), 69.6 (***C***H_2_OAr), 51.9 (***C***H_3_COO); *ν*_max_/cm^−1^ (solid state) = 3370 (OH, br), 1703 (CO, s), 1508 (C=C), 1214 (C—O); ESI-HRMS found *m*/*z* 207.0657 [M−H]^-^, C_11_H_11_O_4_ requires 207.0663.

#### 4.2.2 Compound 2 – Methyl 4-(allyloxy)-3-isopropoxybenzoate

**Figure:**
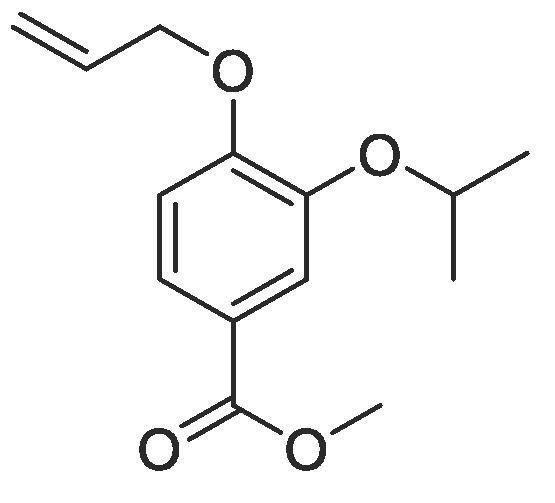

Methyl 4-(allyloxy)-3-hydroxybenzoate (1.04 g, 5.00 mmol), 2-bromopropane (2.35 mL, 25.0 mmol), and K_2_CO_3_ (5.53 g, 40.0 mmol) were added to anhydrous DMF (25 mL) and stirred at r.t. for 6 h. The reaction mixture was diluted with ethyl acetate (100 mL) and the combined organic layers washed with H_2_O (3 × 100 mL) and brine (3 × 100 mL), dried over MgSO_4_, filtered and concentrated *in vacuo* affording the title compound as a yellow oil (1.14 g, 45.5 mmol, 91%); *R_f_* = 0.60 (1:5 EtOAc:*n*-Pentane), ^1^H NMR (400 MHz, CDCl_3_) δ_H_: 7.65 (dd, Ar***H***, *J* = 12, 4, 1H), 7.60 (d, Ar***H***, *J* = 4, 1H), 6.90 (d, Ar***H***, *J* = 8, 1H), 6.11–6.05 (m, C***H*** = CH_2_, 1H), 5.44 (dq, C***H_2_*** = CH, *J* = 16, 1.6, 1H), 5.31 (dq, C***H_2_*** = CH, *J* = 8, 1.6, 1H), 4.66 (dt, C***H_2_***OAr, *J* = 8, 4, 2H), 4.59 (quint, C***H***(CH_3_)_2_, *J* = 6, 1H), 3.90 (s, COOC***H_3_***, 3H), 1.39 (d, CH(***H_3_***)_2_, *J* = 6, 6H); ^13^C NMR (125 MHz, CDCl_3_) δ_C_: 166.9 (***C***=O), 153.7 (***C***OCH_2_), 147.3 (***C***OCH), 132.9 (***C***H = CH_2_), 123.8 (***C***COOCH_3_), 122.9 (Ar***C***), 117.7 (***C***H_2_ = CH_2_), 117.6 (Ar***C***), 112.9 (Ar***C***), 72.0 (***C***H(CH_3_)_2_), 69.6 (***C***H_2_OAr), 51.9 (***C***H_3_COO), 22.1 ((***C***H_3_)_2_CH); *ν*_max_/cm^−1^ (solid state) = 1737 (CO, s), 1372 (C=C), 1236 (C—O); ESI-HRMS found *m*/*z* 251.1289 [M + H]^+^, C_14_H_19_O_4_ requires 251.1283.

#### 4.2.3 Compound 3 – 4-(allyloxy)-3-isopropoxybenzoic acid

**Figure:**
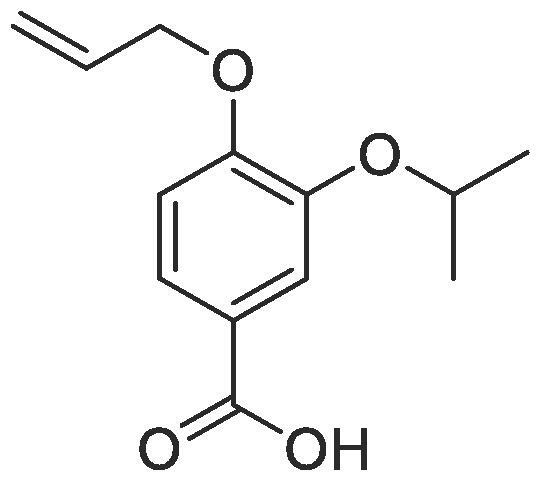

Methyl 4-(allyloxy)-3-isopropoxybenzoate (0.570 g, 2.28 mmol) was added to 2 M NaOH (3.42 mL, 6.83 mmol) and MeOH (3.42 mL) and the reaction mixture stirred at 50 °C for 1 h. The reaction was acidified to pH 2–3 by drop-wise addition of 2 M HCl. The product was extracted with ethyl acetate (2 × 50 mL) and the combined organic layers were dried over MgSO_4_, filtered and concentrated *in vacuo* affording the title compound as a light-yellow solid (0.453 g, 1.92 mmol, 84%); *Rf* = 0.00 (1:5 EtOAc:*n*-Pentane); mp = 108–110 °C; ^1^H NMR (400 MHz, CDCl_3_) δ_H_: 8.91 (br s, O***H***, 1H), 7.72 (d, Ar***H***, *J* = 8, 1H), 7.65 (d, Ar***H***, *J =* 4, 1H), 6.90 (d, Ar***H***, *J* = 8, 1H), 6.12–6.05 (m, C***H*** = CH_2_, 1H), 5.38 (d, C***H_2_*** = CH, *J* = 18, 1H), 5.32 (d, C***H_2_*** = CH, *J* = 8, 1H), 4.67 (d, C***H_2_***OAr, *J* = 8, 2H), 4.59 (quint, C***H***(CH_3_)_2_, *J* = 8, 1H), 1.39 (d, CH(C***H_3_***)_2_, *J* = 8, 6H); ^13^C NMR (125 MHz, CDCl_3_) δ_C_: 163.2 (***C***=O), 153.6 (***C***OCH_2_), 141.3 (***C***OCH), 130.9 (***C***H = CH_2_), 129.5 (***C***COOH), 128.6 (Ar***C***), 123.5 (***C***H_2_ = CH_2_), 120.1 (Ar***C***), 116.9 (Ar***C***), 68.5(***C***H_2_OAr), 67.0 (***C***H(CH_3_)_2_), 26.1 ((***C***H_3_)_2_CH); *ν*_max_/cm^−1^ (solid state) = 2929 (OH, br), 1671 (CO, s), 1441 (C=C), 1263 (C—O); ESI-HRMS found *m*/*z* 235.0975 [M−H]^-^, C_13_H_16_O_4_ requires 235.0970.

#### 4.2.4 Compound 4 – Methyl 4-(allyloxy)-3-isobutoxybenzoate

**Figure:**
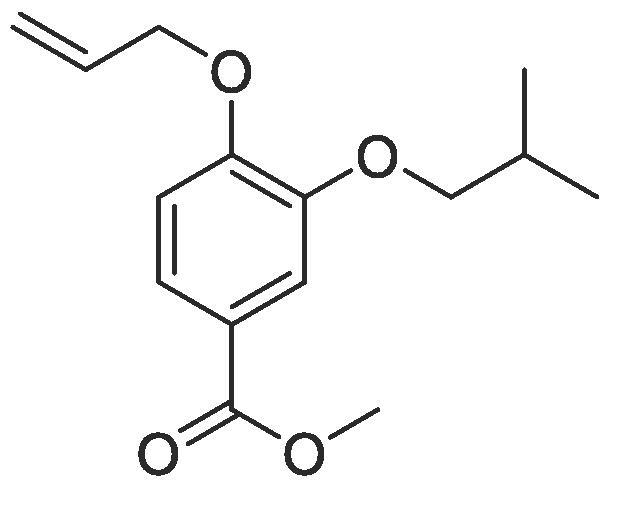

Methyl 4-(allyloxy)-3-hydroxybenzoate (0.104 g, 0.500 mmol), 1-bromo-2-methylpropane (0.272 mL, 2.50 mmol), and K_2_CO_3_ (0.553 g, 0.400 mmol) were added to anhydrous DMF (2.5 mL) and stirred at r.t. for 6 h. The reaction mixture was diluted with ethyl acetate (100 mL) and the combined organic layers washed with H_2_O (3 × 100 mL) and brine (3 × 100 mL), dried over MgSO_4_, filtered and concentrated *in vacuo* affording the title compound as a yellow oil (0.112 g, 0.425 mmol, 85%); *R_f_* = 0.68 (1:5 EtOAc:*n*-Pentane); ^1^H NMR (400 MHz, CDCl_3_) δ_H_: 7.64 (dd, Ar***H***, *J* = 12, 4, 1H), 7.56 (d, Ar***H***, *J* = 4, 1H), 6.89 (d, Ar***H***, *J* = 8, 1H), 6.11–6.04 (m, C***H*** = CH_2_, 1H), 5.46 (dq, C***H_2_*** = CH, *J* = 16, 4, 1H), 5.32 (dq, C***H_2_*** = CH, *J* = 8, 4, 1H), 4.65 (dt, C***H_2_***OAr, *J* = 8, 4, 2H), 3.90 (s, COOC***H_3_***, 3H), 3.83 (d, C***H***_2_CH, *J* = 8, 2H), 2.18 (sept, CH_2_C***H***, *J* = 8, 1H), 1.07 (d, CH(C***H_3_***)_2_, *J* = 8, 6H); ^13^C NMR (125 MHz, CDCl_3_) δ_C_: 167.0 (***C***=O), 152.6 (***C***OCH_2_), 148.9 (***C***OCH_2_CH), 132.9 (***C***H = CH_2_), 123.4 (***C***COOCH_3_), 123.0 (Ar***C***), 117.6 (***C***H_2_ = CH), 114.2 (Ar***C***), 112.8 (Ar***C***), 75.6 (***C***H_2_CH), 69.6 (***C***H_2_OAr), 52.0 (***C***H_3_COO), 27.7 (***C***H(CH_3_)_2_), 19.8 ((***C***H_3_)_2_CH); *ν*_max_/cm^−1^ (solid state) = 1714 (CO, s), 1510 (C=C), 1267 (C—O); ESI-HRMS found *m*/*z* 265.1447 [M + H]^+^, C_15_H_21_O_4_ requires 265.1440.

#### 4.2.5 Compound 5–4-(allyloxy)-3-isobutoxybenzoic acid

**Figure:**
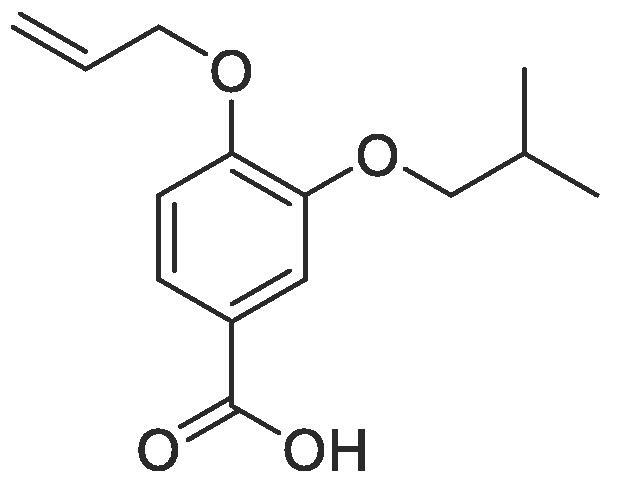

Methyl 4-(allyloxy)-3-isobutoxybenzoate (0.112 g, 0.425 mmol) was added to 2 M NaOH (0.638 mL, 1.28 mmol) and MeOH (0.638 mL) and the reaction mixture stirred at 50 °C for 1 h. The reaction was acidified to pH 2–3 by drop-wise addition of 2 M HCl. The product was extracted with ethyl acetate (2 × 50 mL) and the combined organic layers were dried over MgSO_4_, filtered and concentrated *in vacuo* affording the title compound as a light-yellow solid (0.094 g, 0.374 mmol, 88%); *R_f_* = 0.00 (1:5 EtOAc:*n*-Pentane); mp = 115–116 °C; ^1^H NMR (400 MHz, DMSO‑*d*_6_) δ_H_: 7.52 (dd, Ar***H***, *J* = 12, 8, 1H), 7.43 (d, Ar***H***, *J* = 1, 1H), 7.04 (d, Ar***H***, *J* = 8, 1H), 6.08–5.99 (m, C***H*** = CH_2_, 1H), 5.42 (dq, C***H_2_*** = CH, *J* = 16, 4, 1H), 5.26 (dq, C***H_2_*** = CH, *J* = 8, 4, 1H), 4.63 (dt, C**H_2_**OAr, *J* = 8, 4, 2H), 3.76 (d, C***H_2_***CH, *J* = 4, 2H), 2.03 (quint, C***H***(CH_3_)_2_, *J* = 8, 1H), 0.98 (d, CH(C***H_3_***)_2_, *J* = 8, 6H); ^13^C NMR (125 MHz, CDCl_3_) δ_C_: 167.6 (***C***=O), 152.2 (***C***OCH_2_), 148.5 (***C***OCH_2_CH), 133.8 (***C***H = CH_2_), 123.7 (***C***COOH), 123.6 (Ar***C***), 117.7 (***C***H_2_ = CH), 114.1 (Ar***C***), 113.3 (Ar***C***), 75.1 (***C***H_2_CH), 69.1 (***C***H_2_OAr), 28.3 (***C***H(CH_3_)_2_), 19.5 ((***C***H_3_)_2_CH); *ν*_max_/cm^−1^ (solid state) = 2927 (OH, br), 1675 (CO, s), 1436 (C=C), 1271 (C—O); ESI-HRMS found *m*/*z* 249.1131 [M−H]^-^, C_14_H_17_O_4_ requires 249.1127.

#### 4.2.6 Compound 6 – Methyl (*R*)-4-(allyloxy)-3-(2-methylbutoxy)benzoate

**Figure:**
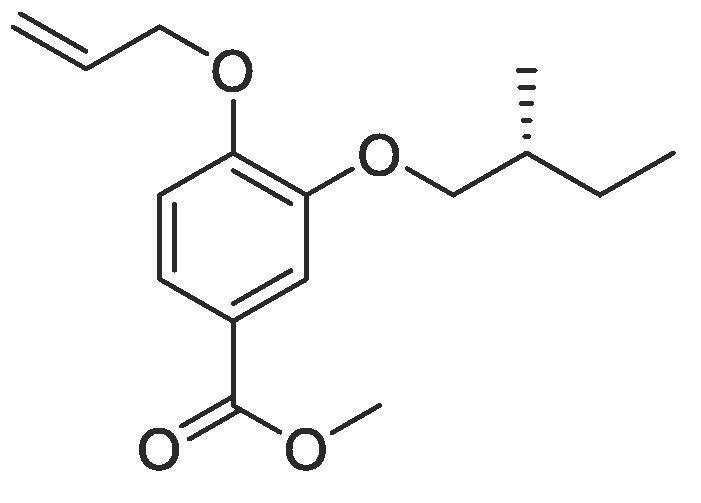

Methyl 4-(allyloxy)-3-hydroxybenzoate (0.104 g, 0.500 mmol), (*R*)-1-bromo-2-methylbutane (0.272 mL, 2.50 mmol), and K_2_CO_3_ (0.553 g, 0.400 mmol) were added to anhydrous DMF (2.5 mL) and stirred at r.t. for 6 h. The reaction mixture was diluted with ethyl acetate (50 mL) and the combined organic layers washed with H_2_O (3 × 50 mL) and brine (3 × 50 mL), dried over MgSO_4_, filtered and concentrated *in vacuo* affording the title compound as a yellow oil (0.111 g, 0.420 mmol, 84%); *Rf* = 0.66 (1:5 EtOAc:*n*-Pentane); ^1^H NMR (400 MHz, CDCl_3_) δ_H_: 7.61 (dd, Ar***H***, *J* = 8, 4, 1H), 7.54 (Ar***H***, J = 4, 1H), 6.87 (d, Ar***H***, *J* = 8, 1H), 6.09–6.01 (m, C***H*** = CH_2_, 1H), 5.46 (dq, C***H_2_*** = CH, *J* = 16, 4, 1H), 5.30 (dq, C***H_2_*** = CH, *J* = 8, 4, 1H), 4.63 (dt, C***H_2_***OAr, *J* = 8, 4, 2H), 3.93–3.79 (m, C***H_2_***CH(CH_3_), 2H), 3.88 (s, COOC***H_3_***, 3H), 1.95 (sex, C***H***C_2_H_5_, *J* = 8, 1H), 1.63–1.56 (m, C***H_2_***CH_3_, 1H), 1.32–1.25 (m, C***H_2_***CH_3_, 1H), 1.04 (d, C***H_3_***CH, *J* = 8, 3H), 0.96 (t, C***H_3_***CH_2_, *J* = 8, 3H); ^13^C NMR (125 MHz, CDCl_3_) δ_C_: 167.0 (***C***=O), 152.5 (***C***OCH_2_), 148.9 (***C***OCH), 132.8 (***C***H = CH_2_), 123.3 (***C***COOCH_3_), 122.9 (Ar***C***), 117.5 (***C***H_2_ = CH), 114.0 (Ar***C***), 112.6 (Ar***C***), 73.8 (***C***HCH_3_), 69.5 (***C***H_2_OAr), 52.0 (***C***H_3_COO), 34.7 (***C***H_2_CH(CH_3_)) 26.2 (***C***H_2_CH) 16.6 (***C***H_3_CH), 11.4 (***C***H_3_CH_2_); *ν*_max_/cm^−1^ (solid state) = 1715 (CO, s), 1511 (C=C), 1266 (C—O); ESI-HRMS found *m*/*z* 279.1608 [M + H]^+^, C_16_H_23_O_4_ requires 279.1596.

#### 4.2.7 Compound 7 – (*R*)-4-(allyloxy)-3-(2-methylbutoxy)benzoic acid

**Figure:**
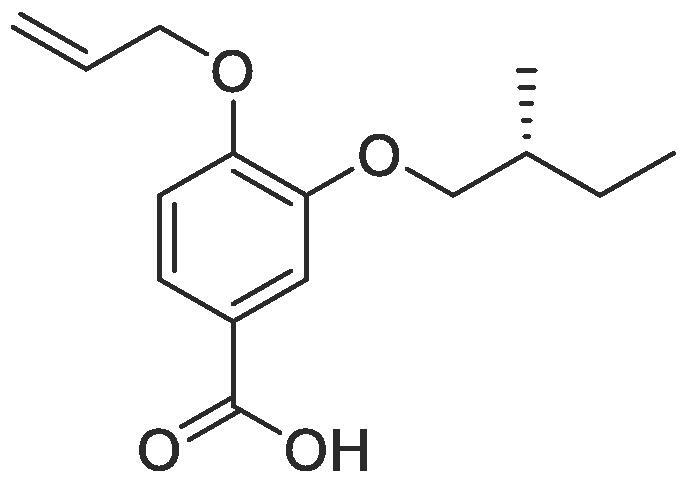

Methyl (*R*)-4-(allyloxy)-3-(2-methylbutoxy)benzoate (0.111 g, 0.420 mmol) was added to 2 M NaOH (0.630 mL, 1.26 mmol) and MeOH (0.630 mL) and the reaction mixture stirred at 50 °C for 1 h. The reaction was acidified to pH 2–3 by drop-wise addition of 2 M HCl. The product was extracted with ethyl acetate (2 × 50 mL) and the combined organic layers were dried over MgSO_4_, filtered and concentrated *in vacuo* affording the title compound as a light-yellow solid (0.084 g, 0.336 mmol, 80%); *R_f_* = 0.00 (1:5 EtOAc:*n*-Pentane); mp = 116–117 °C; ^1^H NMR (400 MHz, DMSO‑*d*_6_) δ_H_: 7.46 (d, Ar***H***, *J* = 4, 1H), 7.43 (dd, Ar***H***, *J* = 8, 4, 1H), 6.86 (d, Ar***H***, *J* = 8, 1H), 6.06–5.99 (m, C***H*** = CH_2_, 1H), 5.40 (dq, C***H_2_*** = CH, *J* = 16, 4, 1H), 5.22 (dq, C***H_2_*** = CH, *J* = 8, 4, 1H), 4.54 (dt, C***H_2_***OAr, *J* = 8, 4, 2H), 3.83–3.71 (m, C***H_2_***CH(CH_3_), 2H), 1.82–1.77 (m, C***H***C_2_H_5_, 1H), 1.55–1.49 (m, C***H_2_***CH_3_, 1H), 1.24–1.21 (m, C***H_2_***CH_3_, 1H), 0.97 (d, C***H_3_***CH, *J* = 8, 3H), 0.90 (t, C***H_3_***CH_2_, *J* = 8, 3H); Insufficient material limited ability to record ^13^C NMR spectra. *ν*_max_/cm^−1^ (solid state) = 2921 (OH, br), 1678 (CO, s), 1436 (C=C), 1272 (C—O).

#### 4.2.8 Compound 8 – Methyl 4-(allyloxy)-3-(benzyloxy)benzoate

**Figure:**
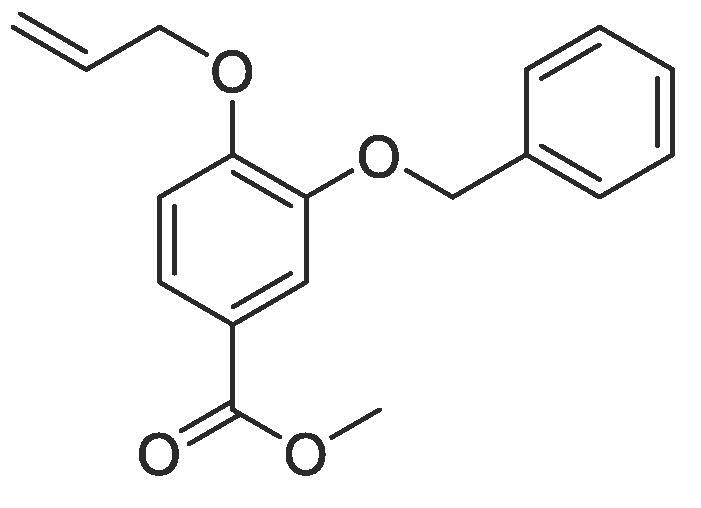

Methyl 4-(allyloxy)-3-hydroxybenzoate (0.104 g, 0.500 mmol), benzyl bromide (0.297 mL, 2.50 mmol), and K_2_CO_3_ (0.553 g, 0.400 mmol) were added to anhydrous DMF (2.5 mL) and stirred at r.t. for 6 h. The reaction mixture was diluted with ethyl acetate (100 mL) and the combined organic layers washed with H_2_O (3 × 100 mL) and brine (3 × 100 mL), dried over MgSO_4_, filtered and concentrated *in vacuo* affording the title compound as a light-yellow oil (0.115 g, 0.385 mmol, 77%); *R_f_* = 0.70 (1:5 EtOAc: *n*-Pentane); ^1^H NMR (400 MHz, CDCl_3_) δ_H_: 7.70–7.66 (m, Ar***H***, 2H), 7.43–7.32 (m, Ar***H***, 5H), 6.93 (d, Ar***H***, *J* = 8, 1H), 6.14–6.07 (m, C***H*** = CH_2_, 1H), 5.46 (dq, C***H_2_*** = CH, *J* = 16, 4, 1H), 5.33 (dq, C***H_2_*** = CH, *J* = 8, 4, 1H), 5.20 (s, C***H_2_***OAr, 2H), 4.70 (dt, C***H_2_***OAr, *J* = 8, 4, 2H), 3.90 (s, COOC***H_3_***, 3H); ^13^C NMR (125 MHz, CDCl_3_) δ_C_: 166.9 (***C***=O), 160.9 (***C***OCH_2_), 148.3 (***C***OCH_2_Ar), 135.3 (***C***CH_2_), 132.9 (***C***H = CH_2_), 128.7 (Ar***C***), 128.7 (Ar***C***), 128.5 (Ar***C***), 127.8 (***C***COOCH_3_), 127.5 (Ar***C***), 127.1 (Ar***C***), 123.0 (Ar***C***), 118.0 (***C***H_2_ = CH), 115.4 (Ar***C***), 112.8 (Ar***C***), 71.3 (***C***H_2_Ar), 69.8 (***C***H_2_OAr), 52.0 (***C***H_3_COO); *ν*max/cm-1 (solid state) = 1714 (CO, s), 1468 (C=C), 1267 (C—O); ESI-HRMS found *m*/*z* 299.1272 [M + H]+, C17H19O9 requires 299.1283.

#### 4.2.9 Compound 9–4-(allyloxy)-3-(benzyloxy)benzoic acid

**Figure:**
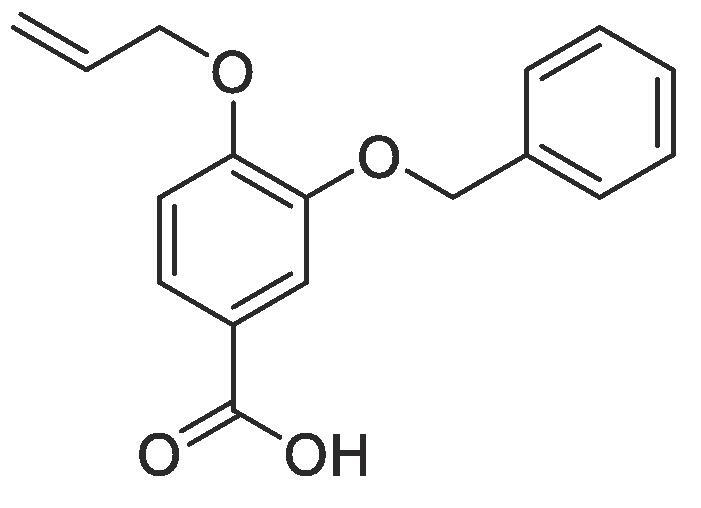

Methyl 4-(allyloxy)-3-(benzyloxy)benzoate (0.115 g, 0.385 mmol) was added to 2 M NaOH (0.578 mL, 1.16 mmol) and MeOH (0.578 mL) and the reaction mixture stirred at 50 °C for 1 h. The reaction was acidified to pH 2–3 by drop-wise addition of 2 M HCl. The product was extracted with ethyl acetate (2 × 50 mL) and the combined organic layers were dried over MgSO_4_, filtered and concentrated *in vacuo* affording the title compound as a light-yellow solid (0.090 g, 0.316 mmol, 82%); *R_f_* = 0.00 (1:5 EtOAc:*n*-Pentane), mp = 124–125 °C, ^1^H NMR (400 MHz, DMSO‑*d*_6_) δ_H_: 7.56–7.54 (m, Ar***H***, 2H), 7.47–7.33 (m, Ar***H***, 5H), 7.08 (d, Ar***H***, *J* = 8, 1H), 6.09–6.02 (m, C***H*** = CH_2_, 1H), 5.42 (d, C***H_2_*** = CH, *J* = 16, 1H), 5.27 (d, C***H_2_*** = CH, *J* = 8, 1H), 5.15 (s, C***H_2_***OAr, 2H), 4.67 (d, C***H_2_***OAr, *J* = 4, 2H). ^13^C NMR (125 MHz, DMSO‑*d*_6_) δC: 167.0 (***C***=O), 159.2 (***C***OCH_2_), 147.5 (***C***OCH_2_Ar), 137.0 (***C***CH_2_), 133.4 (***C***H = CH_2_), 128.4 (Ar***C***), 127.8 (Ar***C***), 127.5 (***C***COOH), 123.5 (Ar***C***), 123.3 (Ar***C***), 117.7 (***C***H_2_ = CH), 114.4 (Ar***C***), 112.8 (Ar***C***), 70.0 (***C***H_2_Ar), 69.8 (***C***H_2_OAr); *ν*_max_/cm^−1^ (solid state) = 2903 (OH, br), 1674 (CO, s), 1436 (C=C), 1271 (C—O); ESI-HRMS found *m*/*z* 285.1121 [M + H]^+^, C_17_H_17_O_4_ requires 285.1127.

#### 4.2.10 Compound 10 – Methyl 4-(allyloxy)-3-((methylthio)methoxy)benzoate

**Figure:**
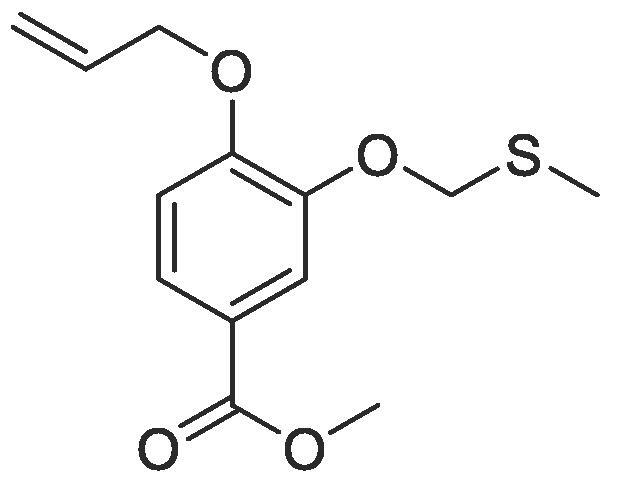

DIAD (0.491 mL, 2.5 mmol) and PPh_3_ (0.656 g, 2.5 mmol) were added to THF (2.5 mL) at 0 °C and stirred for 5 min, before addition of 2-(methylthio)ethan-1-ol (0.130 mL, 1.50 mmol) followed and further stirring occurred for 10 min. Methyl 4-(allyloxy)-3-hydroxybenzoate (0.104 g, 0.5 mmol) in THF (0.75 mL) was added and the mixture stirred at 0 °C for 0.5 h before warming to r.t. and stirring for a further 6 h. The reaction mixture was concentrated *in vacuo* and the crude product purified by column chromatography (1:5 EtOAc:*n*-Pentane) affording the title compound as a white crystalline solid (0.120 g, 0.425 mmol, 85%); *R_f_* = 0.60 (1:5 EtOAc:*n*-Pentane); ^1^H NMR (400 MHz, CDCl_3_) δ_H_: 7.59 (dd, Ar***H***, *J* = 12, 4, 1H), 7.50 (d, Ar***H***, *J* = 4, 1H), 6.83 (d, Ar***H***, *J* = 8, 1H), 6.04–5.97 (m, C***H*** = CH_2_, 1H), 5.37 (dq, C***H_2_*** = CH, *J* = 16, 4, 1H), 5.24 (dq, C***H_2_*** = CH, *J* = 8, 4, 1H), 4.57 (dt, C**H_2_**OAr, *J* = 8, 4, 2H), 4.19 (t, C***H_2_***CH_2_, *J* = 8, 2H), 3.82 (s, COOC***H_3_***, 3H), 2.87 (t, C***H_2_***S, J = 8, 2H), 2.19 (s, SC***H_3_***, 3H); ^13^C NMR (125 MHz, CDCl_3_) δ_C_: 166.7 (***C***=O), 152.4 (***C***OCH_2_), 147.9 (***C***OCH_2_CH_2_), 132.6 (***C***H = CH_2_), 123.8 (***C***COOCH_3_), 122.8 (Ar***C***), 118.0 (***C***H_2_ = CH), 114.3 (Ar***C***), 112.4 (Ar***C***), 69.5 (***C***H_2_CH_2_), 68.8 (***C***H_2_OAr), 51.9 (***C***H_3_COO), 32.8 (***C***H_2_S), 16.3 (***C***H_3_S); *ν*_max_/cm^−1^ (solid state) = 1713 (CO, s), 1422 (C=C), 1266 (C—O); ESI found *m*/*z* 283.0999 [M + H]^+^, C_14_H_19_O_4_S requires 283.0999.

#### 4.2.11 Compound 11–4-(allyloxy)-3-((methylthio)methoxy)benzoic acid

**Figure:**
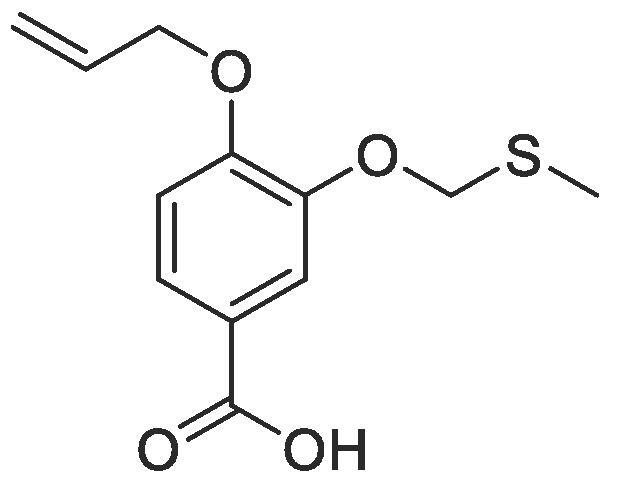

Methyl 4-(allyloxy)-3-((methylthio)methoxy)benzoate (0.120 g, 0.425 mmol) was added to 2 M NaOH (0.640 mL, 1.28 mmol) and MeOH (0.640 mL) and the reaction mixture stirred at 50 °C for 1 h. The reaction was acidified to pH 2–3 by drop-wise addition of 2 M HCl. The product was extracted with ethyl acetate (2 × 50 mL) and the combined organic layers were dried over MgSO_4_, filtered and concentrated *in vacuo* affording the title compound as a white solid (0.102 g, 0.383 mmol, 90%); *R_f_* = 0.00 (1:5 EtOAc:*n*-Pentane); mp 120–121 °C; ^1^H NMR (400 MHz, DMSO‑*d*_6_) δ_H_: 7.56 (dd, Ar***H***, *J* = 16, 4 1H), 7.47 (d, Ar***H***, *J* = 4, 1H), 7.07 (d Ar***H***, *J* = 8, 1H), 6.08–6.01 (m, C***H*** = CH_2_, 1H), 5.43 (dq, C***H_2_*** = CH, *J* = 16, 4, 1H), 5.28 (dq, C***H_2_*** = CH, *J* = 8, 4, 1H), 4.65 (d, C***H_2_***OAr, *J* = 8, 2H), 4.19 (t, C***H_2_***CH_2_, *J* = 8, 2H), 2.86 (t, C***H_2_***S, *J* = 8, 2H), 2.19 (s, SC***H_3_***, 3H); ^13^C NMR (125 MHz, DMSO‑*d*_6_) δ_C_: 167.5 (***C***=O), 152.2 (***C***OCH_2_), 147.9 (***C***OCH_2_CH_2_), 133.8 (***C***H = CH_2_), 123.9 (***C***COOH), 123.6 (Ar***C***), 118.2 (***C***H2 = CH), 114.3 (Ar***C***), 113.2 (Ar***C***), 69.3 (***C***H_2_CH_2_), 68.9 (***C***H_2_OAr), 32.7 (***C***H_2_S), 16.0 (***C***H_3_S); *ν*_max_/cm^−1^ (solid state) = 2938 (OH, br), 1667 (CO, s), 1432 (C=C), 1270 (C—O); ESI found *m*/*z* 267.0687 [M−H]^-^, C_13_H_15_O_3_S requires 267.0691.

#### 4.2.12 Compound 12 - Methyl 4-(allyloxy)-3-(2-(*tert*-butoxyl)-2-oxoethoxy)benzoate

**Figure:**
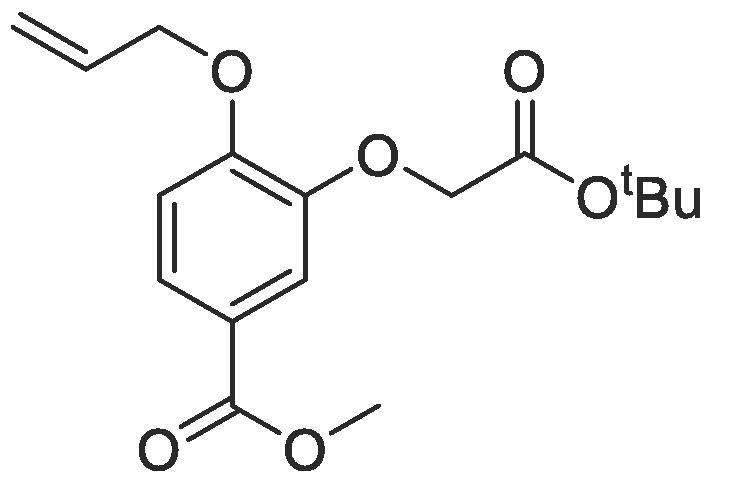

Methyl 4-(allyloxy)-3-hydroxybenzoate (0.104 g, 0.500 mmol), *tert*-Butyl bromoacetate (0.369 mL, 2.50 mmol), and K_2_CO_3_ (0.553 g, 0.400 mmol) were added to anhydrous DMF (2.5 mL) and stirred at r.t. for 6 h. The reaction mixture was diluted with ethyl acetate (100 mL) and the combined organic layers washed with H_2_O (3 × 100 mL) and brine (3 × 100 mL), dried over MgSO_4_, filtered and concentrated *in vacuo*. The crude mixture was purified by column chromatography (1:5 EtOAc:*n*-Pentane) affording the title compound as a colourless oil (0.119 g, 0.370 mmol, 74%); *R_f_* = 0.35 (1:5 EtOAc:*n*-Pentane); ^1^H NMR (400 MHz, CDCl_3_) δ_H_: 7.67 (dd, Ar***H***, *J* = 8, 4, 1H), 7.48 (d, Ar***H***, *J* = 4, 1H), 6.91 (d, Ar***H***, *J* = 8, 1H), 6.11–6.04 (m, C***H*** = CH_2_, 1H), 5.44 (dq, C***H_2_*** = CH, *J* = 16, 4, 1H), 5.31 (dq, C***H_2_*** = CH, *J* = 8, 4, 1H), 4.68 (d, C***H_2_***OAr, *J* = 4, 2H), 4.63 (s, C***H_2_***COO, 2H), 3.87 (s, COOC***H_3_***, 3H), 1.49 (s, (C***H_3_***)_3_CO, 9H); ^13^C NMR (125 MHz, CDCl_3_) δ_C_: 167.6 (***C***=O), 166.6 (***C***=O), 152.4 (***C***OCH_2_), 147.2 (***C***OCH_2_C = O), 132.6 (***C***H = CH_2_), 124.4 (***C***COOCH_3_), 122.7 (Ar***C***), 118.3 (***C***H_2_ = CH), 114.7 (Ar***C***), 112.7 (Ar***C***), 82.4 (***C***(CH_3_)_3_), 69.7 (***C***H_2_OAr), 66.5 (***C***H_2_C = O), 51.9 (***C***H_3_COO), 28.0 ((***C***H_3_)C); *ν*_max_/cm^−1^ (solid state) = 1718 (CO, s), 1368 (C=C), 1130 (C—O); ESI found *m*/*z* 345.1318 [M + Na^+^], C_17_H_22_O_6_Na requires 345.1314.

#### 4.2.13 Compound 13 – 4-(allyloxy)-3-(carboxymethoxy)benzoic acid

**Figure:**
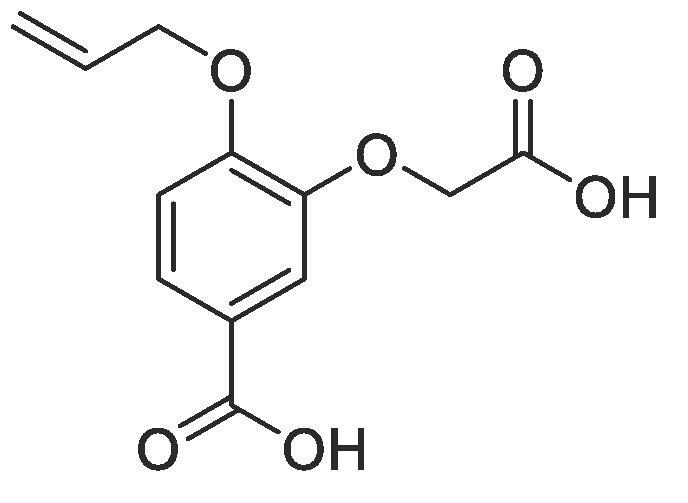

Methyl 4-(allyloxy)-3-(2-(*tert*-butoxyl)-2-oxoethoxy)benzoate (0.119 g, 0.370 mmol) was added to 2 M NaOH (0.555 mL, 1.11 mmol) and MeOH (0.555 mL) and the reaction mixture stirred at 50 °C for 1 h. The reaction was acidified to pH 2–3 by drop-wise addition of 2 M HCl. The product was extracted with ethyl acetate (2 × 50 mL) and the combined organic layers were dried over MgSO_4_, filtered and concentrated *in vacuo* affording the title compound as a yellow solid (0.102 g, 0.383 mmol, 90%); *R_f_* = 0.00 (1:5 EtOAc:*n*-Pentane); mp = 180–181 °C; ^1^H NMR (400 MHz, DMSO‑*d*_6_) δ_H_: 7.56 (dd, Ar***H***, *J* = 8, 4, 1H), 7.34 (d, Ar***H***, *J* = 4, 1H), 7.08 (d, Ar***H***, *J* = 8, 1H), 6.10–6.03 (m, C***H*** = CH_2_, 1H), 5.43 (dq, C***H_2_*** = CH, *J* = 16, 4, 1H), 5.28 (dq, C***H_2_*** = CH, *J* = 8, 4, 1H), 4.74 (s, C***H_2_***COO, 2H), 4.67 (d, C***H_2_***OAr, *J* = 8, 2H); ^13^C NMR (125 MHz, DMSO‑*d*_6_) δ_C_: 170.1 (***C***=O), 169.6 (***C***=O), 158.9 (***C***OCH_2_), 146.9 (***C***OCH_2_COOH), 133.2 (***C***H = CH_2_), 123.6 (***C***COOH), 123.0 (Ar***C***), 117.9 (***C***H_2_ = CH), 113.7 (Ar***C***), 112.8 (Ar***C***), 68.9 (***C***H_2_OAr), 65.0 (***C***H_2_COOH),; *ν*_max_/cm^−1^ (solid state) = 2938 (OH, br), 1687 (CO, s), 1429 (C=C), 1209 (C—O); ESI found *m*/*z* 275.1175 [M + Na]^+^, C_12_H_12_O_6_Na requires 275.06.

#### 4.2.14 Compound 14 – Methyl 4-(allyloxy)-3-(4-((*tert*-butoxycarbonyl)amino)butoxy)benzoate

**Figure:**
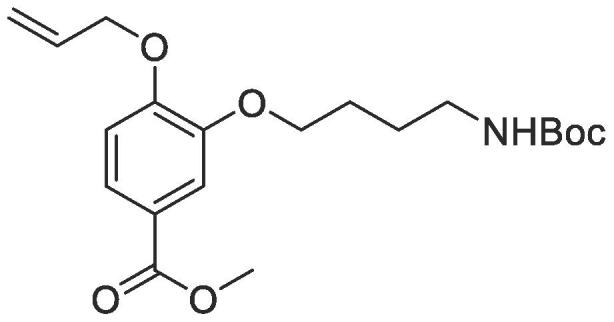

Methyl 4-(allyloxy)-3-hydroxybenzoate (0.104 g, 0.500 mmol), 4-(Boc-amino)butyl bromide (0.630 g, 2.50 mmol), and K_2_CO_3_ (0.553 g, 0.400 mmol) were added to anhydrous DMF (2.5 mL) and stirred at r.t. for 6 h.. The reaction mixture was diluted with ethyl acetate (100 mL) and the combined organic layers washed with H_2_O (3 × 100 mL) and brine (3 × 100 mL), dried over MgSO_4_, filtered and concentrated *in vacuo* affording the title compound as a colourless oil which was taken forward as crude (0.140 g, 0.415 mmol, 83%); *R_f_* = 0.11 (1:5 EtOAc:*n*-Pentane); ^1^H NMR (400 MHz, CDCl_3_) δ_H_: 7.63 (dd, Ar***H***, *J* = 12, 4, 1H), 7.52 (d, Ar***H***, *J* = 4, 1H), 6.87 (d, Ar***H***, *J* = 8, 1H), 6.11–6.04 (m, C***H*** = CH_2_, 1H), 5.40 (dq, C***H_2_*** = CH, *J* = 16, 4, 1H), 5.31 (dq, C***H_2_*** = CH, *J* = 8, 4, 1H), 4.64 (d, C***H_2_***OAr, *J* = 8, 2H), 4.07 (t, ArOC***H_2_***CH_2_, *J* = 8, 2H), 3.88 (s, COOC***H_3_***, 3H), 3.42 (t, C***H_2_***NH, *J* = 8, 2H), 1.90–1.82 (m, ArOCH_2_C***H_2_***, 2H), 1.65–1.61 (m, C***H_2_***CH_2_NH, 2H), 1.46 (C***H_3_***)_3_CO, 9H); ^13^C NMR (125 MHz, CDCl_3_) δ_C_: 166.9 (***C***=O), 156.1 (***C***=O), 152.4 (***C***OCH_2_), 148.4 (***C***OCH_2_CH_2_), 132.8 (***C***H = CH_2_), 123.6 (***C***COOCH_3_), 122.9 (Ar***C***), 118.1 (***C***H_2_ = CH), 114.0 (Ar***C***), 112.4 (Ar***C***), 79.0 (***C***(CH_3_)_3_), 69.7 (***C***H_2_OAr), 68.9 (O***C***H_2_CH_2_), 52.0 (***C***H_3_COO), 33.3 (***C***H_2_NH), 28.9 ((***C***H_3_)C), 28.7 (OCH_2_***C***H_2_), 28.5 (OCH_2_CH_2_***C***H_2_); ESI-HRMS found *m*/*z* 402.1888 [M + Na]^+^, C_20_H_29_NO_6_Na requires 402.2000.

#### 4.2.15 Compound 15–4-(allyloxy)-3-(4-((*tert*-butoxycarbonyl)amino)butoxy)benzoic acid

**Figure:**
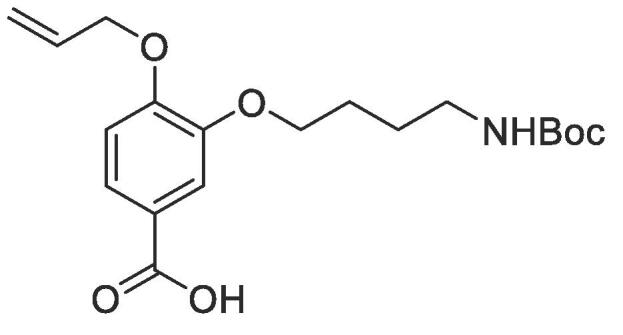

Methyl 4-(allyloxy)-3-(4-((*tert*-butoxycarbonyl)amino)butoxy)benzoate (0.140 g, 0.415 mmol) was added to 2 M NaOH (0.555 mL, 1.11 mmol) and MeOH (0.555 mL) and the reaction mixture stirred at 50 °C for 1 h. The reaction was acidified to pH 2–3 by drop-wise addition of 2 M HCl. The product was extracted with ethyl acetate (2 × 50 mL) and the combined organic layers were dried over MgSO_4_, filtered and concentrated *in vacuo.* The crude mixture was purified by column chromatography (1:3 EtOAc:*n*-Pentane) affording the title compound as a white solid (0.102 g, 0.136 g, 0.374 mmol, 90%); *R_f_* = 0.00 (1:5 EtOAc:*n*-Pentane); ^1^H NMR (400 MHz, DMSO‑*d*_6_) δ_H_: 7.53 (dd, Ar***H***, *J* = 8, 4, 1H), 7.45 (d, Ar***H***, *J* = 4, 1H), 7.05 (d, Ar***H***, *J* = 8, 1H), 6.10–6.00 (m, C***H*** = CH_2_, 1H), 5.42 (dq, C***H_2_*** = CH, *J* = 16, 4, 1H), 5.28 (dq, C***H_2_*** = CH, *J* = 8, 4, 1H), 4.65 (dt, C***H_2_***OAr, *J* = 8, 4, 2H), 4.00 (t, ArOC***H_2_***CH_2_, *J* = 8, 2H), 2.98 (q, C***H_2_***NH, *J* = 8, 2H), 1.74–1.70 (m, ArOCH_2_C***H_2_***, 2H), 1.56–1.54 (m, C***H_2_***CH_2_NH, 2H), 1.38 (C***H_3_***)_3_CO, 9H); ^13^C NMR (125 MHz, DMSO‑*d*_6_) δ_C_: 167.7 (***C***=O), 156.2 (***C***=O), 153.5 (***C***OCH_2_), 148.5 (***C***OCH_2_CH_2_), 134.0 (***C***H = CH_2_), 123.7 (***C***COOH), 123.0 (Ar***C***), 118.1 (***C***H_2_ = CH), 114.2 (Ar***C***), 113.3 (Ar***C***), 78.0 (***C***(CH_3_)_3_), 69.4 (***C***H_2_OAr), 68.7 (O***C***H_2_CH_2_), 49.2 (***C***H_2_NH), 31.3 ((***C***H_3_)C), 28.9 (OCH_2_***C***H_2_), 26.7 (OCH_2_CH_2_***C***H_2_); *ν*_max_/cm^−1^ (solid state) = 3368 (N—H), 2989 (OH, br), 1682 (CO, s), 1441 (C=C), 1263 (C—O); ESI-HRMS found *m*/*z* 364.1755 [M−H]^-^, C_19_H_26_NO_6_ requires 364.1760.

#### 4.2.16 Compound 16 – Methyl 4-(allyloxy)-3-(2-amino-2-oxoethoxy)benzoate

**Figure:**
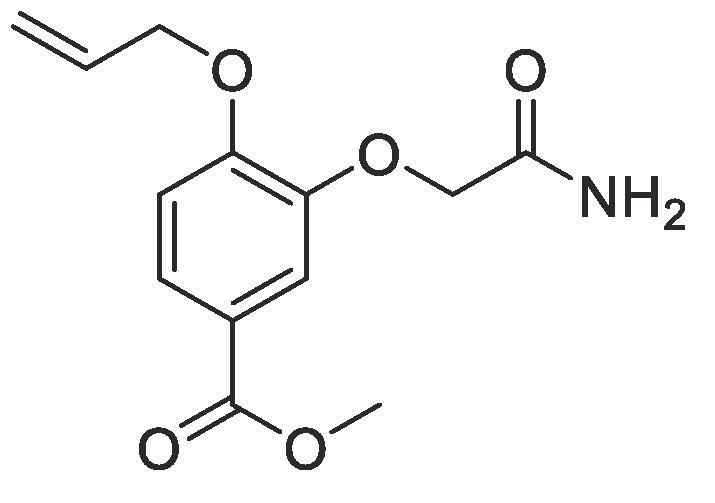

Methyl 4-(allyloxy)-3-hydroxybenzoate (0.104 g, 0.500 mmol), 2-bromoacetamide (0.345 g, 2.50 mmol), and K_2_CO_3_ (0.553 g, 0.400 mmol) were added to anhydrous DMF (2.5 mL) and stirred at r.t. for 6 h. The reaction mixture was diluted with ethyl acetate (100 mL) and the combined organic layers washed with H_2_O (3 × 100 mL) and brine (3 × 100 mL), dried over MgSO_4_, filtered and concentrated *in vacuo* affording the title compound as a white solid (0.106 g, 0.400 mmol, 80%); *R_f_* = 0.00 (1:5 EtOAc:*n*-Pentane); ^1^H NMR (400 MHz, CDCl_3_) δ_H_: 7.75 (dd, Ar***H***, *J* = 8, 4, 1H), 7.60 (d, Ar***H***, *J* = 4, 1H), 6.94 (d, Ar***H***, *J* = 12, 1H), 6.10–6.02 (m, C***H*** = CH_2_, 1H), 5.44 (dq, C***H_2_*** = CH, *J* = 16, 4, 1H), 5.35 (dq, C***H_2_*** = CH, *J* = 8, 4, 1H), 4.68 (dt, C***H_2_***OAr, *J* = 8, 4, 2H), 4.58 (s, C***H_2_***CONH_2_, 2H), 3.89 (s, COOC***H_3_***, 3H); ^13^C NMR (125 MHz, CDCl_3_) δ_C_: 170.3 (***C***ONH_2_), 166.3 (***C***=O), 152.6 (***C***OCH_2_), 146.9 (***C***OCH_2_CO), 132.2 (***C***H = CH_2_), 125.5 (***C***COOCH_3_), 123.3 (Ar***C***), 118.7 (***C***H_2_ = CH), 116.5 (Ar***C***), 112.5 (Ar***C***), 69.6 (***C***H_2_OAr), 69.2 (***C***H_2_CO), 52.1 (***C***H_3_COO); *ν*_max_/cm^−1^ (solid state) = 3462 (N—H), 1700 (CO, s), 1426 (C=C), 1272 (C—O); ESI-HRMS found *m*/*z* 266.1022 [M + H]^+^, C_13_H_16_NO_5_ requires 266.1028.

#### 4.2.17 Compound 17–4-(allyloxy)-3-(2-amino-2-oxoethoxy)benzoic acid

**Figure:**
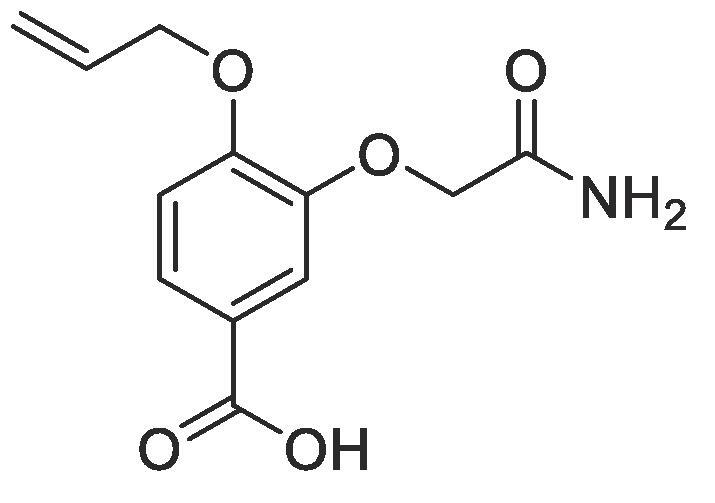

Methyl 4-(allyloxy)-3-(4-((*tert*-butoxycarbonyl)amino)butoxy)benzoate (0.106 g, 0.400 mmol) was added to LiOH·H_2_O (0.017 g, 0.400) in MeOH (0.555 mL) and the reaction mixture stirred at r.t. for 3 h. The reaction was acidified to pH 2–3 by drop-wise addition of 2 M HCl. The product was extracted with ethyl acetate (2 × 50 mL) and the combined organic layers were dried over MgSO_4_, filtered and concentrated *in vacuo* affording the title compound as a yellow solid (0.088 g, 0.352 mmol, 88%); *R_f_* = 0.00 (1:5 EtOAc:*n*-Pentane); mp = 121–122 °C; ^1^H NMR (400 MHz, DMSO‑*d*_6_) δ_H_: 7.55 (dd, Ar***H***, *J* = 8, 4, 1H), 7.35 (d, Ar***H***, *J* = 4, 1H), 7.08 (d, Ar***H***, *J* = 12, 1H), 6.10–6.03 (m, C***H*** = CH_2_, 1H), 5.44 (dq, C***H_2_*** = CH, *J* = 16, 4, 1H), 5.29 (dq, C***H_2_*** = CH, *J* = 8, 4, 1H), 4.71 (s, C***H_2_***CONH_2_, 2H), 4.67 (d, C***H_2_***OAr, *J* = 4, 2H); ^13^C NMR (125 MHz, CDCl_3_) δ_C_: 170.2 (***C***ONH_2_), 168.7 (***C***=O), 152.0 (***C***OCH_2_), 149.4 (***C***OCH_2_CO), 133.8 (***C***H = CH_2_), 126.4 (***C***COOH), 125.4 (Ar***C***), 118.4 (***C***H_2_ = CH), 116.9 (Ar***C***), 113.4 (Ar***C***), 72.5 (***C***H_2_OAr), 69.4 (***C***H_2_CO); *ν*_max_/cm^−1^ (solid state) = 2928 (OH, br), 1668 (CO, s), 1449 (C=C), 1221 (C—O); ESI-HRMS found *m*/*z* 250.0720 [M−H]^-^, C_12_H_12_NO_5_ requires 250.0715.

#### 4.2.18 Compound 18 – Methyl 4-(allyloxy)-3-(2-(methoxymethoxy)ethoxy)benzoate

**Figure:**
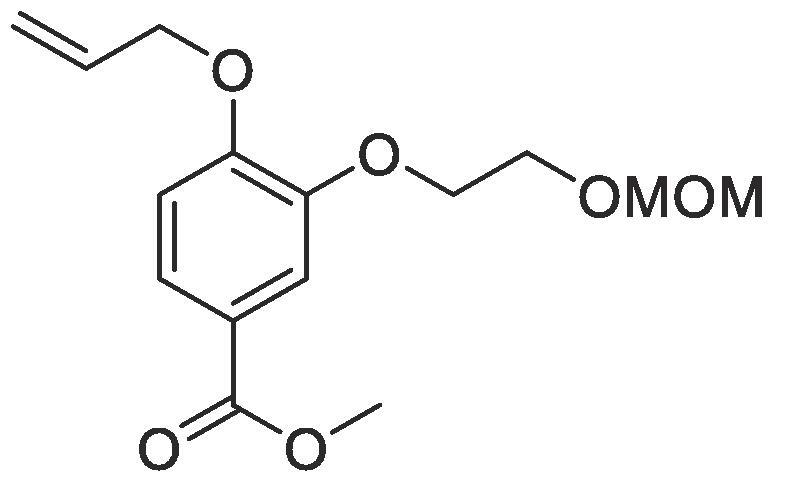

Methyl 4-(allyloxy)-3-hydroxybenzoate (0.104 g, 0.500 mmol), 1-bromo-2-(methoxymethoxy)ethane (0.292 mL, 2.50 mmol), and K_2_CO_3_ (0.553 g, 0.400 mmol) were added to anhydrous DMF (2.5 mL) and stirred at r.t. for 6 h. The reaction mixture was diluted with ethyl acetate (100 mL) and the combined organic layers washed with H_2_O (3 × 100 mL) and brine (3 × 100 mL), dried over MgSO_4_, filtered and concentrated *in vacuo* affording the title compound as a colourless oil which was taken forward as crude (0.124 g, 0.420 mmol, 84%); *R_f_* = 0.28 (1:5 EtOAc:*n*-Pentane); ^1^H NMR (400 MHz, CDCl_3_) δ_H_: 7.50 (dd, Ar***H***, *J* = 8, 4, 1H), 7.44 (d, Ar***H***, *J* = 4, 1H), 6.75 (d, Ar***H****, J* = 12, 1H), 5.97–5.88 (m, C***H*** = CH_2_, 2H), 5.30 (dq, C***H_2_*** = CH, *J* = 16, 4, 1H), 5.17–5.13 (m, C***H_2_*** = CH, 1H), 4.57 (s, OC***H_2_***OCH_3_, 2H), 4.51–4.48 (m, C***H_2_***OAr, 2H), 4.11–4.08 (m, CH_2_C***H_2_***, 2H), 3.79–3.78 (m, C***H_2_***CH_2_, 2H), 3.73 (s, COOC***H_3_***, 3H), 3.25 (s, C***H_3_***OCH_2_, 3H); ^13^C NMR (125 MHz, CDCl_3_) δ_C_: 166.7 (***C***=O), 152.6 (***C***OCH_2_), 148.2 (***C***OCH_2_CH_2_), 132.7 (***C***H = CH_2_), 123.9 (***C***COOCH_3_), 122.8 (Ar***C***), 118.0 (***C***H_2_ = CH), 114.6 (Ar***C***), 112.4 (Ar***C***), 96.5 (***C***H_2_OCH_3_), 69.5 (***C***H_2_OAr), 68.1 (***C***H_2_CH_2_), 65.7 (***C***H_2_O), 55.2 (***C***H_3_O), 51.9 (***C***H_3_COO); *ν*_max_/cm^−1^ (solid state) = 1713 (CO, s), 1468 (C=C), 1209 (C—O); ESI-HRMS found *m*/*z* 297.1331 [M + H]^+^, C_15_H_21_O_6_ requires 297.1338.

#### 4.2.19 Compound 19 –4-(allyloxy)-3-(2-(methoxymethoxy)ethoxy)benzoic acid

**Figure:**
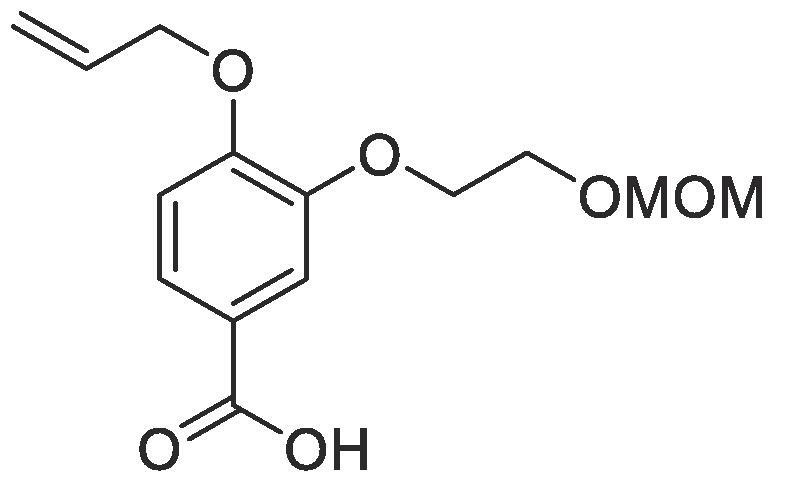

Methyl 4-(allyloxy)-3-(2-(methoxymethoxy)ethoxy)benzoate (0.124 g, 0.420 mmol) was added to 2 M NaOH (0.630 mL, 1.26 mmol) and MeOH (0.630 mL) and the reaction mixture stirred at 50 °C for 1 h. The reaction was acidified to pH 2–3 by drop-wise addition of 2 M HCl. The product was extracted with ethyl acetate (2 × 50 mL) and the combined organic layers were dried over MgSO_4_, filtered and concentrated *in vacuo* affording the title compound as a white solid (0.102 g, 0.370 mmol, 88%); *R_f_* = 0.00 (1:5 EtOAc:*n*-Pentane); mp = 87–88 °C, ^1^H NMR (400 MHz, DMSO‑*d*_6_) δ_H_: 7.54 (dd, Ar***H***, *J* = 8, 4, 1H), 7.46 (d, Ar***H***, *J* = 4, 1H), 7.05 (d, Ar***H****, J* = 12, 1H), 6.08–6.00 (m, C***H*** = CH_2_, 2H), 5.42 (dq, C***H_2_*** = CH, *J* = 16, 4, 1H), 5.27 (dq, C***H_2_*** = CH, *J* = 8, 4, 1H), 4.65–4.63 (m, C***H_2_***OAr, 2H), 4.17–4.13 (m, OC***H_2_***OCH_3_, 2H), 3.86–3.78 (m, CH_2_C***H_2_***, 2H), 3.62–3.59 (m, C***H_2_***CH_2_, 2H), 3.27 (s, C***H_3_***OCH_2_, 3H); ^13^C NMR (125 MHz, DMSO‑*d*_6_) δ_C_: 167.5 (***C***=O), 154.1 (***C***OCH_2_), 148.1 (***C***OCH_2_CH_2_), 133.8 (***C***H = CH_2_), 123.8 (***C***COOH), 122.6 (Ar***C***), 118.2 (***C***H_2_ = CH), 114.4 (Ar***C***), 113.2 (Ar***C***), 96.1 (***C***H_2_OCH_3_), 69.3 (***C***H_2_OAr), 68.6 (***C***H_2_CH_2_), 65.8 (***C***H_2_O), 56.3 (***C***H_3_O); *ν*_max_/cm^−1^ (solid state) = 2930 (OH, br), 1671 (CO, s), 1445 (C=C), 1227 (C—O); ESI-HRMS found *m*/*z* 305.0992 [M + Na]^+^, C_14_H_18_O_6_Na requires 305.1001.

#### 4.2.20 Compound 20 – Methyl 4-(allyloxy)-3-((1-trityl-1*H*-imidazol-4-yl)methoxy)benzoate

**Figure:**
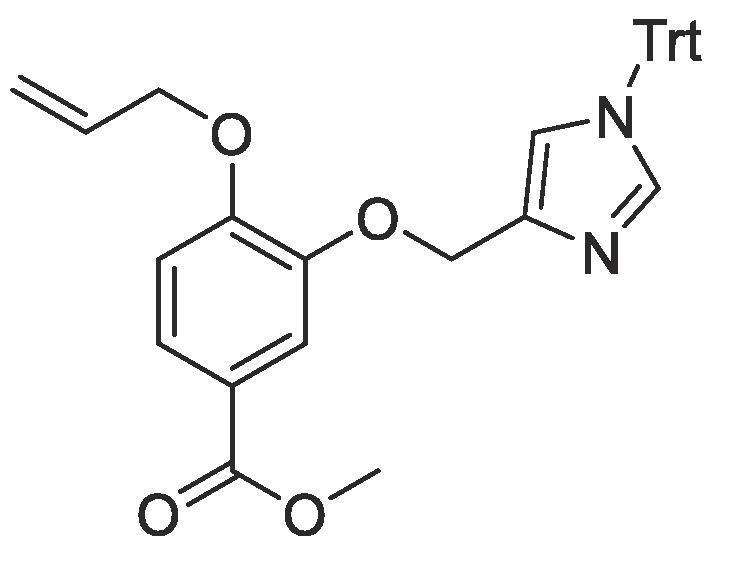

Trityl-imidazole-OH (1 g, 2.93 mmol) was suspended in DCM (15 mL) and PPh_3_ (925 mg, 3.52 mmol) was added and the reaction mixture cooled on ice. *N*-bromosuccinimide (627 mg, 3.52 mmol) was then added portion-wise and the reaction mixture stirred for 2 h at 0 °C. The reaction mixture was quenched with sat. NaHCO_3_ (10 mL) and the product extracted with DCM (20 mL) and concentrated *in vacuo* to afford crude 4-(bromomethyl)-1-trityl-1*H*-imidazole which was used without further purification.

Methyl 4-(allyloxy)-3-hydroxybenzoate (0.122 g, 0.59 mmol), the crude 4-(bromomethyl)-1-trityl-1*H*-imidazole, and K_2_CO_3_ (1.26 g, 4.7 mmol) were added to anhydrous DMF (5 mL) and stirred at r.t. for overnight. The reaction mixture was diluted with ethyl acetate (100 mL) and the combined organic layers washed with H_2_O (3 × 100 mL) and brine (3 × 100 mL), dried over MgSO_4_, filtered and concentrated *in vacuo.* The crude mixture was purified by column chromatography (hexane:EtOAc 10:0 → 1:1) affording the title compound as a yellow oil (0.24 g, 0.46 mmol, 78%); *R_f_* = 0.70 (1:5 EtOAc:*n*-Pentane); ^1^H NMR (400 MHz, CDCl_3_) δ_H_: 7.68 (d, Ar***H***, *J* = 4, 1H), 7.64 (dd, Ar***H***, *J* = 8, 4, 1H), 7.42 (d, Ar***H***, *J* = 4, 1H), 7.33–7.30 (m, Ar***H****,* 8H), 7.12–7.09 (m, Ar***H****,* 4H), 6.85 (d, Ar***H****, J* = 8, 1H), 6.02–5.95 (m, C***H*** = CH_2_, 1H), 5.34 (dd, C***H_2_*** = CH, *J* = 16, 4, 1H), 5.22 (dd, C***H_2_*** = CH, *J* = 8, 4, 1H), 5.14 (s, OC***H_2_***Ar, 2H), 4.58 (d, C***H_2_***OAr, *J* = 4, 2H), 4.23 (s, COOC***H_3_***, 3H); ^13^C NMR (125 MHz, CDCl_3_) δC: 166.9 (***C***=O), 153.0 (***C***OCH_2_), 147.9 (***C***OCH_2_CO), 142.7, 142.4, 142.3 (Ar***C***), 139.1 (Ar***C***), 137.0 (Ar***C***), 132.9 (***C***H = CH_2_), 129.9 (Ar***C***), 128.2 (Ar***C***), 124.1 (***C***COOH), 122.9 (Ar***C***), 120.8 (Ar***C***), 118.2 (***C***H_2_ = CH), 116.1 (Ar***C***), 112.7 (Ar***C***), 75.5 (Ar***C***), 69.7 (***C***H_2_OAr), 66.2 (***C***H_2_CO) 53.1 (***C***H_3_); ESI-HRMS found *m*/*z* 531.2293 [M + H]^+^, C_34_H_31_N_2_O_4_ requires 531.2284.

#### 4.2.21 Compound 21 – 4-(allyloxy)-3-((1-trityl-1*H*-imidazol-4-yl)methoxy)benzoic acid

**Figure:**
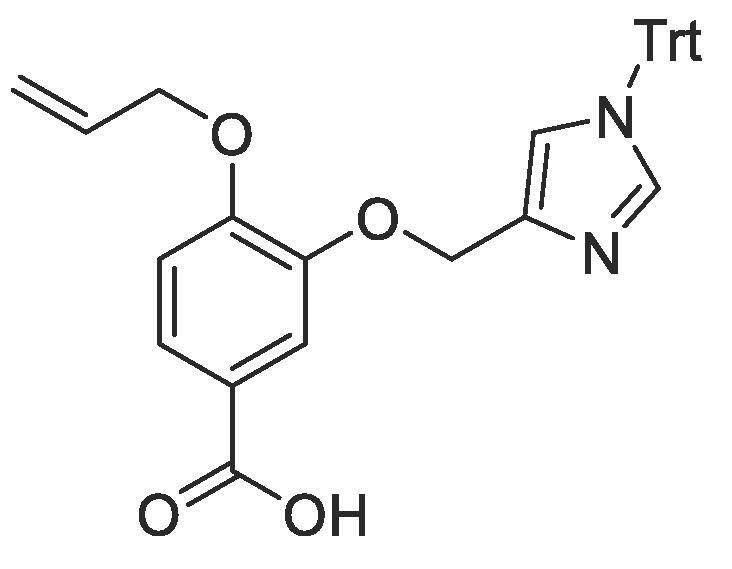

Methyl 4-(allyloxy)-3-((1-trityl-1*H*-imidazol-4-yl)methoxy)benzoate (0.1 g, 0.19 mmol) was added to 2 M NaOH (3 mL) and MeOH (4 mL) and the reaction mixture stirred at 50 °C overnight. The reaction was acidified to pH 2–3 by drop-wise addition of 2 M HCl. The product was extracted with ethyl acetate (2 × 50 mL) and the combined organic layers were dried over MgSO_4_, filtered and concentrated *in vacuo* affording the title compound as an off-white solid (0.82 g, 0.16 mmol, 84%); *R_f_* = 0.00 (1:5 EtOAc:*n*-Pentane); ^1^H NMR (400 MHz, CDCl_3_) δ_H_: 7.80–7.70 (m, Ar***H***, *J* = 4, 2H), 7.42–7.28 (m, Ar***H****,* 8H), 7.09–7.08 (m, Ar***H****,* 8H), 6.94 (s, Ar***H***, 1H), 6.86 (d, Ar***H****, J* = 8, 1H), 5.99–5.92 (m, C***H*** = CH_2_, 1H), 5.34–5.19 (m, C***H_2_*** = CH, OC***H_2_***Ar, 4H), 4.57 (d, C***H_2_***OAr, *J* = 4, 2H); ^13^C NMR (125 MHz, CDCl_3_) δC: 170.1 (***C***=O), 153.2 (***C***OCH_2_), 147.2 (***C***OCH_2_CO), 141.3 (Ar***C***), 137.9 (Ar***C***), 134.9 (Ar***C***), 132.6 (***C***H = CH_2_), 129.6 (Ar***C***), 128.3 (Ar***C***), 125.2 (***C***COOH), 123.0 (Ar***C***), 120.9 (Ar***C***), 118.0 (***C***H_2_ = CH), 117.2 (Ar***C***), 112.6 (Ar***C***), 69.5 (***C***H_2_OAr), 64.6.

#### 4.2.22 Compound 22 – *tert*-butyl 3-((2-allyloxy)-5-(methyoxycarbonyl)phenoxy)methyl)-1*H*-indole-1-carboxylate

**Figure:**
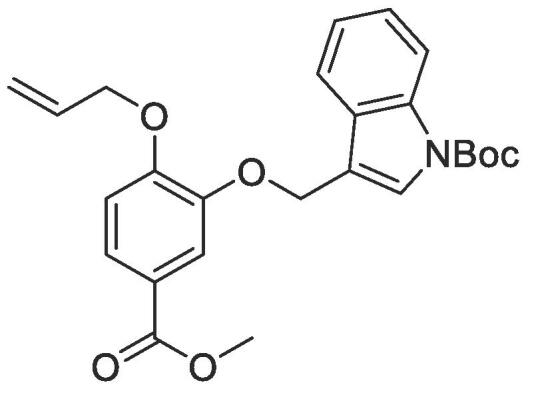

Methyl 4-(allyloxy)-3-hydroxybenzoate (0.104 g, 0.500 mmol), *tert*-butyl 3-(bromomethyl)-1*H*-indole-1-carboxylate (0.775 g, 2.50 mmol), and K_2_CO_3_ (0.553 g, 0.400 mmol) were added to anhydrous DMF (2.5 mL) and stirred at r.t. for 6 h. The reaction mixture was diluted with ethyl acetate (100 mL) and the combined organic layers washed with H_2_O (3 × 100 mL) and brine (3 × 100 mL), dried over MgSO_4_, filtered and concentrated *in vacuo*. The crude mixture was purified by column chromatography (1:5 EtOAc:*n*-Pentane) affording the title compound as a red-brown oil (0.162 g, 0.370 mmol, 74%); *R_f_* = 0.70 (1:5 EtOAc:*n*-Pentane); ^1^H NMR (400 MHz, CDCl_3_) δ_H_: 8.08–8.01 (m, Ar***H***, 4H), 7.71–7.66 (m, Ar***H****,* 1H), 7.62–7.58 (m, Ar***H****,* 2H), 7.75 (d, Ar***H****, J* = 8, 1H), 6.42–6.35 (m, C***H*** = CH_2_, 1H), 5.75 (dq, C***H_2_*** = CH, *J* = 16, 4, 1H), 5.65 (s, ArOC***H_2_***C = CH, 2H), 5.62 (dq, C***H_2_*** = CH, *J* = 8, 4, 1H), 4.98 (d, C***H_2_***OAr, *J* = 8, 2H), 4.23 (s, COOC***H_3_***, 3H), 2.02 (s, (C***H_3_***)_3_CO, 9H); ^13^C NMR (125 MHz, CDCl_3_) δC: 166.8 (***C***=O), 153.0 (***C***OCH_2_), 149.6 (***C***OCH_2_), 148.0 (***C***OCH_2_), 135.7 (***C***N), 132.7 (***C***H = CH_2_), 129.4 (***C***CCH_2_), 124.9 (***C***HN), 124.6 (Ar***C***), 124.2 (Ar***C***), 122.7 (***C***COOCH_3_), 122.7 (Ar***C***), 119.7 (Ar***C***), 117.9 (***C***H_2_ = CH), 116.4 (Ar***C***), 115.6 (Ar***C***), 115.3 (Ar***C***), 112.7 (Ar***C***), 83.8 (***C***(CH_3_)_3_), 69.6 (***C***H_2_OAr), 64.0 (***C***H_2_C), 52.0 (***C***H_3_COO), 28.1 (C(***C***H_3_)_3_); *ν*_max_/cm^−1^ (solid state) = 1720 (CO, s), 1437 (C=C), 1266 (C—O); ESI-HRMS found *m*/*z* 460.1704 [M + Na]^+^, C_25_H_27_NO_6_Na requires 460.1800.

#### 4.2.23 Compound 23 – Methyl (*S*)-4-(allyloxy)-3-(2-methoxymethoxy)propoxy)benzoate

**Figure:**
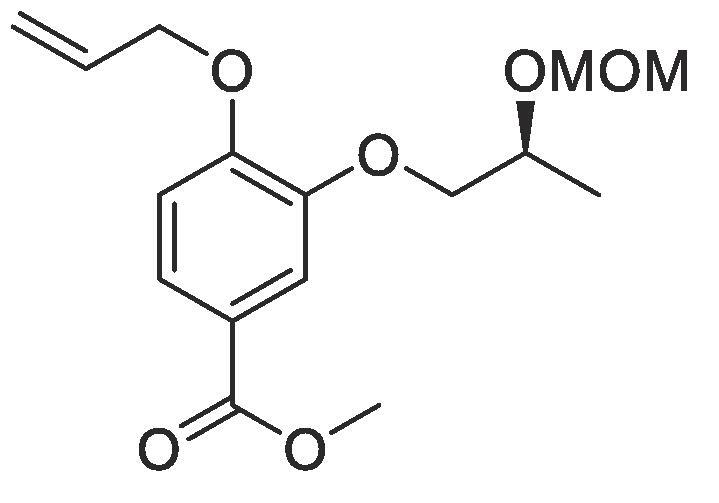

DIAD (0.491 mL, 2.50 mmol) and PPh_3_ (0.656 g, 2.50 mmol) were added to THF (2.5 mL) at 0 °C and stirred for 5 min, before addition of (*S*)-2-(methoxymethoxy)propan-1-ol (0.300 mL g, 2.50 mmol) followed and further stirring occurred for 10 min. Methyl 4-(allyloxy)-3-hydroxybenzoate (0.104 g, 0.5 mmol) in THF (0.75 mL) was added and the mixture stirred at 0 °C for 0.5 h before warming to r.t. and stirring for a further 6 h. The reaction mixture was concentrated *in vacuo* and the crude product purified by column chromatography (1:5 EtOAc:*n*-Pentane) affording the title compound as a light yellow oil (0.132 g, 0.425 mmol, 85%); *R_f_* = 0.40 (1:5 EtOAc:*n*-Pentane); ^1^H NMR (400 MHz, CDCl_3_) δ_H_: 7.63 (dd, Ar***H***, *J* = 12, 4, 1H), 7.54 (d, Ar***H***, *J* = 4, 1H), 6.86 (d, Ar***H***, *J* = 8, 1H), 6.07–5.99 (m, C***H*** = CH_2_, 1H), 5.42 (dd, C***H_2_*** = CH, *J* = 16, 4, 1H), 5.28 (dd, C***H_2_*** = CH, *J* = 8, 4, 1H), 4.77 (dd, C***H_2_***OCH_3_, *J* = 20, 8, 2H), 4.61 (dt, C***H_2_***OAr, *J* = 8, 4, 2H), 4.19–4.15 (m, C***H***CH_3_, 1H), 4.04–3.99 (m, C***H_2_***CH, 2H), 3.87 (s, C***H_3_***COO, 3H), 3.41 (s, C***H_3_***O, 3H), 1.31 (d, C***H_3_***CH, 3H); ^13^C NMR (125 MHz, CDCl3) δ_C_: 166.8 (***C***=O), 152.6 (***C***OCH_2_), 148.4 (***C***OCH_2_CH), 132.7 (***C***H = CH_2_), 123.8 (***C***COOCH_3_), 122.9 (Ar***C***), 117.9 (C***H_2_*** = CH), 114.4 (Ar***C***), 112.5 (Ar***C***), 95.4 (***C***H_2_O), 73.1 (***C***H_2_CH), 71.1 (***C***HCH_3_), 69.5 (***C***H_2_OAr), 55.3 (***C***H_3_O), 51.9 (***C***H_3_COO), 17.4 (***C***H_3_CH); *ν*_max_/cm^−1^ (solid state) = 1720 (CO, s), 1468 (C=C), 1213 (C—O); ESI-HRMS found *m*/*z* 311.1491 [M + H]+, C_16_H_23_O_6_ requires 311.1495.

#### 4.2.24 Compound 24 – Methyl 4-hydroxy-isopropoxybenzoate

**Figure:**
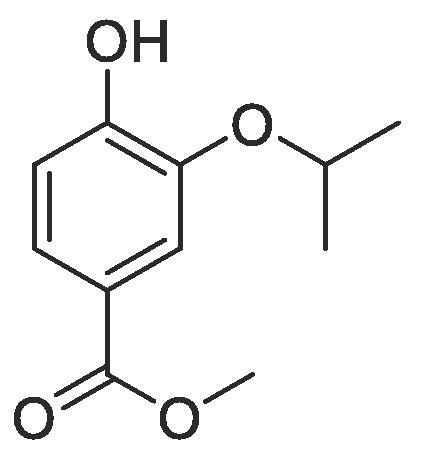

To a solution of methyl 4-(allyloxy)-3-isopropoxybenzoate (0.570 g, 2.28 mmol) in MeOH (20 mL) was added Pd(PPh_3_)_4_ (0.026 g, 0.023 mmol) and the reaction mixture stirred for 0.25 h at r.t. K_2_CO_3_ (0.945 g, 6.84 mmol) was then added and the reaction mixture stirred at r.t. for 4 h. The reaction mixture was concentrated *in vacuo* and acidified to pH 4–5 with 2 M HCl. The product was extracted with DCM (2 × 50 mL) and the combined organic layers washed with brine (2 × 50 mL), dried over MgSO_4_, filtered and concentrated *in vacuo* affording the title compound as a colourless oil (0.321 g, 1.54 mmol, 67%); *R_f_* = 0.40 (1:5 EtOAc:*n*-Pentane); ^1^H NMR (400 MHz, CDCl_3_) δ_H_: 7.61 (dd, Ar***H***, *J* = 8, 4, 1H), 7.55 (d, Ar***H***, *J* = 4, 1H), 6.94 (d, Ar***H***, *J* = 12, 1H), 4.68 (quint, C***H***(CH_3_)_2_, *J* = 4, 1H), 3.87 (s, COOC***H_3_***, 3H), 1.39 (d, CH(C***H_3_***)_2_, *J* = 4, 6H); ^13^C NMR (125 MHz, CDCl_3_) δ_C_: 167.1 (***C***=O), 151.1 (***C***OH), 144.4 (***C***OCH), 128.7 (***C***COOH), 124.2 (Ar***C***), 122.3 (Ar***C***), 114.3 (Ar***C***), 72.1 (***C***H(CH_3_)_2_), 52.1 (***C***H_3_COO), 22.2 ((***C***H_3_)_2_CH); *ν*_max_/cm^−1^ (solid state) = 3348 (OH, br), 1690 (CO, s), 1249 (C—O); ESI-HRMS found *m*/*z* 210.00 [M + H]^+^, C_11_H_14_O_4_ requires 210.09.

#### 4.2.25 Compound 25 – 2-isopropoxy-4-(methoxycarbonyl)phenyl 4-(allyloxy)-3-isopropoxybenzoate

**Figure:**
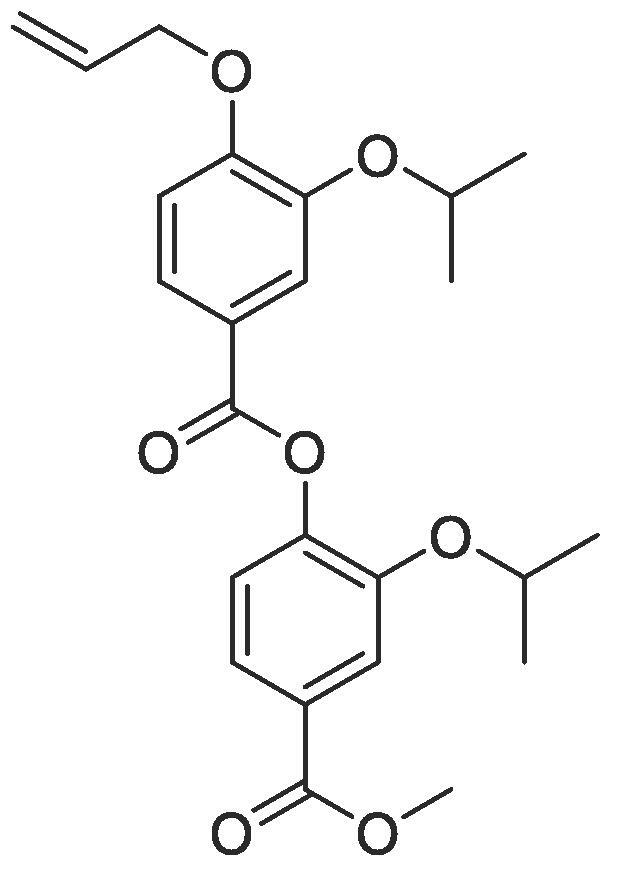

To a mixture of EDC.HCl (0.288 g, 1.50 mmol), HOBt (0.203 g, 1.50 mmol), and DIPEA (0.523 mL, 3.00 mmol) in anhydrous DMF (5 mL) was added 4-(allyloxy)-3-isopropoxybenzoic acid (177 mg, 0.750 mmol). After stirring for 5 min at r.t., methyl 4-hydroxy-isopropoxybenzoate was added (105 mg, 0.5 mmol). The reaction was stirred at r.t. for 5 h, and, following completion, the crude product was extracted using ethyl acetate (3 × 10 mL). The combined organic layers were washed with LiCl solution (3 × 15 mL), Na_2_CO_3_ (3 × 15 mL), and brine (3 × 15 mL), and the resulting solution concentrated *in vacuo*. Purification was performed using column chromatography (1:9 EtOAc:*n*-Pentane) to isolate the title compound as a colourless oil (0.196 g, 0.460 mmol, 92%); *R_f_* = 0.50 (1:5 EtOAc:*n*-Pentane); ^1^H NMR (400 MHz, CDCl_3_) δ_H_: 7.81 (dd, Ar***H***, J = 8, 4, 1H), 7.72 (d, Ar***H***, J = 4, 1H), 7.70–7.67 (m, Ar***H***, 2H), 7.22 (d, Ar***H***, J = 12, 1H), 6.96 (d, Ar***H***, J = 12, 1H), 6.12–6.05 (m, C***H*** = CH_2_, 1H), 5.46 (dq, C***H_2_*** = CH, *J* = 16, 4, 1H), 5.32 (dq, C***H_2_*** = CH, *J* = 8, 4, 1H), 4.69 (dt, C***H_2_***OAr, J = 8, 4, 2H), 4.63–4.58 (m, C***H***(CH_3_)_2_, 2H), 3.92 (s, C***H_3_***COO, 3H), 1.39 (d CH(C***H_3_***)_2_, J = 8, 6H), 1.28 (d, CH(C***H_3_***)_2_, J = 8, 6H); ^13^C NMR (125 MHz, CDCl_3_) δ_C_: 166.7 (***C***=O), 164.2 (***C***=O), 154.4 (***C***OCH), 149.9 (***C***OCH_2_), 147.6 (***C***OCH), 145.6 (***C***OCO), 133.0 (***C***H = CH_2_), 128.6 (***C***COOCH_3_), 124.9 (Ar***C***), 123.3 (***C***COOCAr), 122.7 (Ar***C***) 122.0 (Ar***C***), 118.3 (***C***H_2_ = CH), 116.8 (Ar***C***), 113.2 (Ar***C***), 72.3 (***C***H(CH_3_)_2_), 72.1 (***C***H(CH_3_)_2_), 69.9 (***C***H_2_OAr), 52.4 (***C***H_3_COO), 22.2 (C(***C***H_3_)_2_); *ν*_max_/cm^−1^ (solid state) = 1721 (CO, s), 1481 (C=C), 1261 (C—O); ESI-HRMS found *m*/*z* 427.1760 [M−H]^-^, C_24_H_27_O_7_ requires 427.1757.

#### 4.2.26 Compound 26 – Methyl 4-(allyloxy)-3-methoxybenzoate

**Figure:**
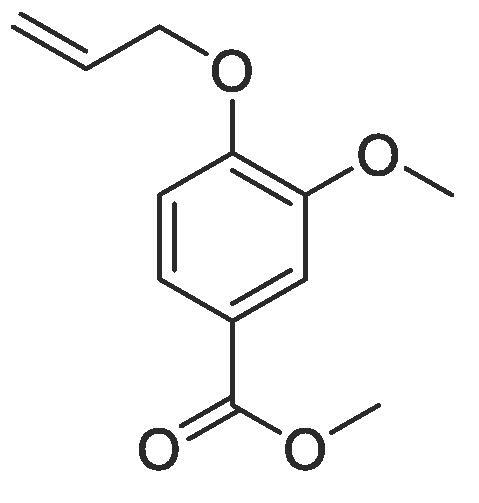

Methyl 4-hydroxy-3-methyoxybenzoate (0.5 g, 2.75 mmol), allyl bromide (0.24 mL, 2.75 mmol), and K_2_CO_3_ (0.45 g, 3.3 mmol) were added to anhydrous DMF (10 mL) and stirred at r.t. for 3 h. The resulting mixture was extracted using ethyl acetate (2 × 20 mL) and the combined organic layers washed with H_2_O (3 × 50 mL) and brine (3 × 50 mL), dried over MgSO_4_, filtered and concentrated *in vacuo* affording the title compound as a white crystalline solid (0.51 g, 2.3 mmol, 84%); ^1^H NMR (400 MHz, CDCl_3_) δ_H_: 7.58 (dd, Ar***H***, *J* = 8, 4, 1H), 7.49 (d, Ar***H***, *J* = 4, 1H), 6.83 (d, Ar***H***, *J* = 8, 1H), 6.06–5.97 (m, C***H*** = CH_2_, 1H), 5.37 (dq, C***H_2_*** = CH, *J* = 16, 4, 1H), 5.26 (dq, C***H_2_*** = CH, *J* = 8, 4, 1H), 4.61 (dt, C***H_2_***OAr, *J* = 8, 4, 2H), 3.87 (s, COOC***H_3_***, 3H), 3.83 (s, C***H_3_***, 3H); ^13^C NMR (125 MHz, CDCl_3_) δ_C_: 166.8 (***C***=O), 151.9 (***C***OCH_2_), 148.8 (***C***OCH_3_), 132.5 (***C***H = CH_2_), 123.3 (Ar***C***), 122.8 (***C***COOCH_3_), 118.4 (***C***H_2_ = CH_2_), 112.2 (Ar***C***), 111.9 (Ar***C***), 69.6 (***C***H_2_OAr), 56.0 (***C***H_3_), 51.9 (***C***H_3_COO).

#### 4.2.27 Compound 27–4-(allyloxy)-3-methoxybenzoic acid

**Figure:**
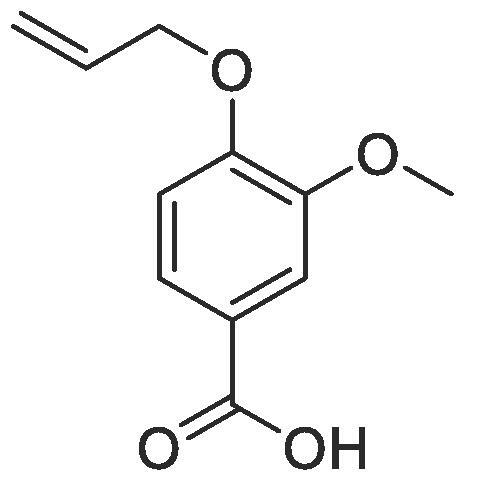

Methyl 4-(allyloxy)-3-methoxybenzoate (0.51 g, 2.3 mmol) was added to 2 M NaOH (2 mL, 14 mmol) and MeOH (15 mL) and the reaction mixture stirred at 50 °C for 1 h. The reaction was acidified to pH 2–3 by drop-wise addition of 2 M HCl. The product was extracted with ethyl acetate (2 × 50 mL) and the combined organic layers were dried over MgSO_4_, filtered and concentrated *in vacuo* affording the title compound as a yellow solid (0.48 g, 2.3 mmol, 99%); ^1^H NMR (400 MHz, CDCl_3_) δ_H_: 7.73 (dd, Ar***H***, *J* = 8, 4, 1H), 7.59 (d, Ar***H***, *J* = 4, 1H), 6.91 (d, Ar***H***, *J* = 8, 1H), 6.14–6.04 (m, C***H*** = CH_2_, 1H), 5.44 (dq, C***H_2_*** = CH, *J* = 16, 4, 1H), 5.34 (dq, C***H_2_*** = CH, *J* = 8, 4, 1H), 4.69 (dt, C***H_2_***OAr, *J* = 8, 4, 2H), 3.94 (s, C***H_3_***, 3H); ^13^C NMR (125 MHz, CDCl_3_) δ_C_: 172.0 (***C***=O), 152.8 (***C***OCH_2_), 149.0 (***C***OCH_3_), 132.5 (***C***H = CH_2_), 124.5 (Ar***C***), 122.1 (***C***COOCH_3_), 118.8 (***C***H_2_ = CH_2_), 112.8 (Ar***C***), 112.0 (Ar***C***), 69.9 (***C***H_2_OAr).

#### 4.2.28 Compound 28 – 2-methyoxy-4-(methoxycarbonyl)phenyl 4-(allyloxy)-3-methoxybenzoate

**Figure:**
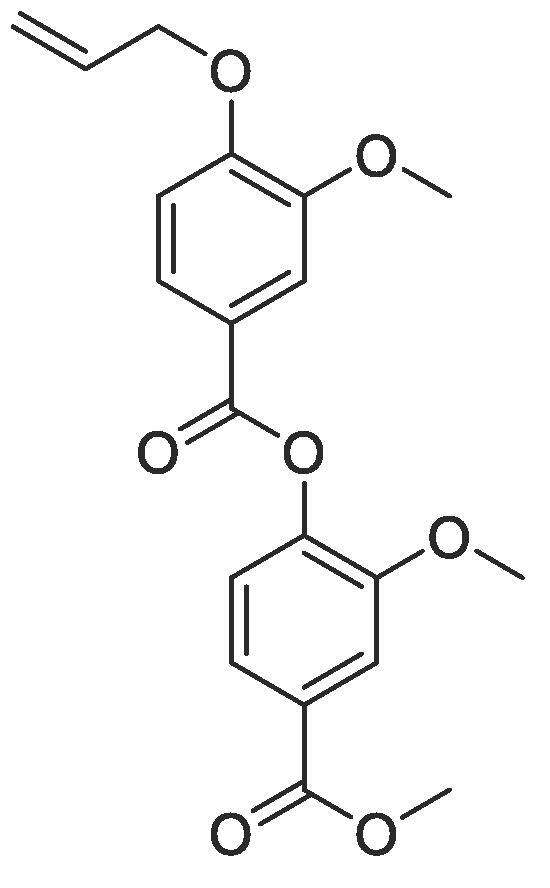

To a mixture of EDC.HCl (0.77 g, 4 mmol), Oxyma (0.57 g, 4 mmol), and DIPEA (1.4 mL, 8 mmol) in anhydrous DMF (15 mL) was added 4-(allyloxy)-3-methoxybenzoic acid (420 mg, 2 mmol). After stirring for 5 min at r.t., methyl 4-hydroxy-3-methoxybenzoate was added (245 mg, 1.4 mmol). The reaction was stirred at r.t. for 5 h, and, following completion, the crude product was extracted using ethyl acetate (3 × 30 mL). The combined organic layers were washed with LiCl solution (3 × 20 mL), Na_2_CO_3_ (3 × 20 mL), and brine (3 × 20 mL), and the resulting solution concentrated *in vacuo*. Purification was performed using column chromatography (1:1 EtOAc:Heptane) to isolate the title compound as a pale yellow solid (0.306 g, 0.82 mmol, 61%); ^1^H NMR (400 MHz, CDCl_3_) δ_H_: 7.84 (dd, Ar***H***, J = 8, 4, 1H), 7.73–7.68 (m, Ar***H***, 3H), 7.21 (d, Ar***H***, J = 8, 1H), 6.95 (d, Ar***H***, J = 8, 1H), 6.13–6.07 (m, C***H*** = CH_2_, 1H), 5.45 (dq, C***H_2_*** = CH, *J* = 16, 4, 1H), 5.35 (dq, C***H_2_*** = CH, *J* = 8, 4, 1H), 4.69 (d, C***H_2_***OAr, J = 4, 2H), 3.96 (s, C***H_3_***COO, 3H), 3.94 (s, C***H_3_***, 3H), 3.88 (s, C***H_3_***, 3H); ^13^C NMR (125 MHz, CDCl_3_) δ_C_: 166.6 (***C***=O), 164.2 (***C***=O), 152.8 (***C***OH), 151.5 (***C***OCH_3_), 149.2 (***C***OCH_3_), 144.2 (***C***OCO), 132.5 (***C***H = CH_2_), 128.9 (***C***COO), 124.6 (***C***COO), 123.1 (Ar***C***), 122.8 (Ar***C***) 121.2 (Ar***C***), 118.9 (***C***H_2_ = CH_2_), 113.6 (Ar***C***), 112.9 (Ar***C***), 112.1 (Ar***C***), 69.9 (***C***H_2_OAr), 56.3 (***C***H_3_), 56.3 (***C***H_3_), 52.5 (***C***H_3_COO).

#### 4.2.29 Compound 29 – 2-methyoxy-4-(methoxycarbonyl)phenyl 4-hydroxy-3-methoxybenzoate

**Figure:**
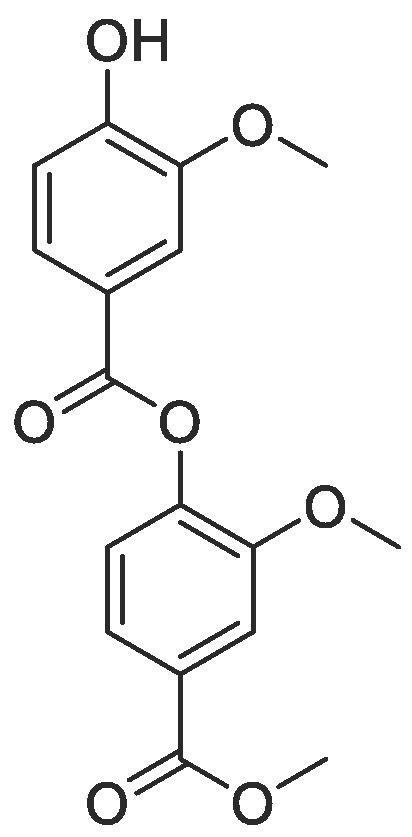

To a solution of 2-methyoxy-4-(methoxycarbonyl)phenyl 4-(allyloxy)-3-methoxybenzoate (0.120 g, 0.32 mmol) in THF (5 mL) was added Pd(PPh_3_)_4_ (7.5 mg, 6.4 μmol) and the reaction mixture stirred for 5 min at r.t. NaBH_4_ (0.024 g, 0.64 mmol) was then added and the reaction mixture stirred at r.t. for 2 h. The reaction mixture was concentrated *in vacuo* and acidified to pH 4–5 with 1 M HCl. The product was extracted with DCM (2 × 10 mL) and the combined organic layers washed with brine (2 × 10 mL), dried over MgSO_4_, filtered and concentrated *in vacuo* affording the title compound as a colourless oil (0.47 g, 0.14 mmol, 44%); ^1^H NMR (400 MHz, CDCl_3_) δ_H_: 7.84 (dd, Ar***H***, J = 8, 4, 1H), 7.73–7.67 (m, Ar***H***, 3H), 7.21 (d, Ar***H***, J = 8, 1H), 7.01 (d, Ar***H***, J = 8, 1H), 3.98 (s, C***H_3_***COO, 3H), 3.94 (s, C***H_3_***, 3H), 3.88 (s, C***H_3_***, 3H); ^13^C NMR (125 MHz, CDCl_3_) δ_C_: 166.6 (***C***=O), 164.2 (***C***=O), 151.5 (***C***OH), 150.9 (***C***OCH_3_), 146.4 (***C***OCH_3_), 144.2 (***C***OCO), 128.9 (***C***COO), 125.4 (***C***COO), 123.1 (Ar***C***), 122.8 (Ar***C***) 121.1 (Ar***C***), 114.4 (Ar***C***), 113.6 (Ar***C***), 112.4 (Ar***C***), 56.3 (***C***H_3_), 56.3 (***C***H_3_), 52.5 (***C***H_3_COO).

### 4.3 Conformational analysis

#### 4.3.1 X-ray crystallography

*Crystal data for***28**: C_20_H_20_O_7_, *M* = 372.36, monoclinic, *P*2_1_/*n* (no. 14), *a* = 11.9797(6), *b* = 10.4128(4), *c* = 14.3753(7) Å, β = 94.453(4)°, *V* = 1787.79(14) Å^3^, *Z* = 4, *D*_c_ = 1.383 g cm^−3^, μ(Mo-Kα) = 0.105 mm^−1^, *T* = 173 K, colourless blocks, Agilent Xcalibur 3 E diffractometer; 3765 independent measured reflections (*R*_int_ = 0.0289), *Fn^2^* refinement,^1^, ^2^ *R*_1_(obs) = 0.0488, w*R*_2_(all) = 0.1254, 2574 independent observed absorption-corrected reflections [|*F*_o_| > 4σ(|*F*_o_|), completeness to θ_full_(25.2°) = 99.8%], 261 parameters. CCDC 2242268.

**Fig. 4:**
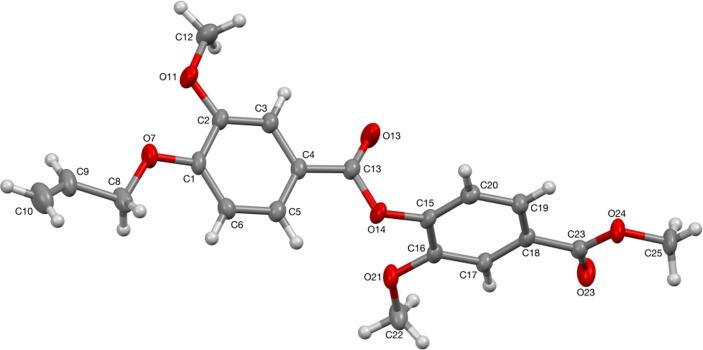
Crystal structure of **28** (50% probability of elipsoids).

The O7-bound –CH_2_–CH = CH_2_ moiety in the structure of **28** was found to be disordered. Two orientations were identified of *ca*. 88 and 12% occupancy, their geometries were optimised, the thermal parameters of adjacent atoms were restrained to be similar, and only the non-hydrogen atoms of the major occupancy orientation were refined anisotropically (those of the minor occupancy orientation were refined isotropically) (see Fig. 4).

#### 4.3.2 Molecular modelling

Superimposition of the oligoester with the Bak peptide α-helix was conducted using MOE2022.02 software. The oligoester was energy minimized using the Amber10:EHT forcefield. The central ester bond was then selected and, using the bidirectional torsion profile, conformational analysis was conducted. This gave a standard sin(θ) curve for the dihedral angle ranging from −180° to 180° across this bond.

The Bak peptide was imported into MOE using PDB ID 1BXL. The in-built ‘QuickPrep’ function was used to allow for energy minimisation and side-chain resolution. The oligoester was then superimposed, using the ‘superpose molecules’ function such that the oligoester oxygen atom of the OMe aligned with the α-carbon of the side chain in the Bak peptide. This was done at the *i*, *i + 4*, *i + 7* positions of the α-helix. In addition, the carbon atom in the central phenyl ring of the oligoester ‘backbone’ was superimposed with the corresponding backbone carbonyl of the Bak peptide to ensure the correct directionality was obtained. Using the SVL function, a RMSD value of 0.83 Å was obtained.

#### 4.3.3 Aqueous stability

To pre-prepared 100 mM pH buffer solutions (1.00 mL), including pH 2.5, 5.0, 7.4, 10.0 and 12.5 (Table 2), was added the dimer (10 mg/mL in MeCN) at r.t, forming a final solution with dimer concentration 5 mg/mL. The mixture was stirred at r.t. for 7 days and monitored via TLC and LCMS (50% MeCN) to follow ester bond degradation.

**Table 2:** Buffer salts used to generate solutions from pH 2.5 to 12.5 dissolved in 5 mL H_2_O.

**pH**	**Components**	**Quantity**
2.5	Sodium acetate Acetic acid	2 mg 29 mg
5.0	Sodium acetate Acetic acid	28 mg 10 mg
7.4	Phosphate buffered saline (PBS)	1 tablet
10.0	Sodium bicarbonate Sodium carbonate	19 mg 29 mg
12.5	Sodium bicarbonate Sodium carbonate	5 mg 46 mg

## Declaration of Competing Interest

The authors declare that they have no known competing financial interests or personal relationships that could have appeared to influence the work reported in this paper.

## Data Availability

All raw data has been uploaded onto the Imperial College Data Repository, the DOI is shared in the manuscript.
