# Supplementary material for: Aromatic oligoesters as novel helix mimetic scaffolds
Source: Bioorg Med Chem. 2023 May 3;87:117311. doi: 10.1016/j.bmc.2023.117311 (PMC10250785; doi:10.1016/j.bmc.2023.117311)
Supplement: Supplementary data 1 [file mmc1.docx]

**Aromatic Oligoesters as Novel Helix Mimetic Scaffolds**

Muhammed Haque, Theo Flack, Ravi Singh, Archie Wall, Guilherme Vieira de Castro, Lishen Jiang, Andrew J. P. White and Anna Barnard*

**Supporting Information**

_
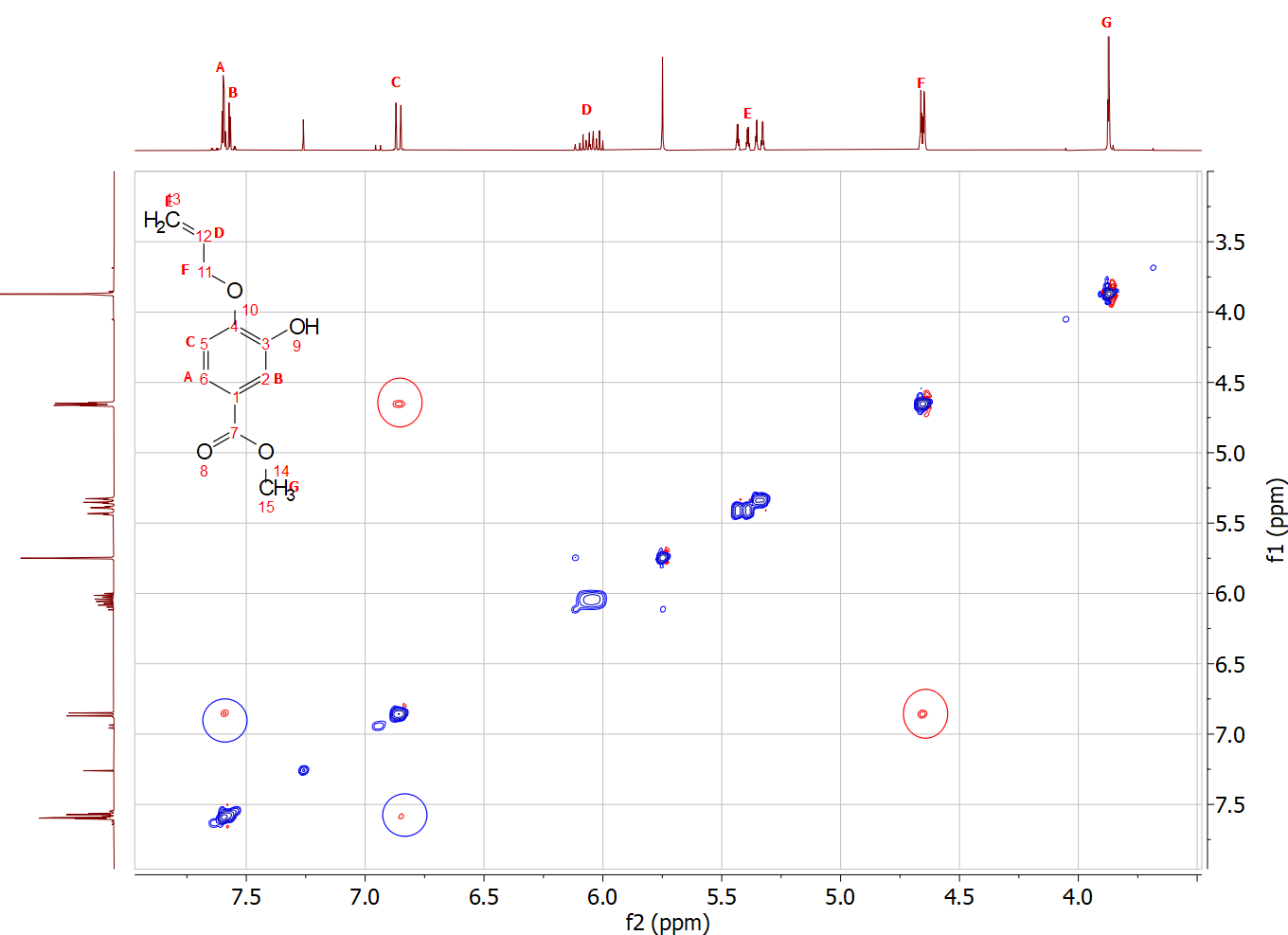
_

*Figure* S1 - NOESY Spectra of Compound **1** in CDCl_3_. Proton C was assigned through observation of coupling with proton A (blue circles) and also coupled with protons F (red circles) confirming installation of a *para*-allyl group.


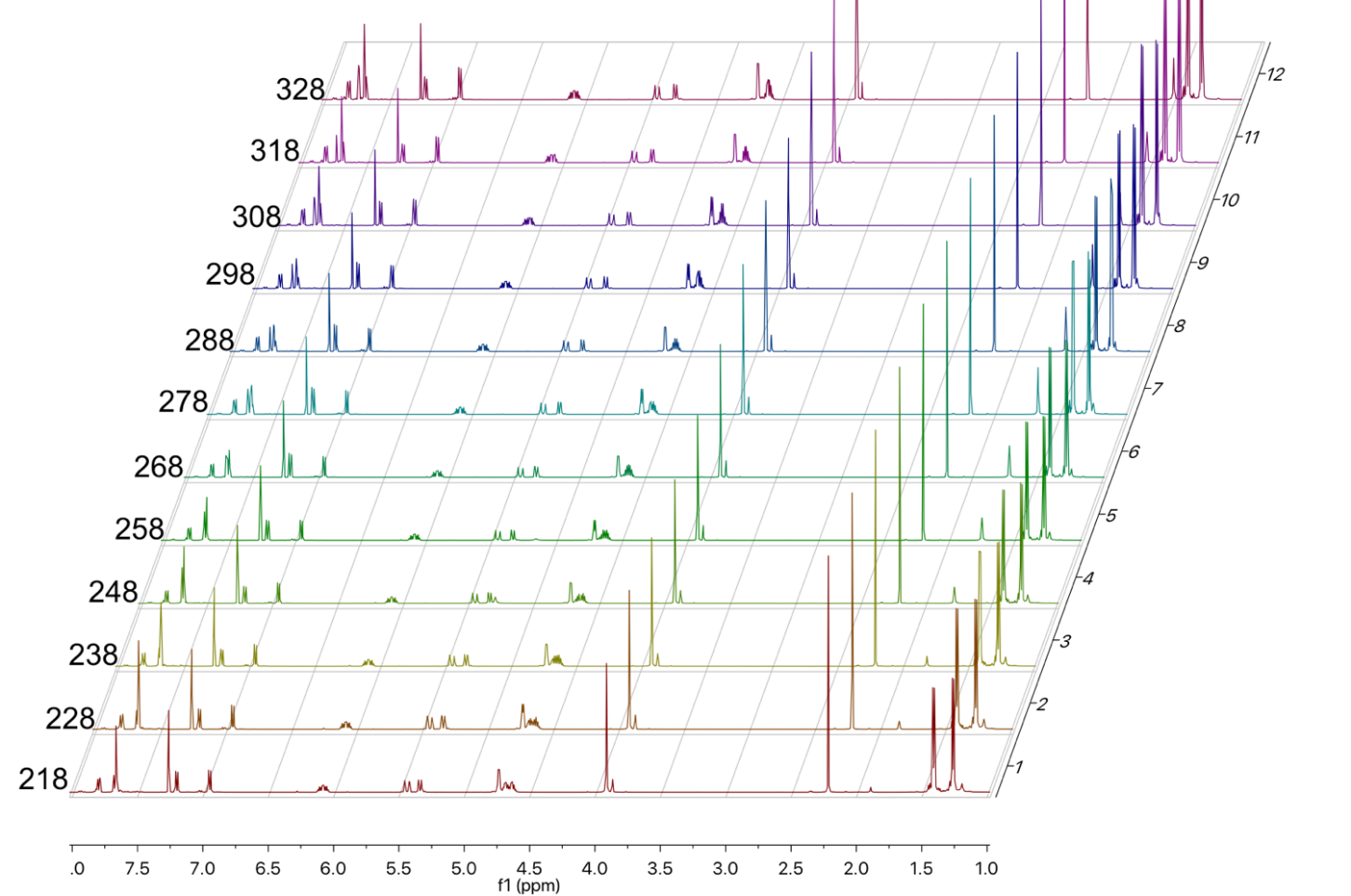


*Figure S2* – Variable temperature NMR of dimer **25** conducted at 10K intervals from 218 to 328K in CDCl_3_


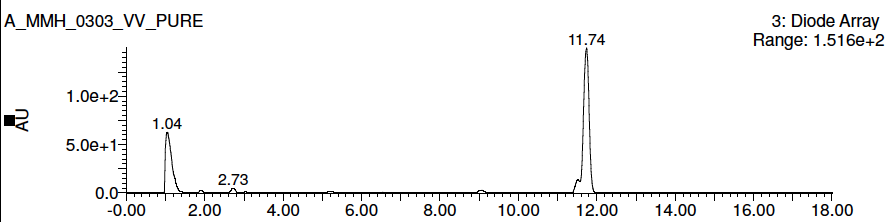

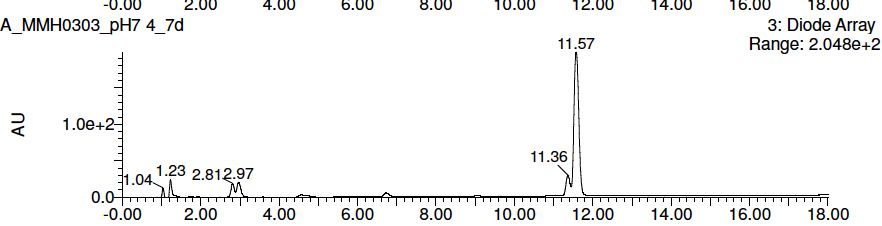

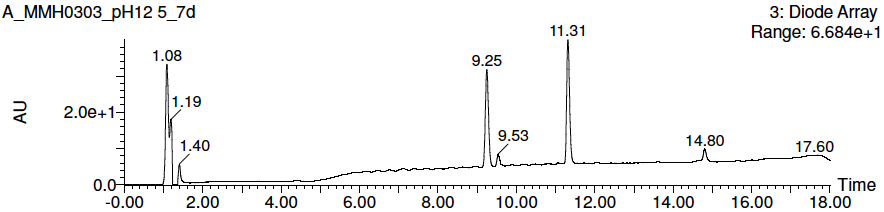


*Figure S3* – LC-MS UV Diode array traces of dimer **25** initially (top) and after 7 days at pH 7.4 (middle) and pH 12.5 (bottom).

*Table S1* – Chemical structures of monomers and dimers synthesised

| **Compound Number** | **Structure** | **Compound Number** | **Structure** |
| --- | --- | --- | --- |
| **1** |  | **2** |  |
| **3** |  | **4** |  |
| **5** |  | **6** |  |
| **7** |  | **8** |  |
| **9** |  | **10** |  |
| **11** |  | **12** |  |
| **13** |  | **14** |  |
| **15** |  | **16** |  |
| **17** |  | **18** |  |
| **19** |  | **20** |  |
| **21** |  | **22** |  |
| **23** |  | **24** |  |
| **25** |  | **26** |  |
| **27** |  | **28** |  |
| **29** |  |  |  |

**NMR Spectra**

NMR Spectra files are available online via the Imperial College London Data Repository at the following DOI: [10.14469/hpc/12265](https://doi.org/10.14469/hpc/12265) and as pdf images below.

**
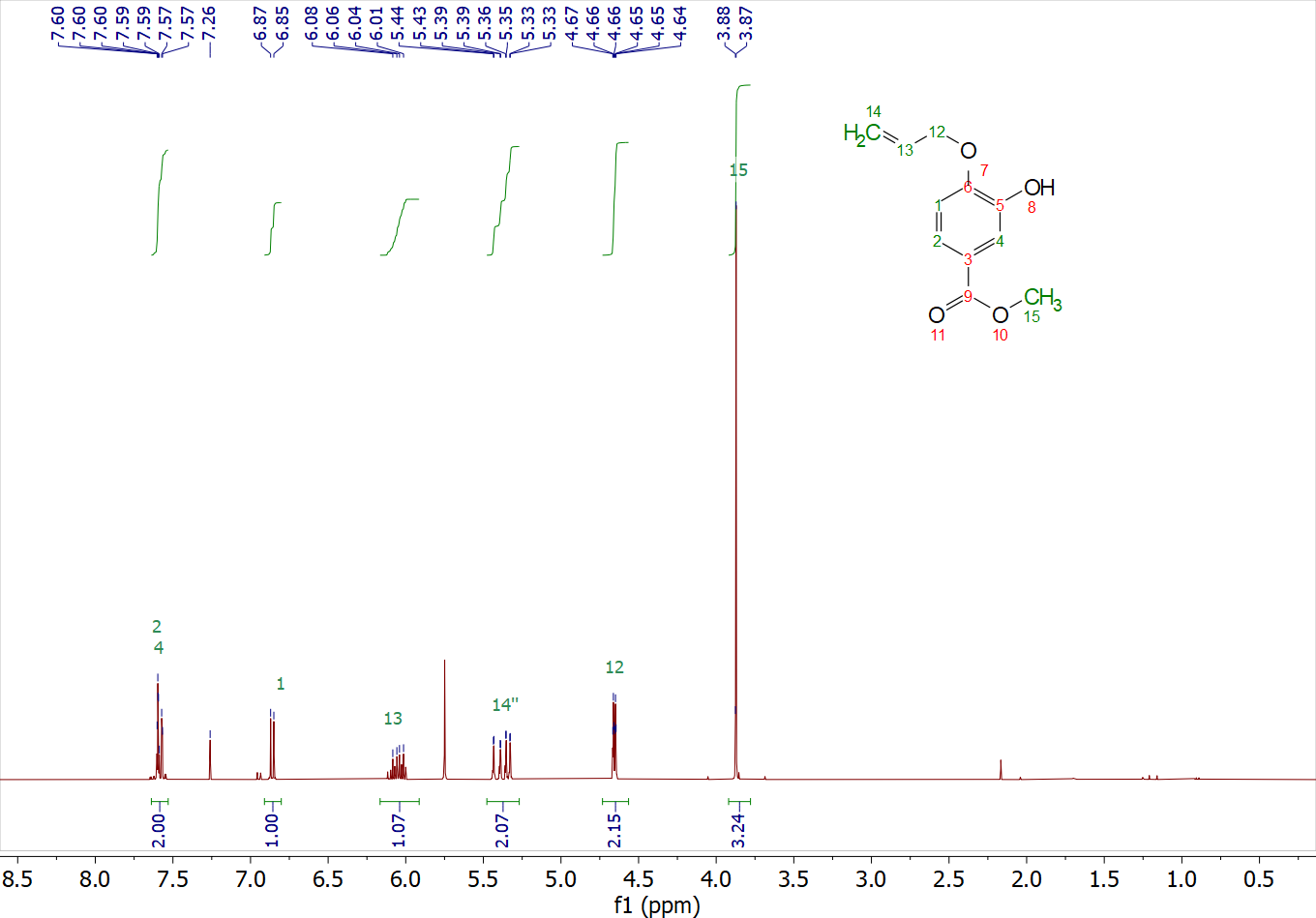
**

^1^H NMR Spectra of Compound **1** in CDCl_3_

_
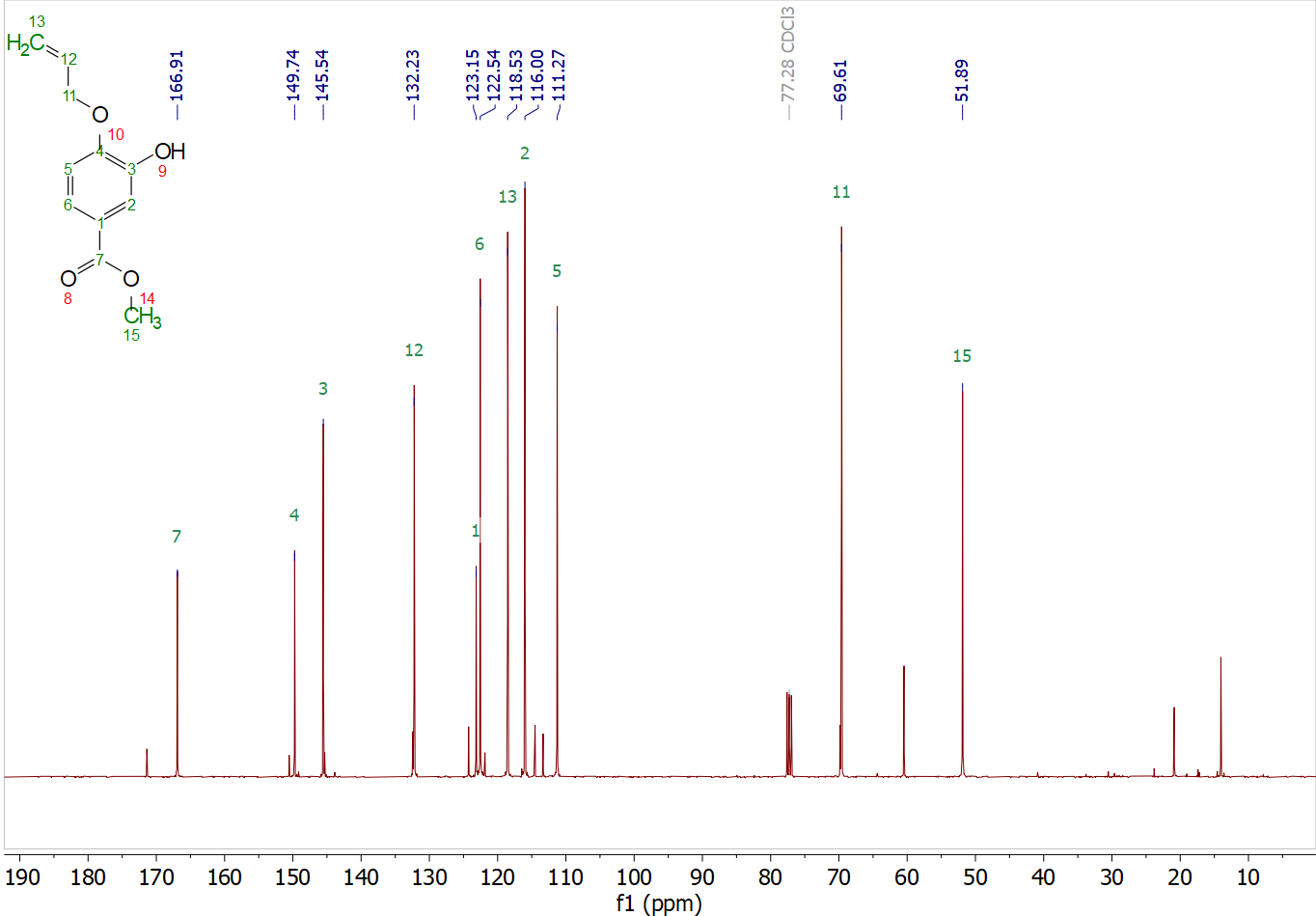
_

^13^C NMR Spectra of Compound **1** in CDCl_3_

_
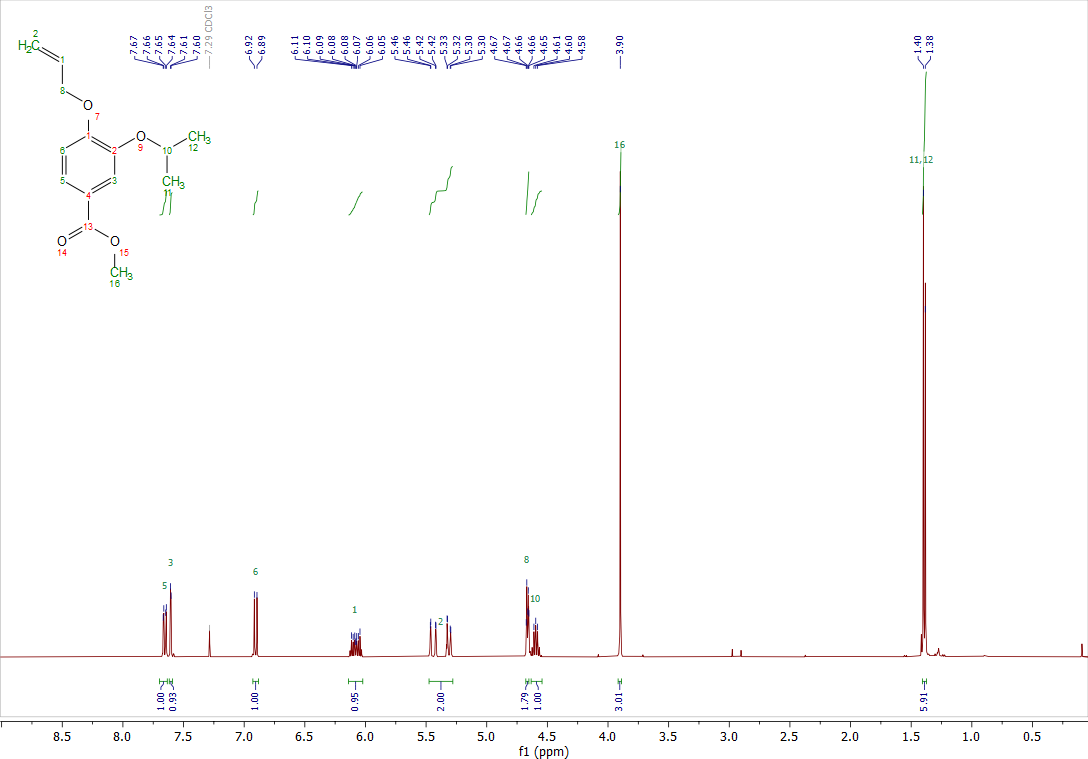
_

^1^H NMR Spectra of Compound **2** in CDCl_3_


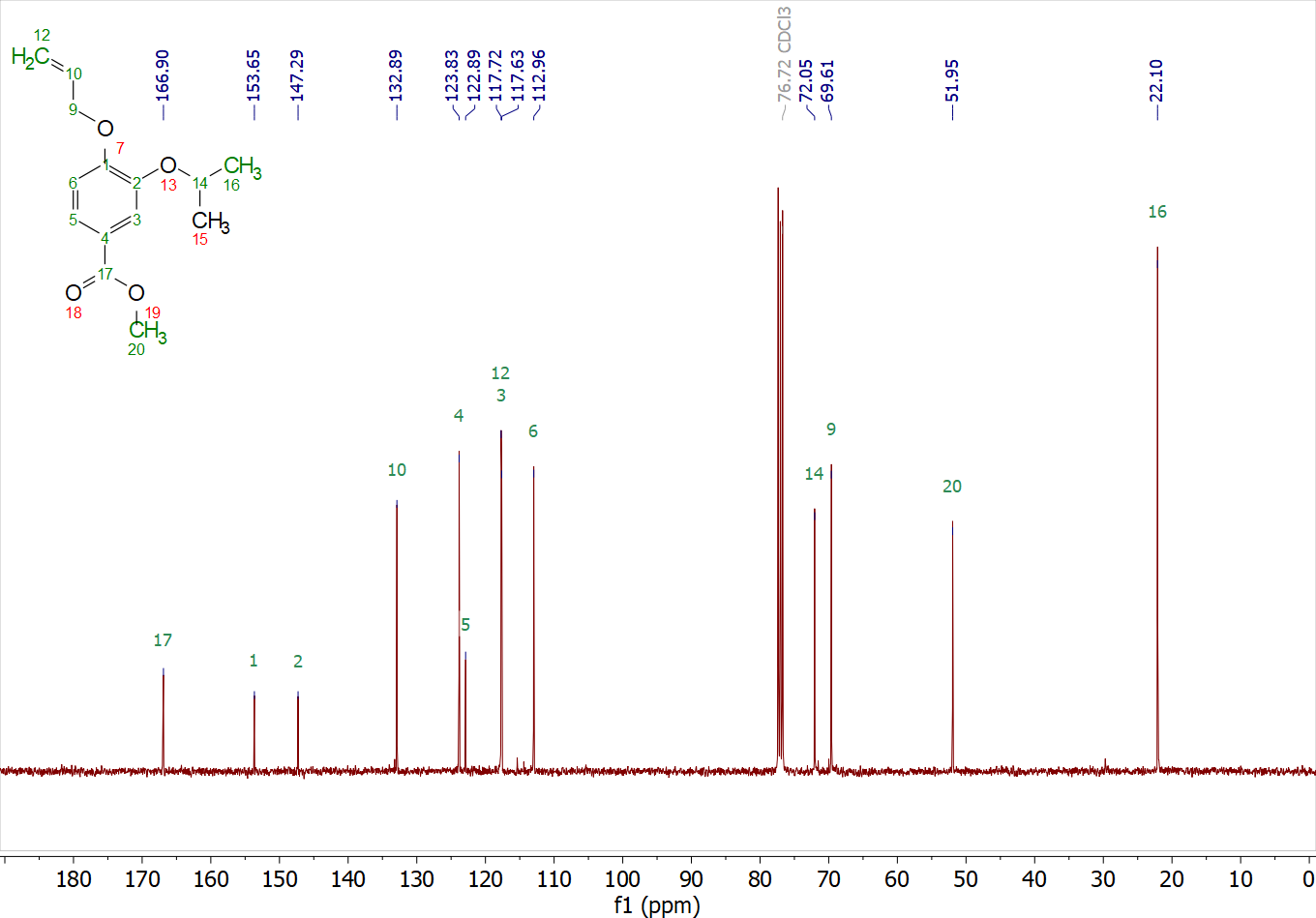


^13^C NMR Spectra of Compound **2** in CDCl_3_


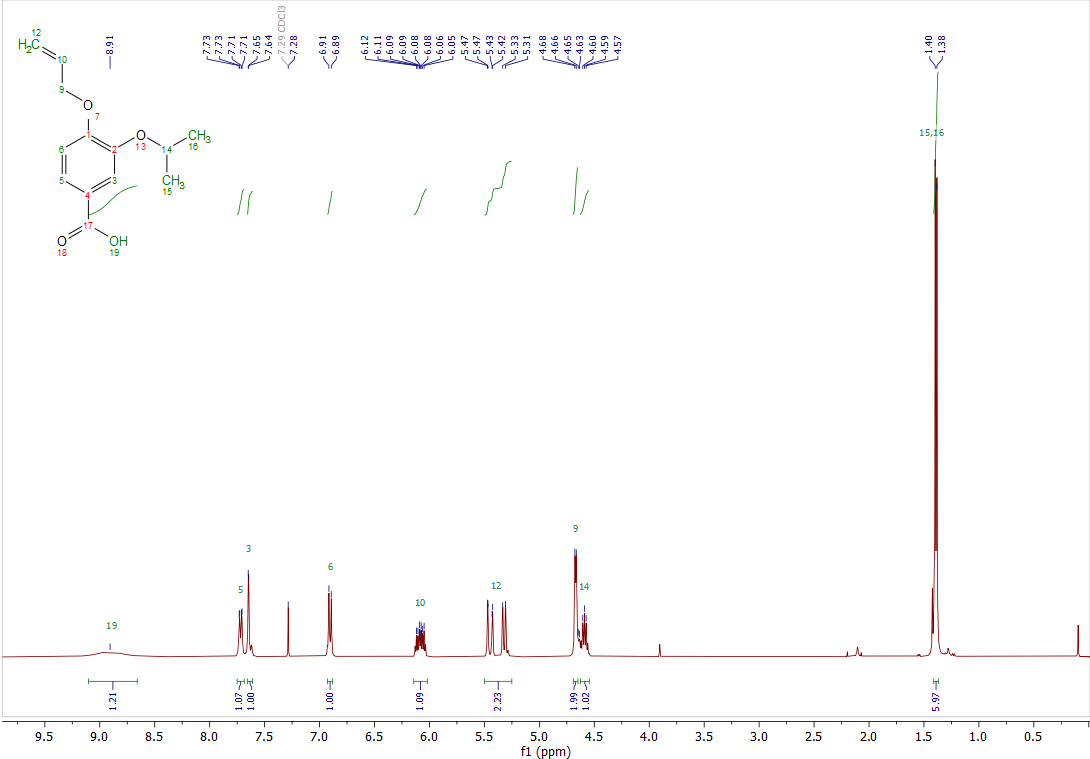


^1^H NMR Spectra of Compound **3** in CDCl_3_

_
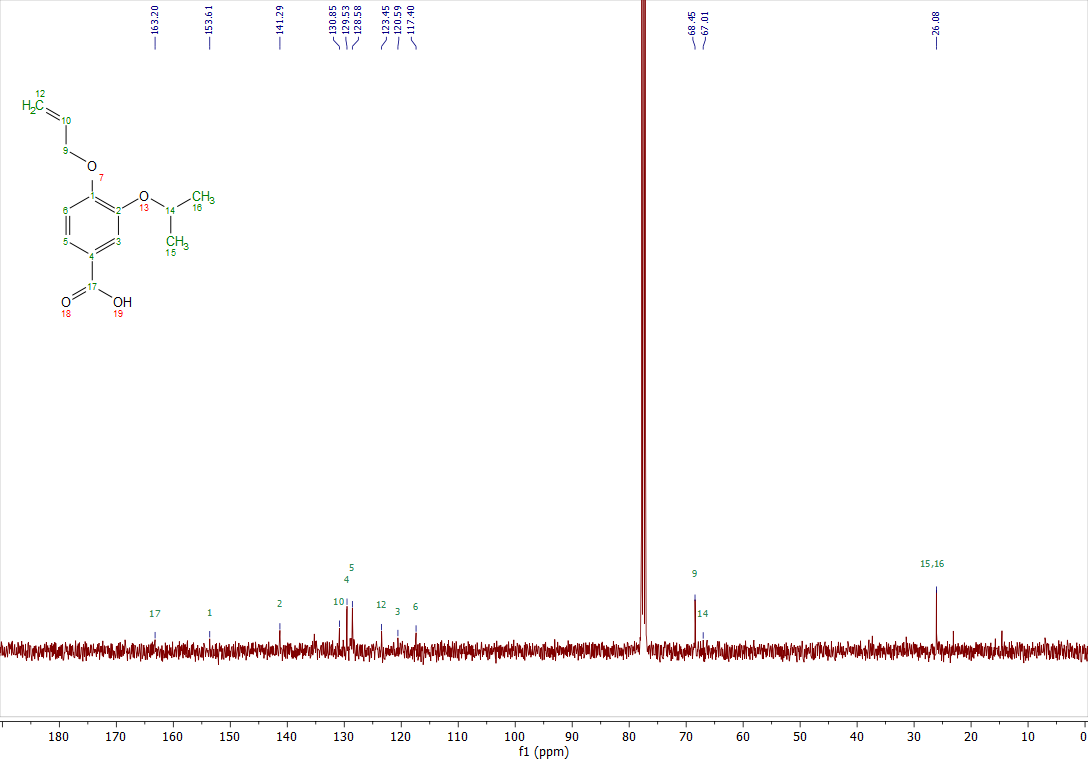
_

^13^C NMR Spectra of Compound **3** in CDCl_3_

_
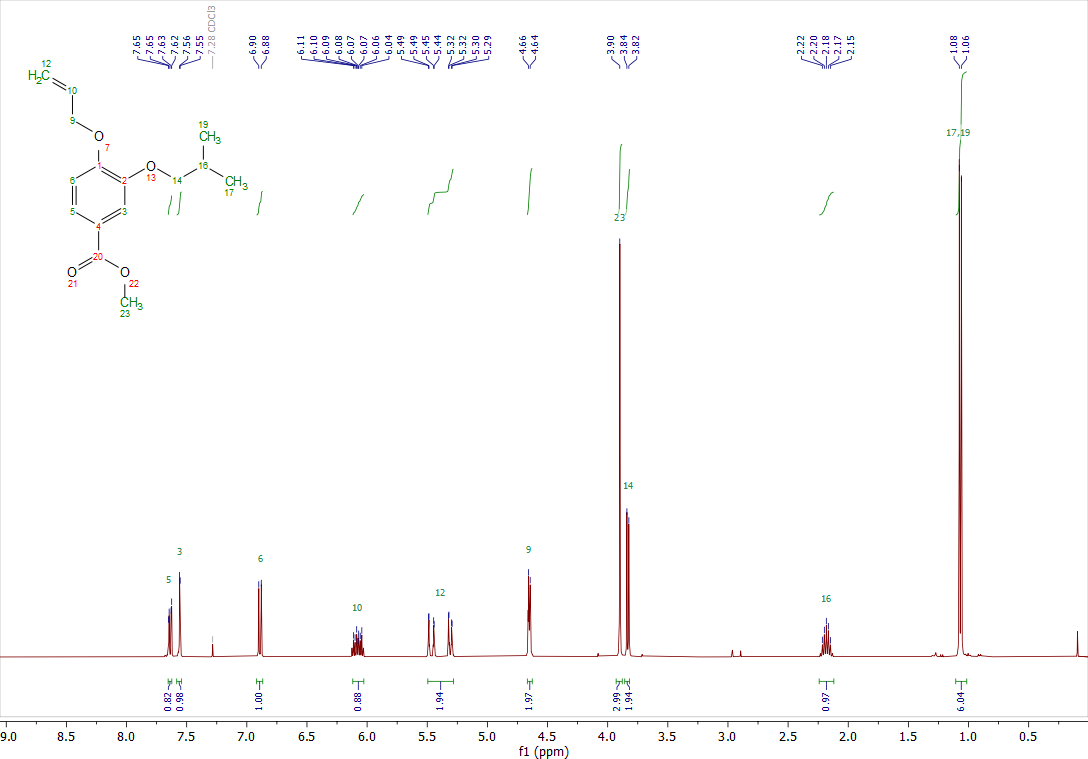
_

^1^H NMR Spectra of Compound **4** in CDCl_3_

_
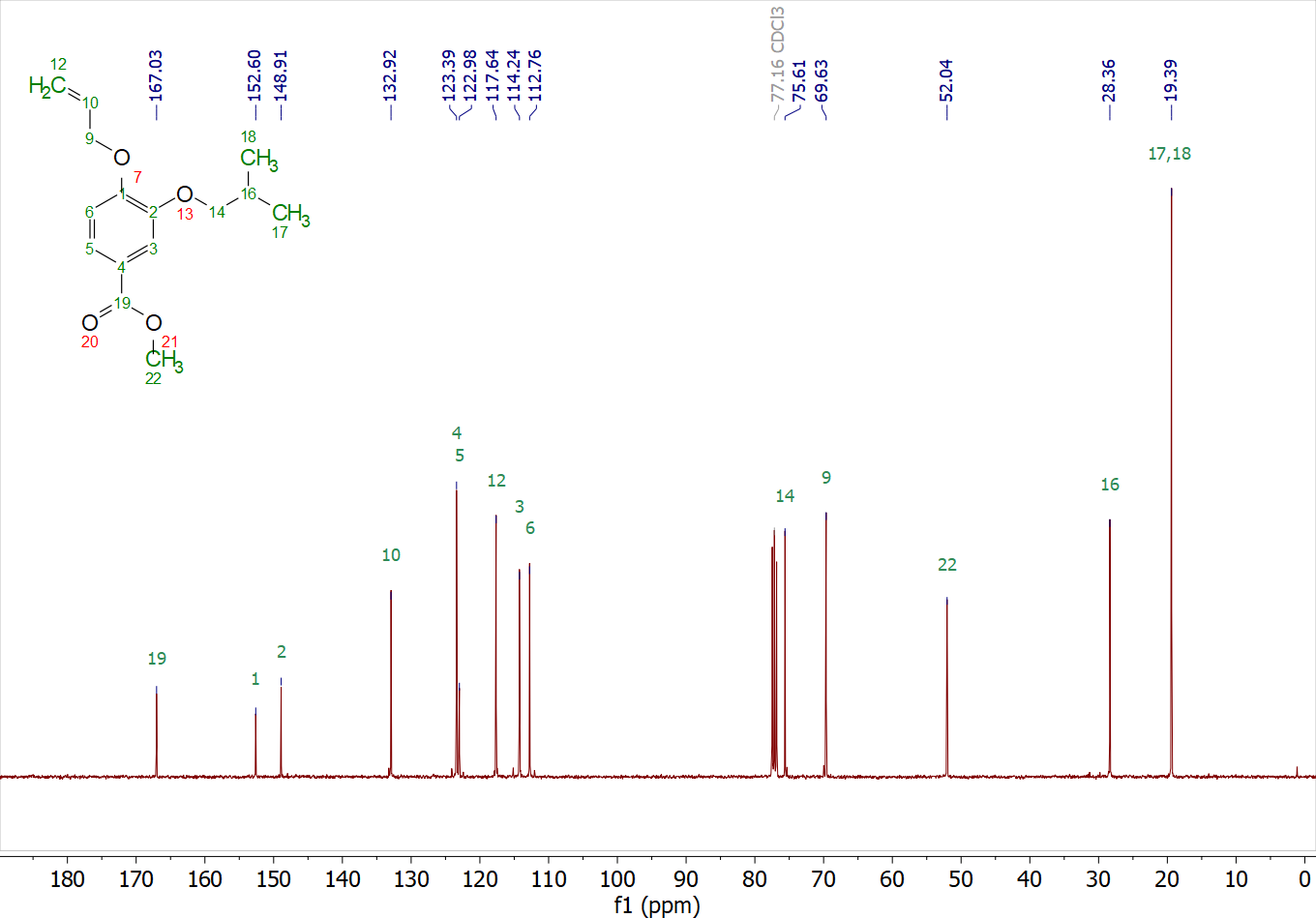
_

^13^C NMR Spectra of Compound **4** in CDCl_3_

_
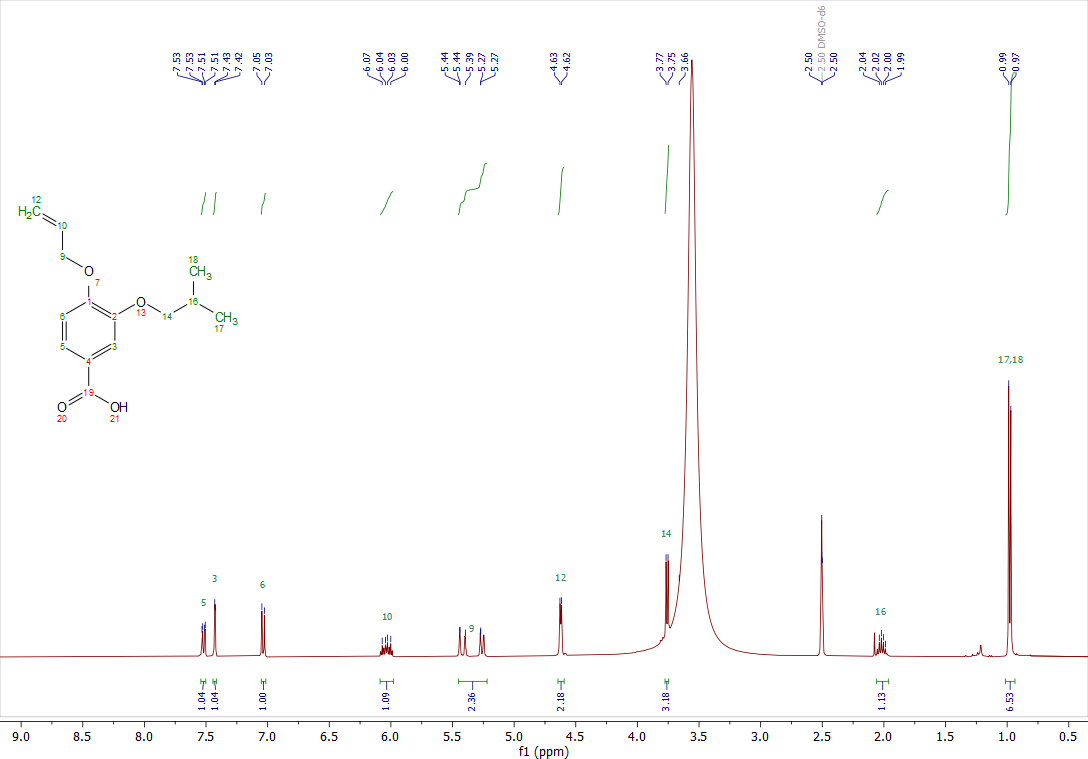
_

^1^H NMR Spectra of Compound **5** in DMSO-d_6_


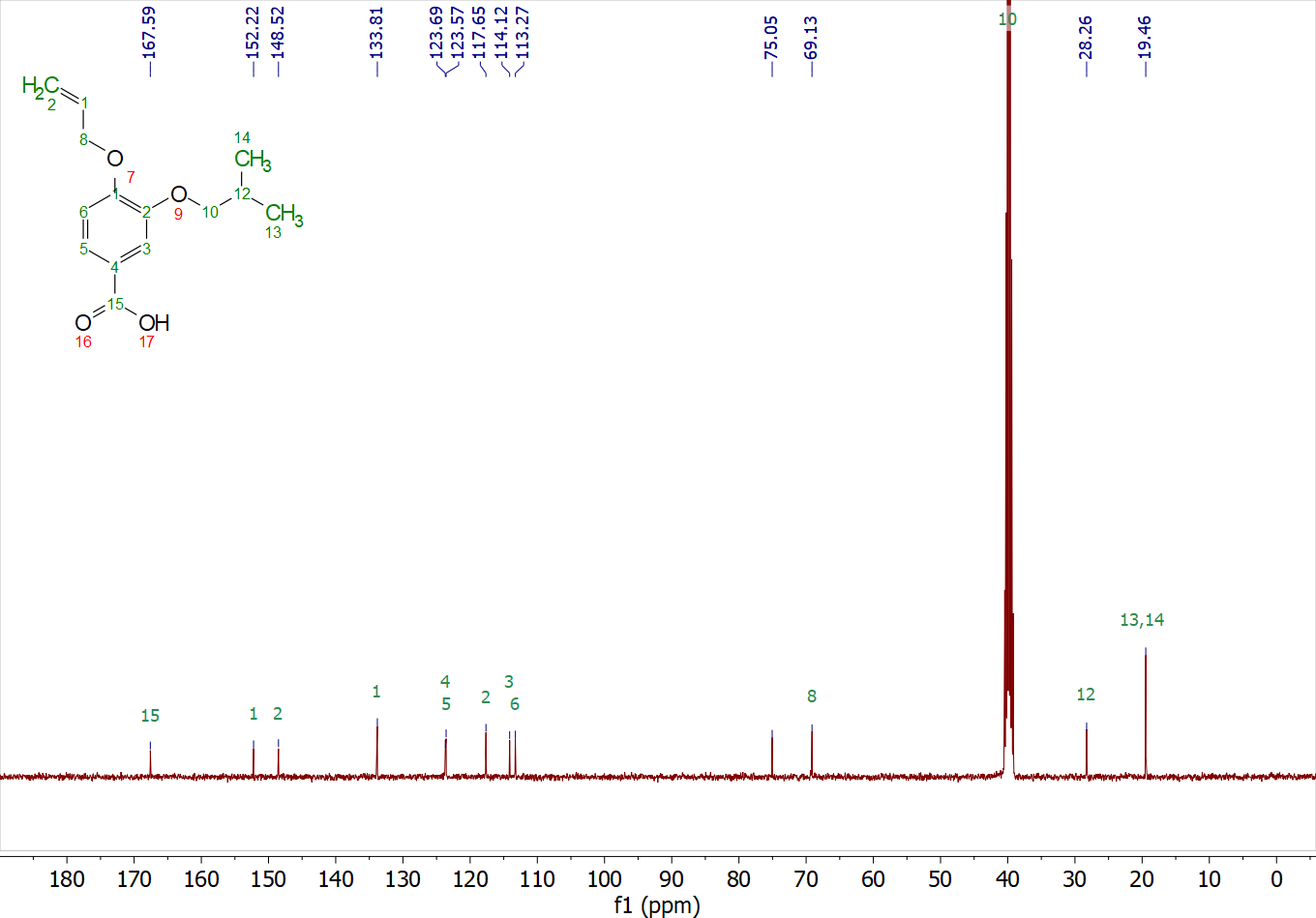


^13^C NMR Spectra of Compound **5** in DMSO-d_6_


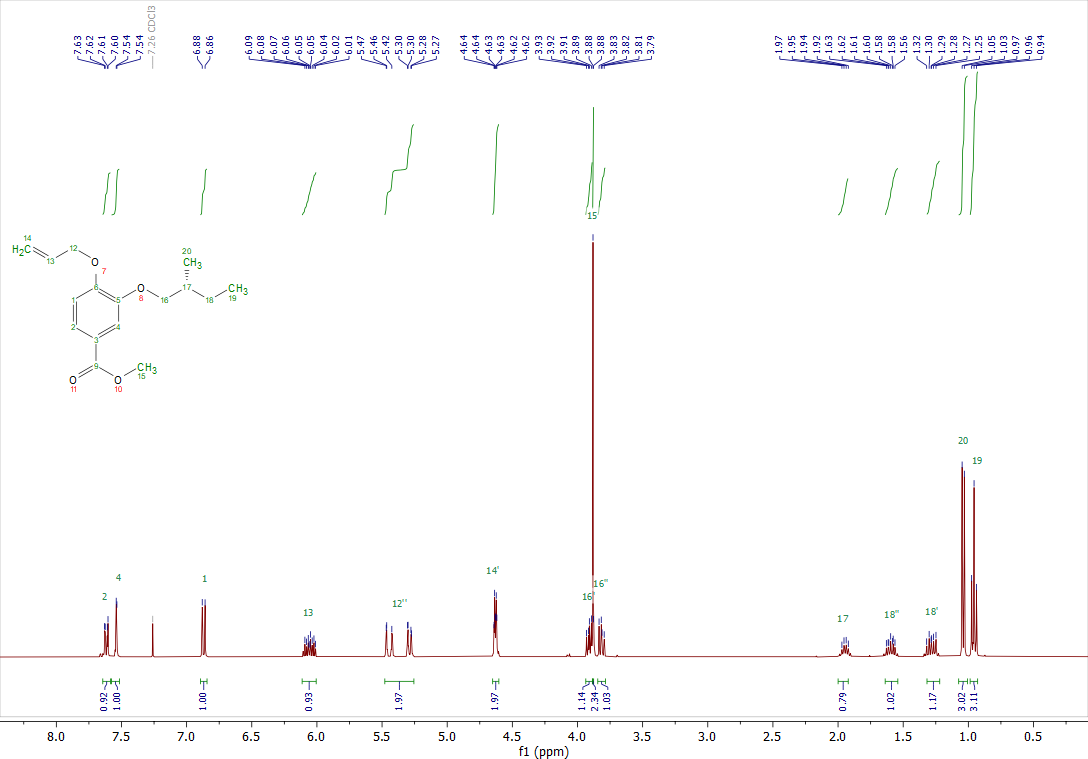


^1^H NMR Spectra of Compound **6** in CDCl_3_


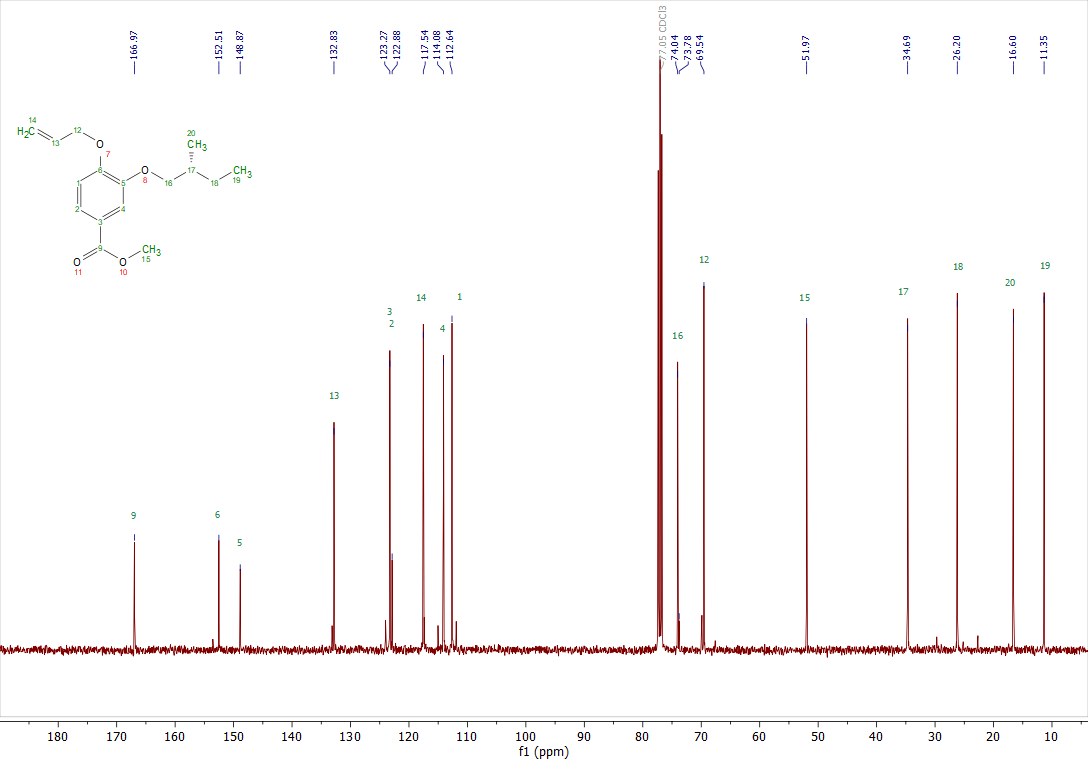


^13^C NMR Spectra of Compound **6** in CDCl_3_

_
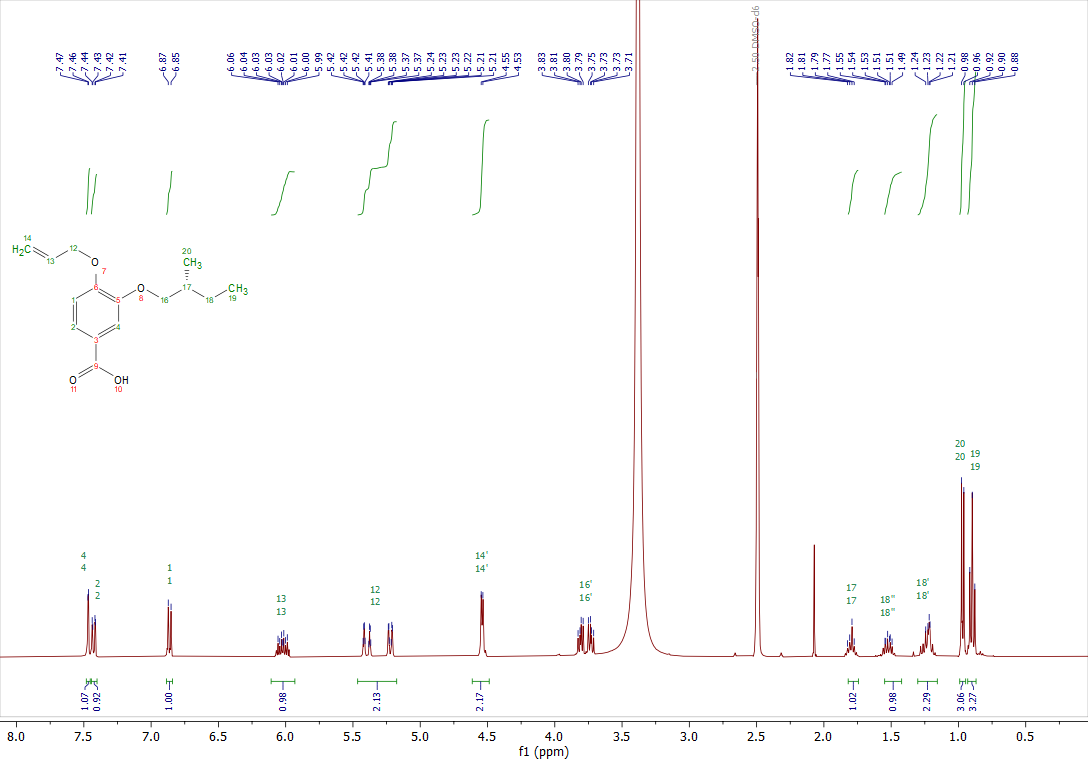
_

^1^H NMR Spectra of Compound **7** in DMSO-d_6_


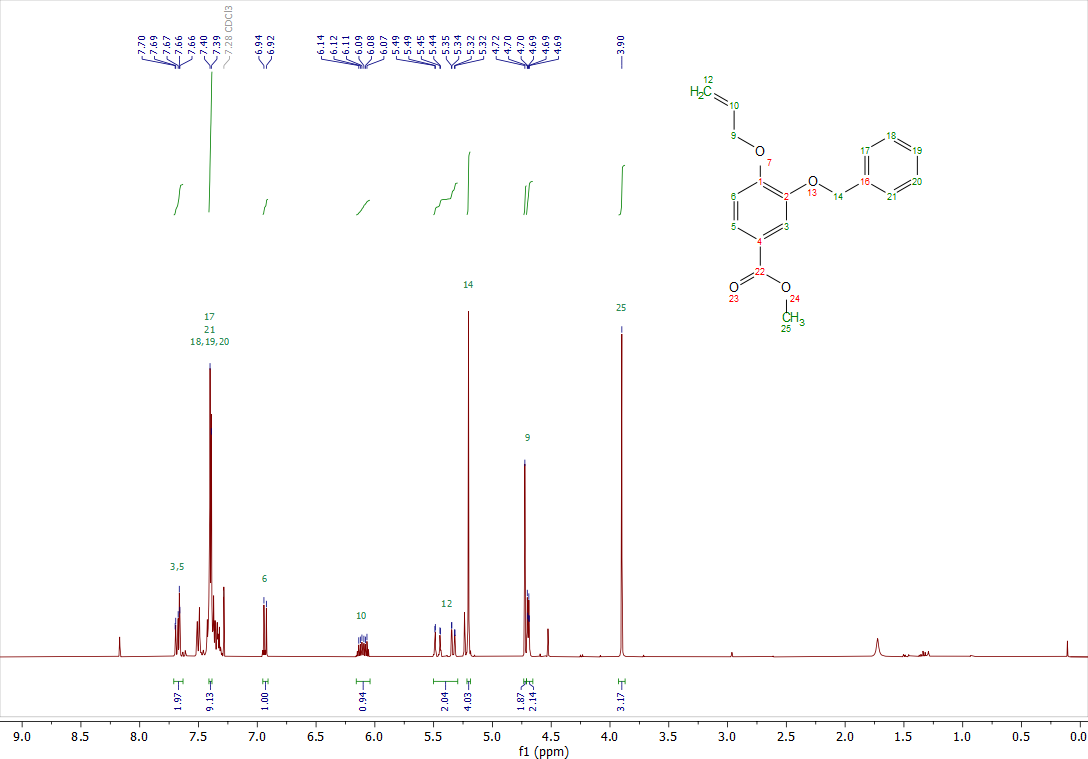


^1^H NMR Spectra of Compound **8** in CDCl_3_

_
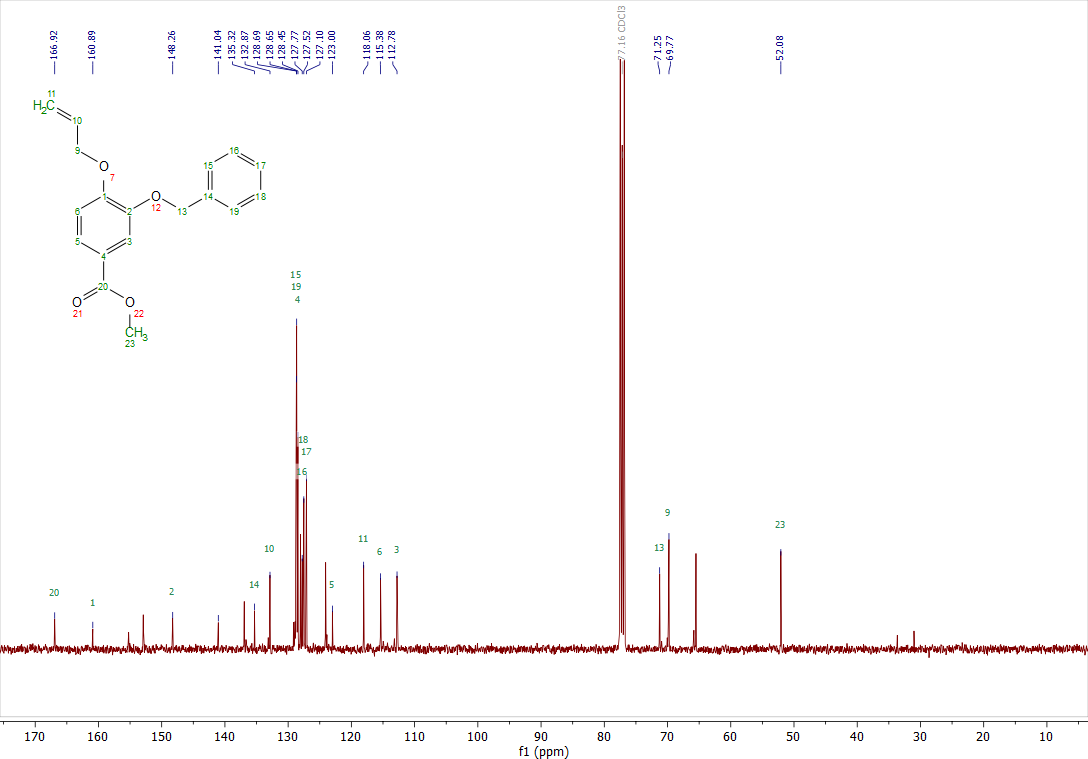
_

^13^C NMR Spectra of Compound **8** in CDCl_3_

_
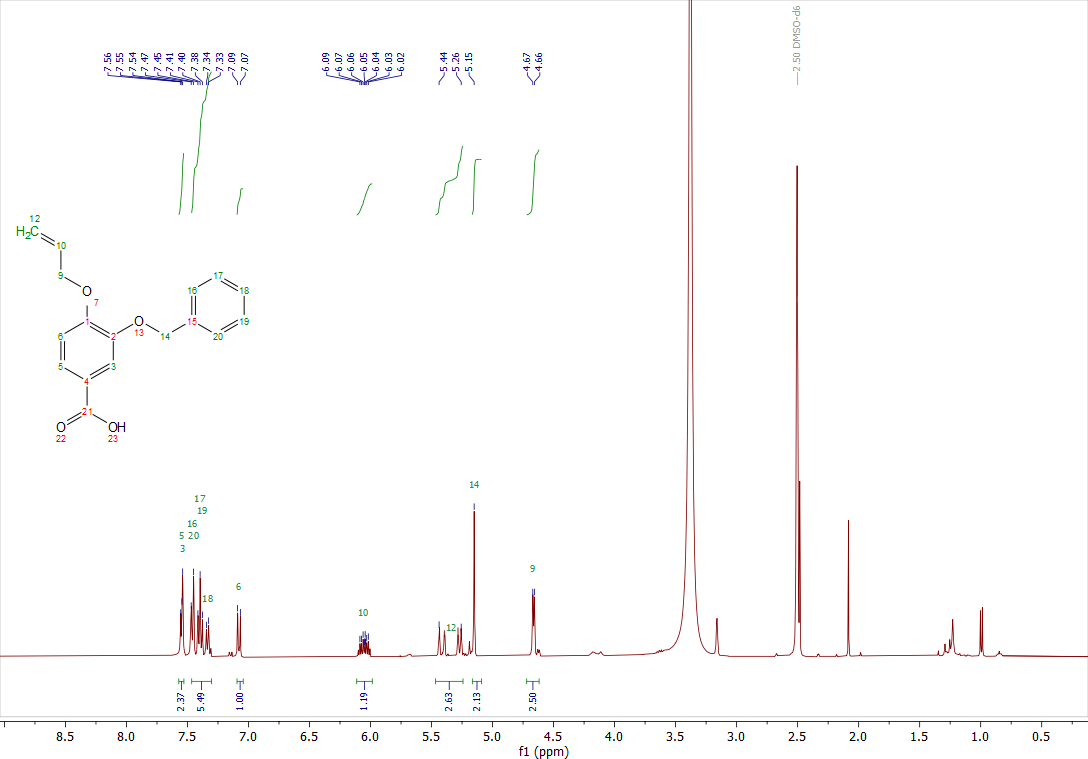
_

^1^H NMR Spectra of Compound **9** in DMSO-d_6_

_
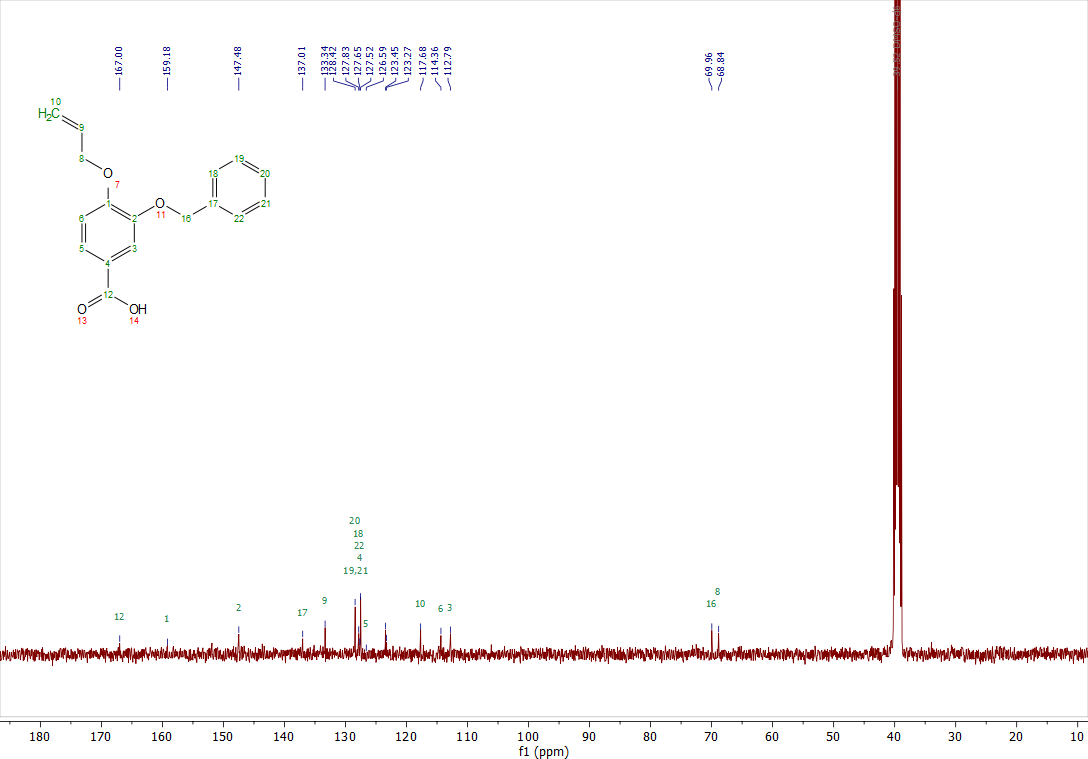
_

^13^C NMR Spectra of Compound **9** in DMSO-d_6_

_
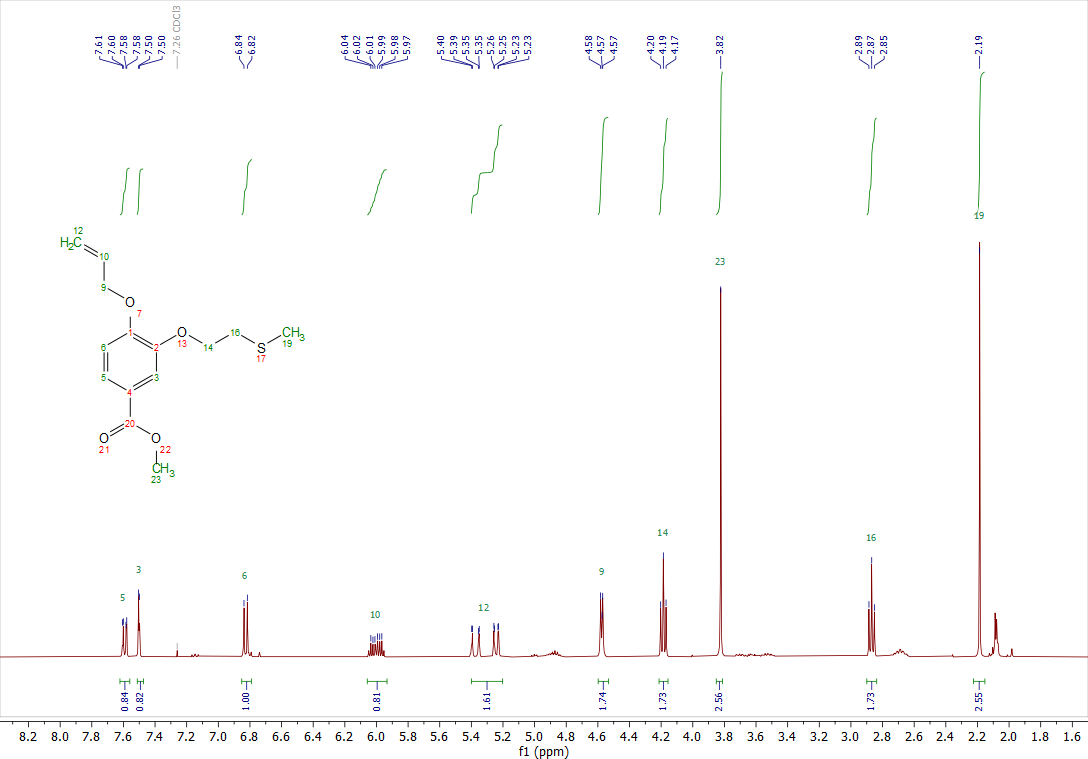
_

^1^H NMR Spectra of Compound **10** in CDCl_3_


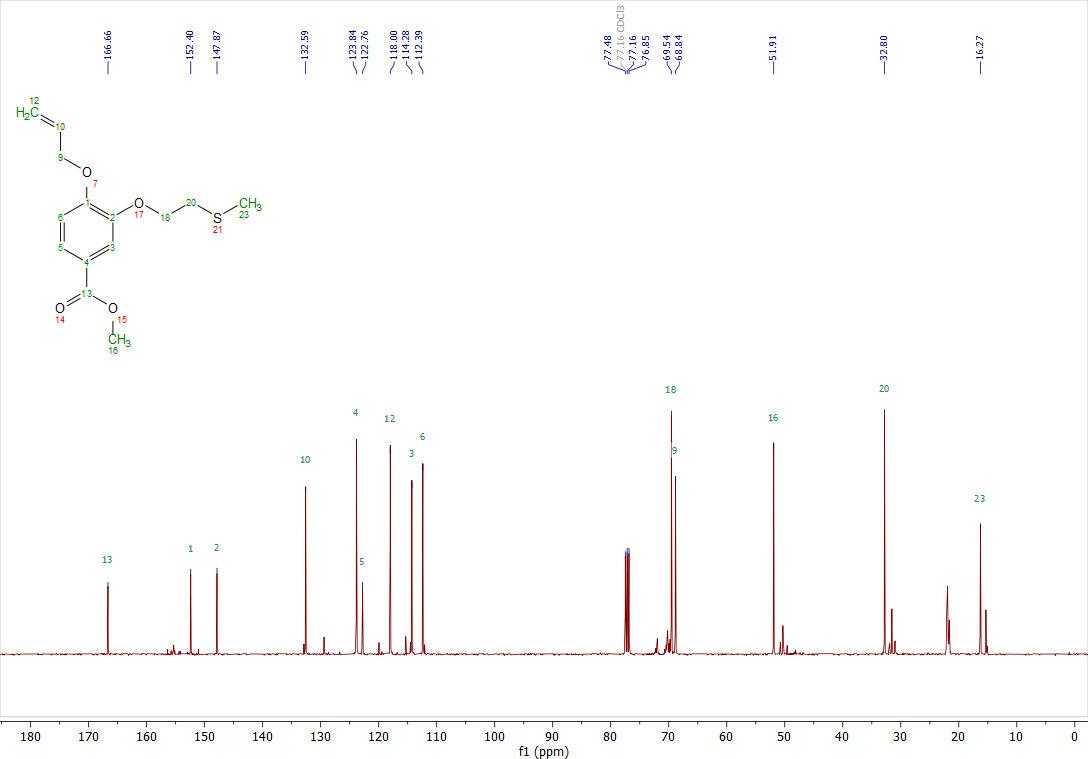


^13^C NMR Spectra of Compound **10** in CDCl_3_

_
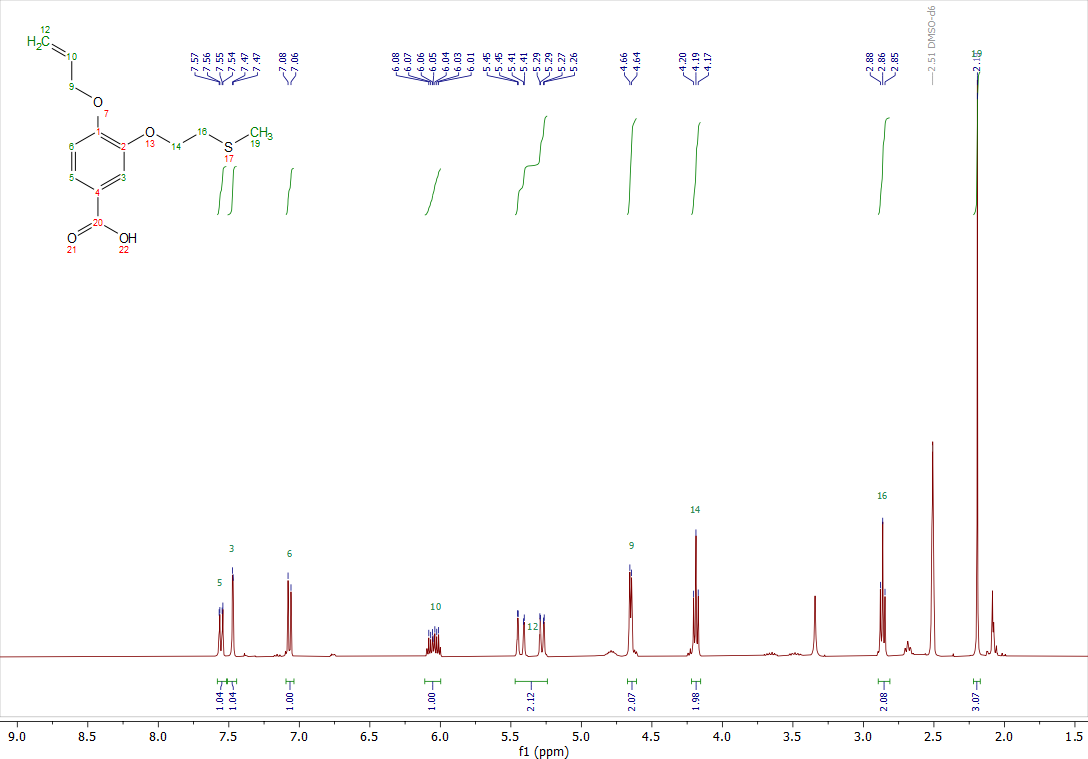
_

^1^H NMR Spectra of Compound **11** in DMSO-d_6_

_
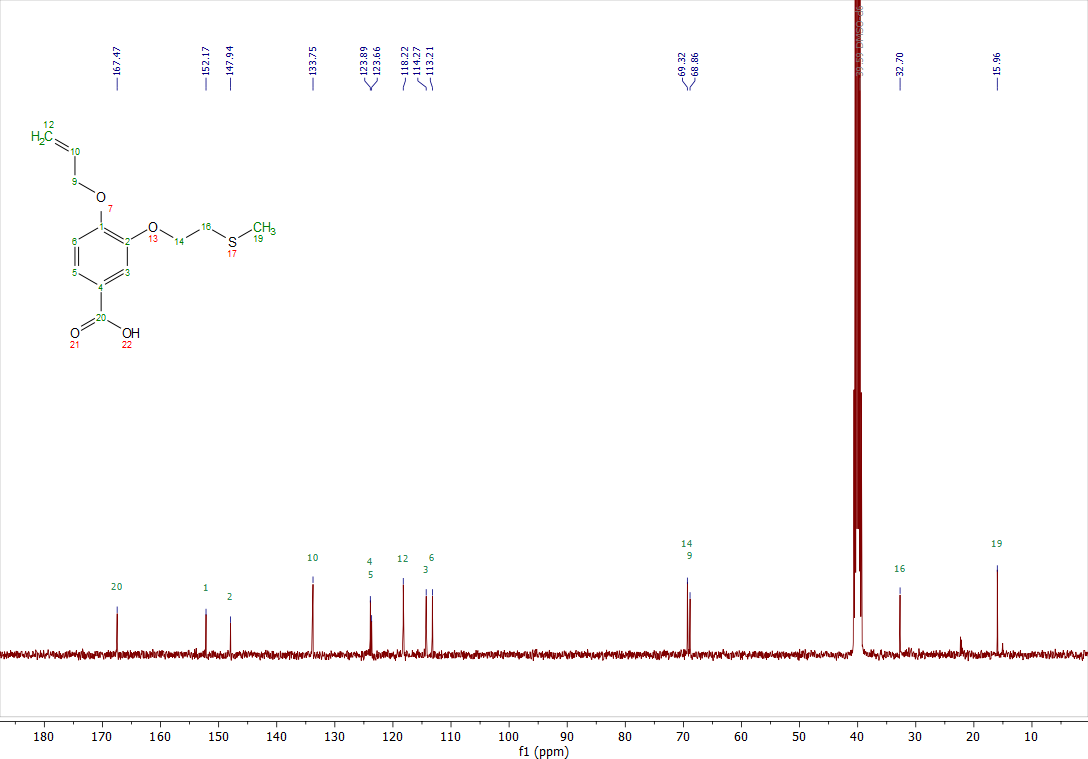
_

^13^C NMR Spectra of Compound **11** in DMSO-d_6_


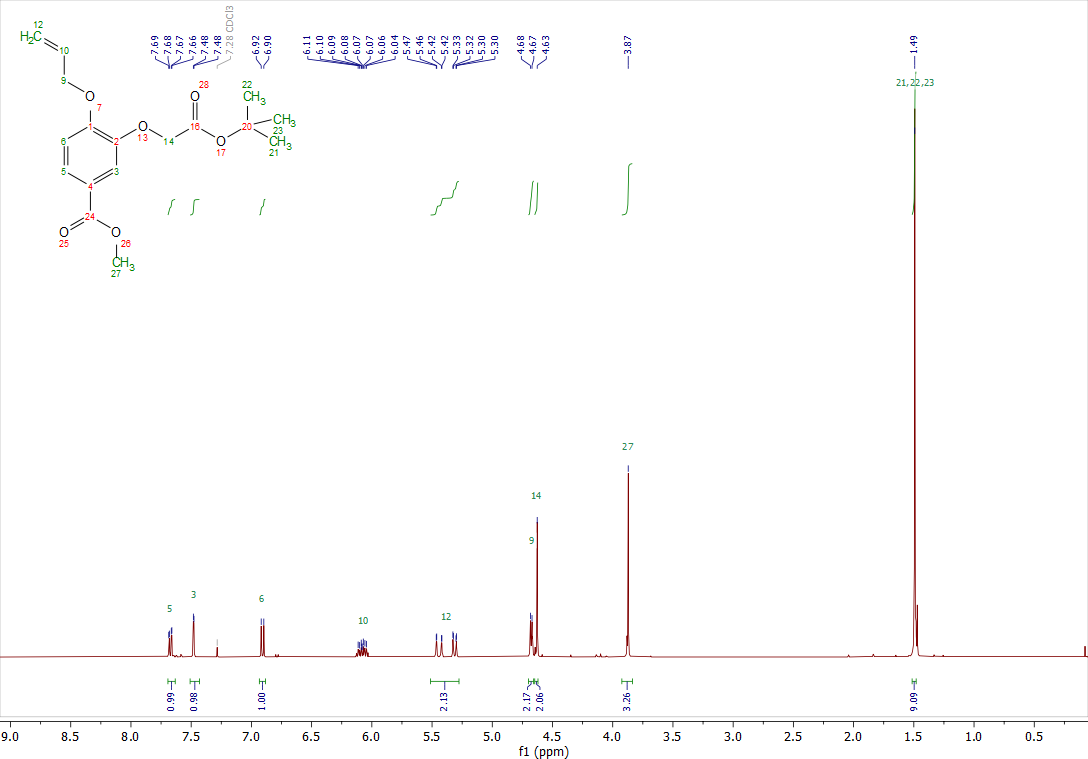


^1^H NMR Spectra of Compound **12** in CDCl_3_

_
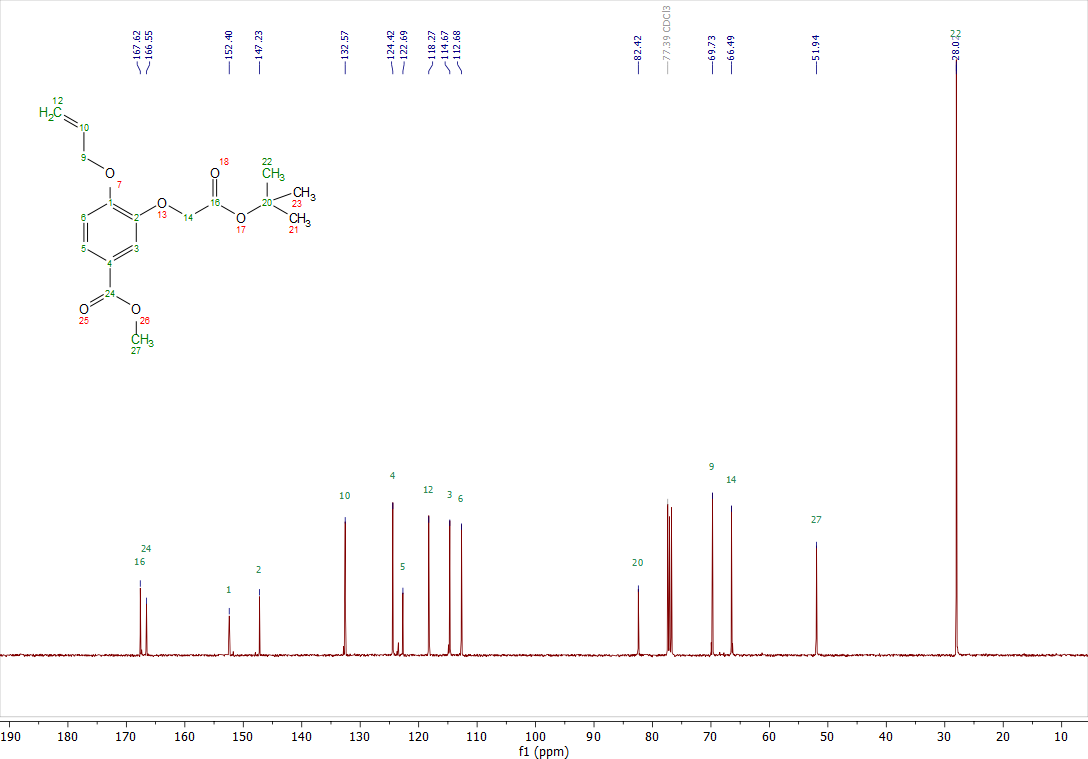
_

^13^C NMR Spectra of Compound **12** in CDCl_3_


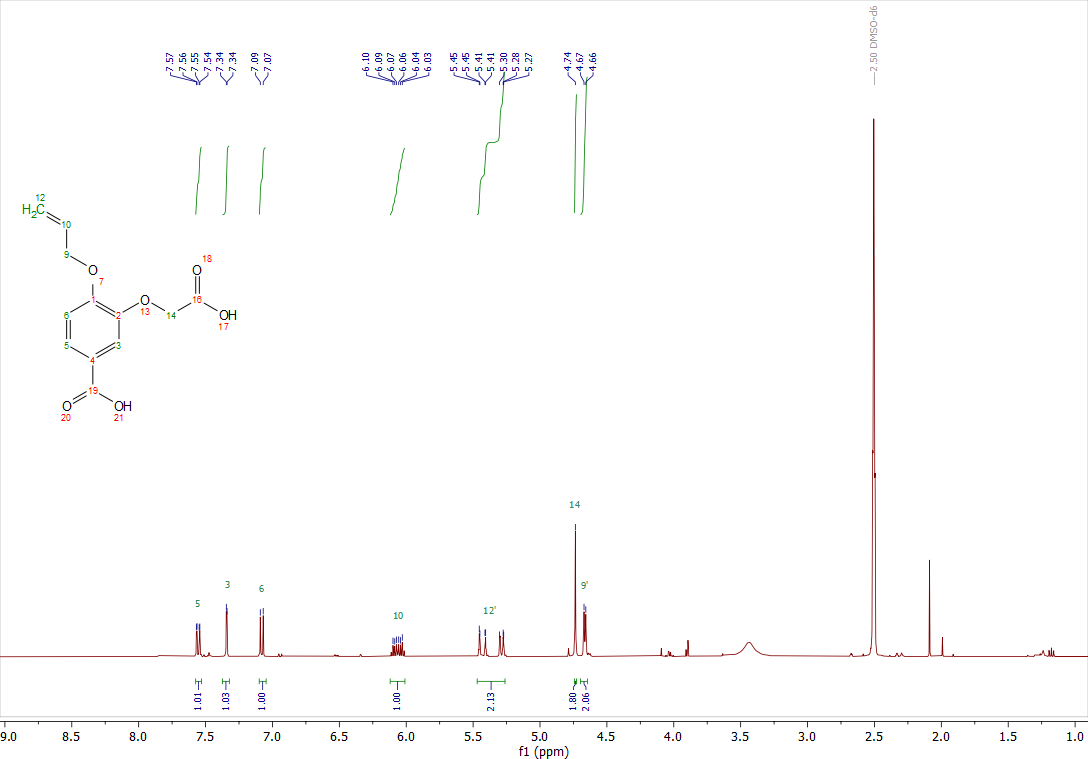


^1^H NMR Spectra of Compound **13** in DMSO-d_6_


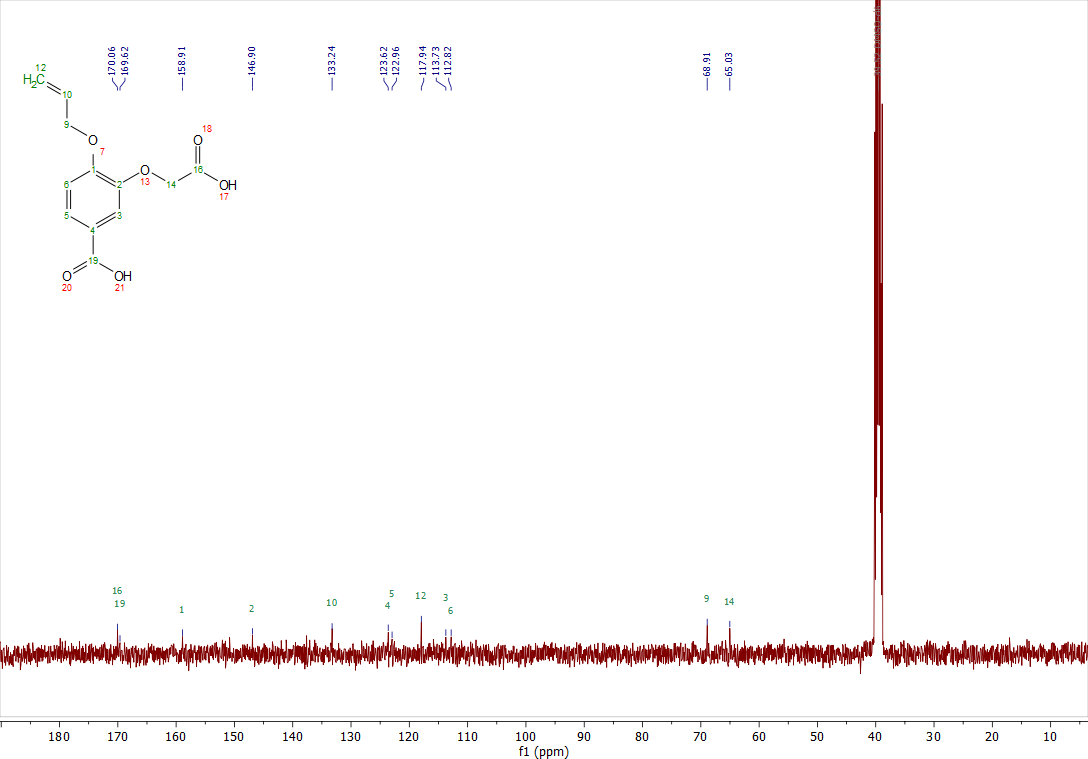


^13^C NMR Spectra of Compound **13** in DMSO-d_6_


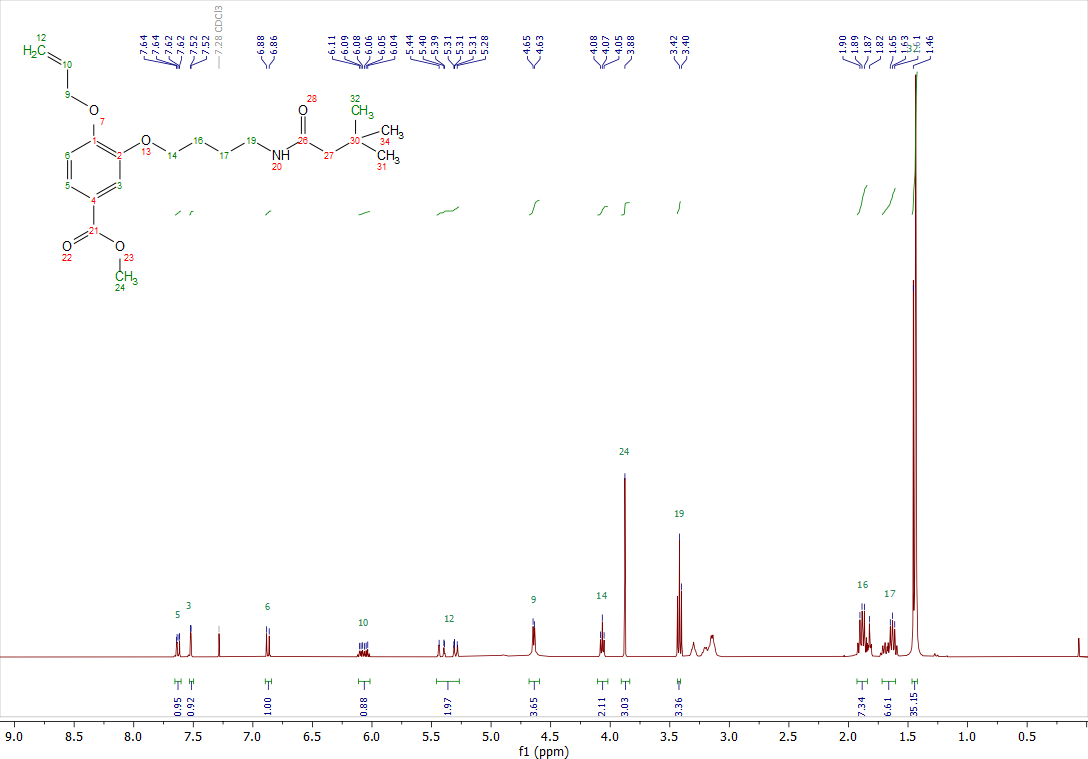


^1^H NMR Spectra of Compound **14** in CDCl_3_

_
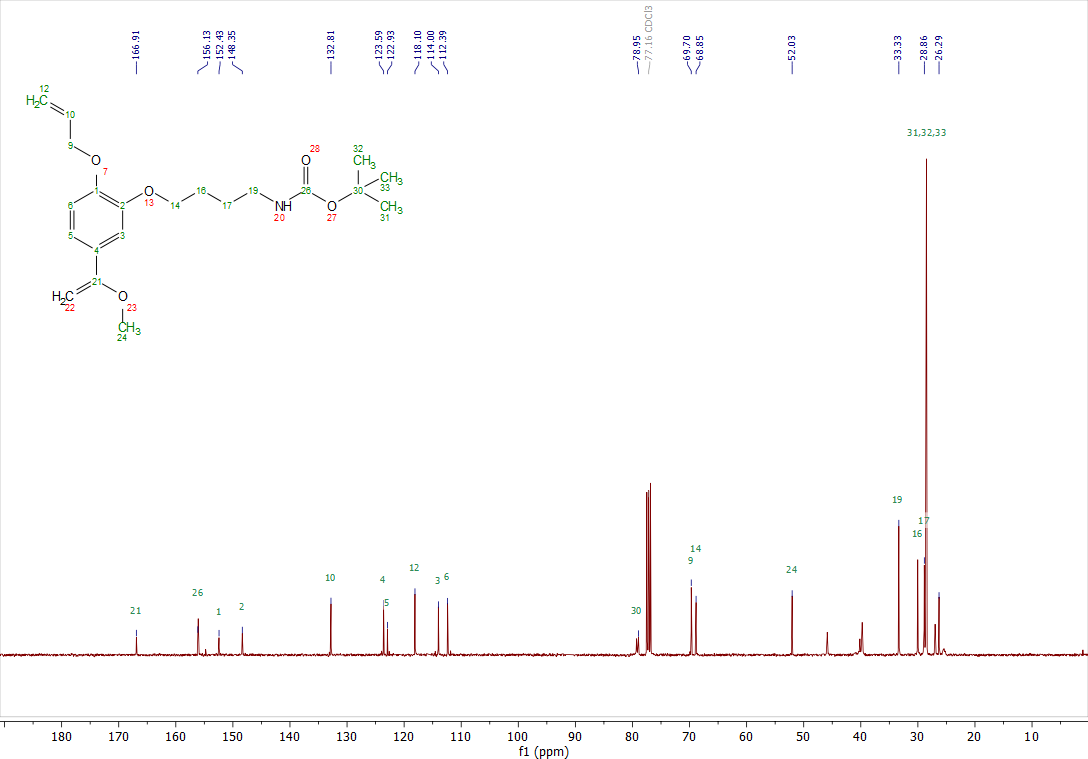
_

^13^C NMR Spectra of Compound **14** in CDCl_3_


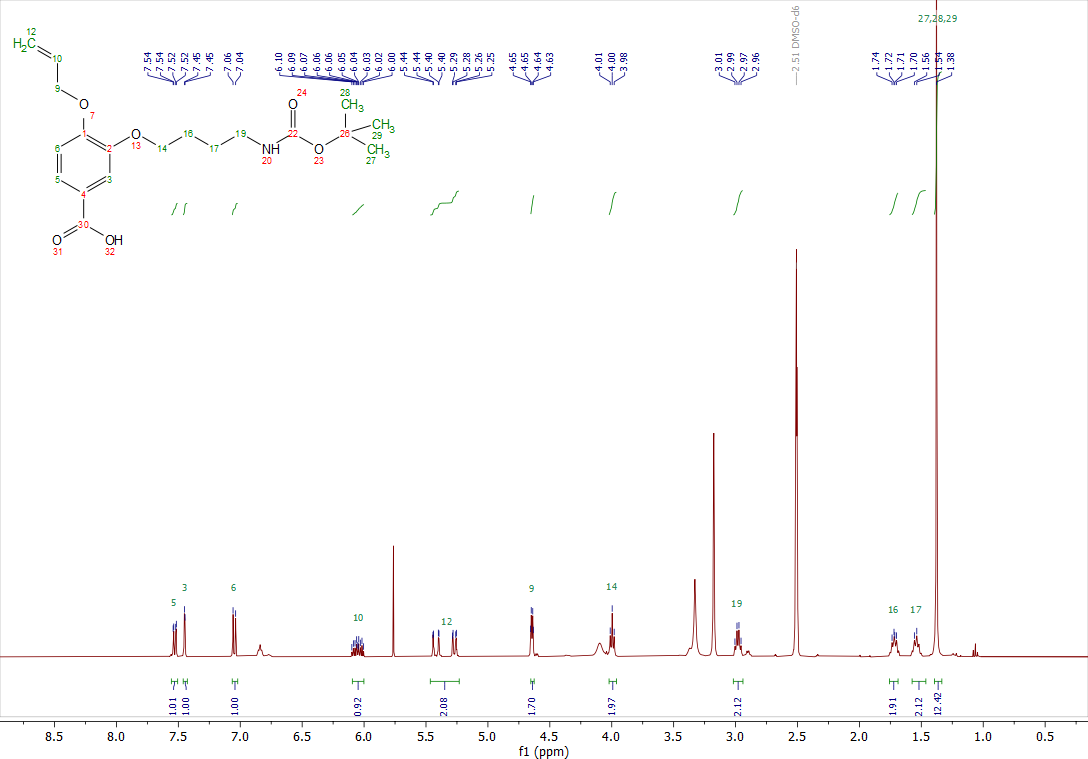


^1^H NMR Spectra of Compound **15** in DMSO-d_6_

_
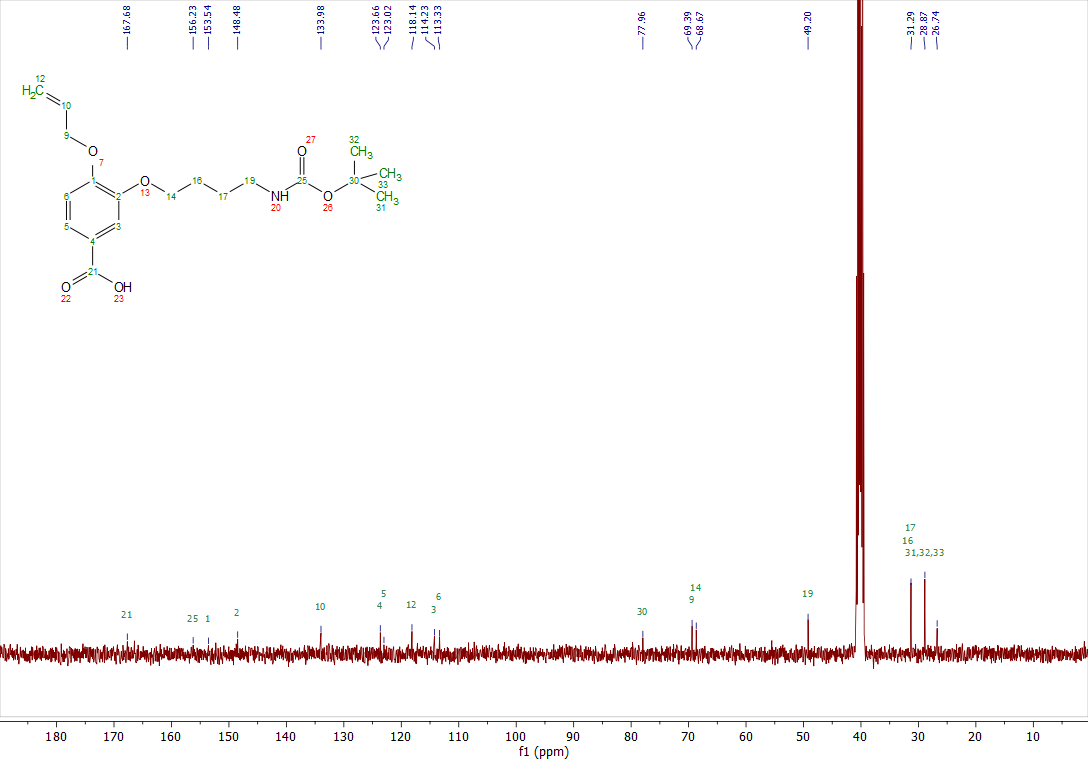
_

^13^C NMR Spectra of Compound **15** in DMSO-d_6_

_
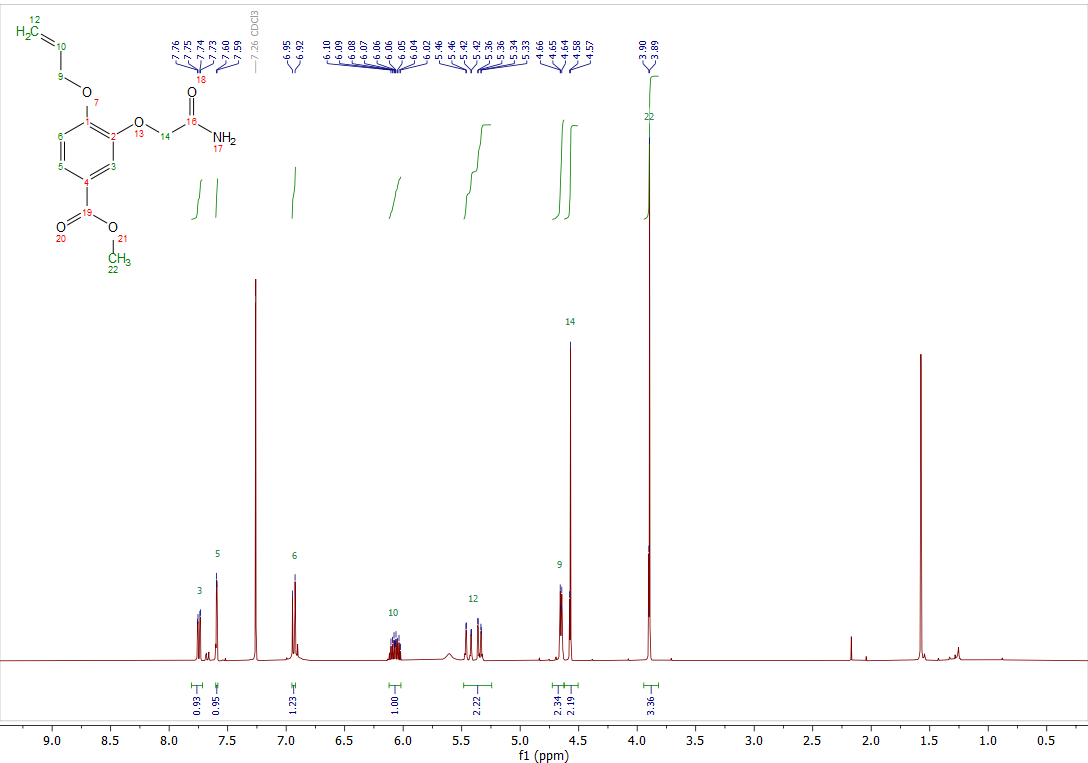
_

^1^H NMR Spectra of Compound **16** in CDCl_3_

_
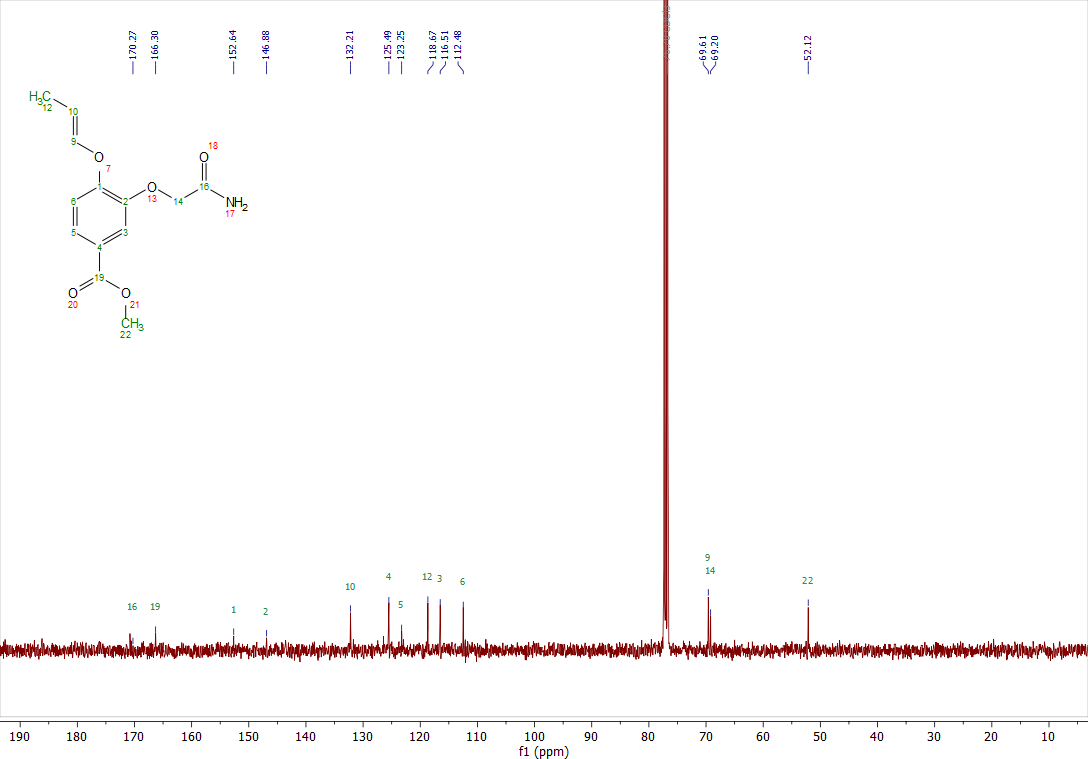
_

^13^C NMR Spectra of Compound **16** in CDCl_3_

_
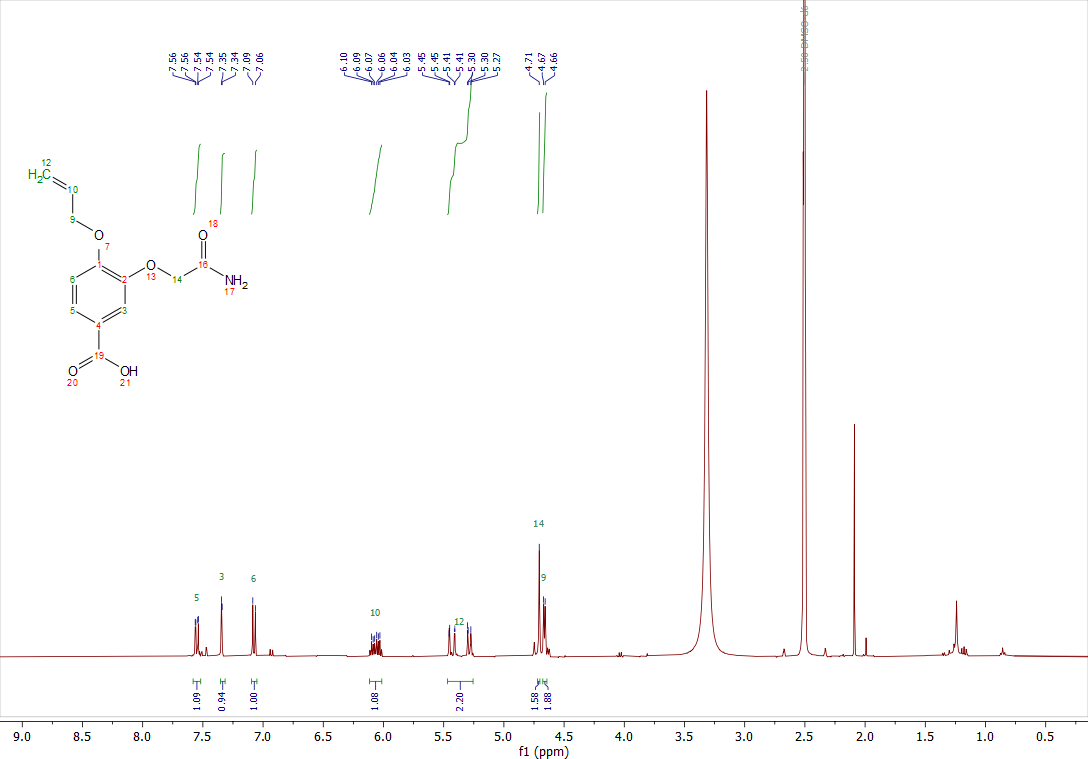
_

^1^H NMR Spectra of Compound **17** in DMSO-d_6_

_
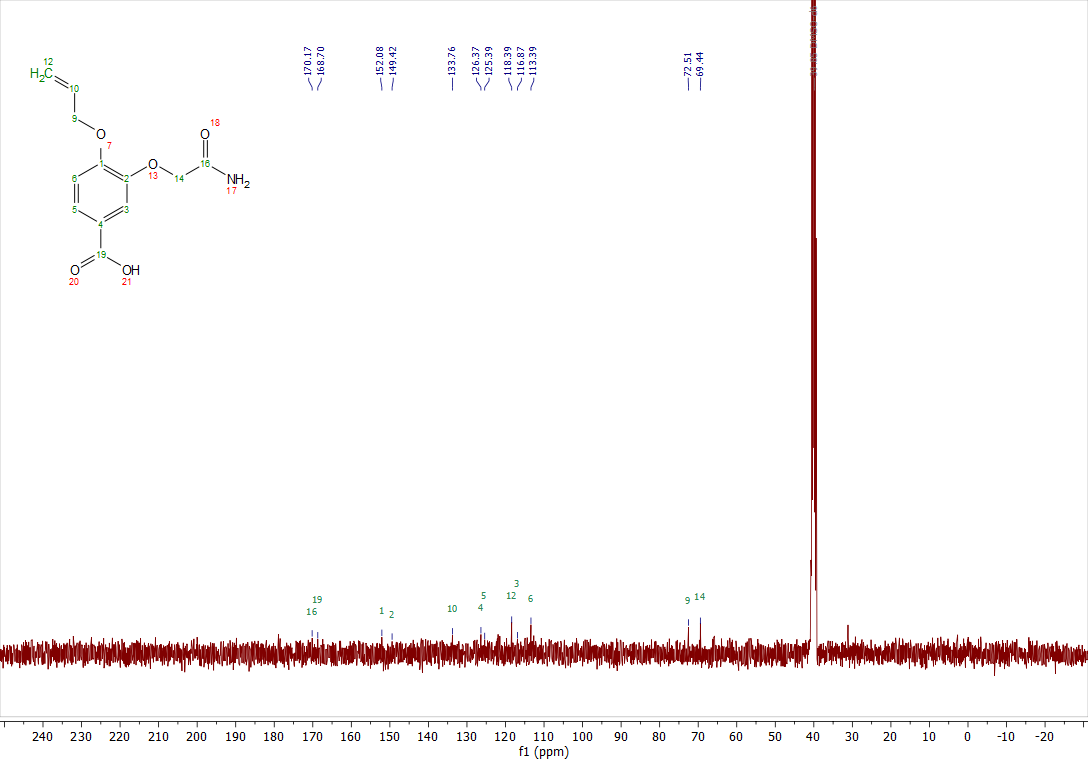
_

^13^C NMR Spectra of Compound **17** in DMSO-d_6_

_
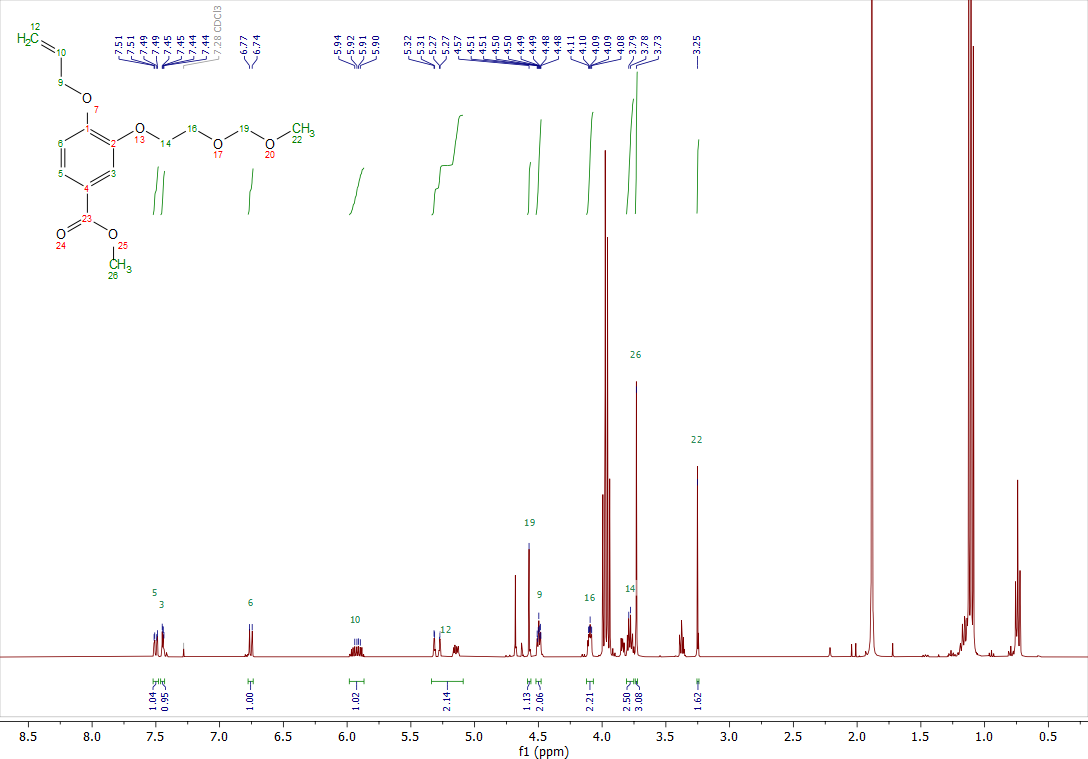
_

^1^H NMR Spectra of Compound **18** in CDCl_3_

_
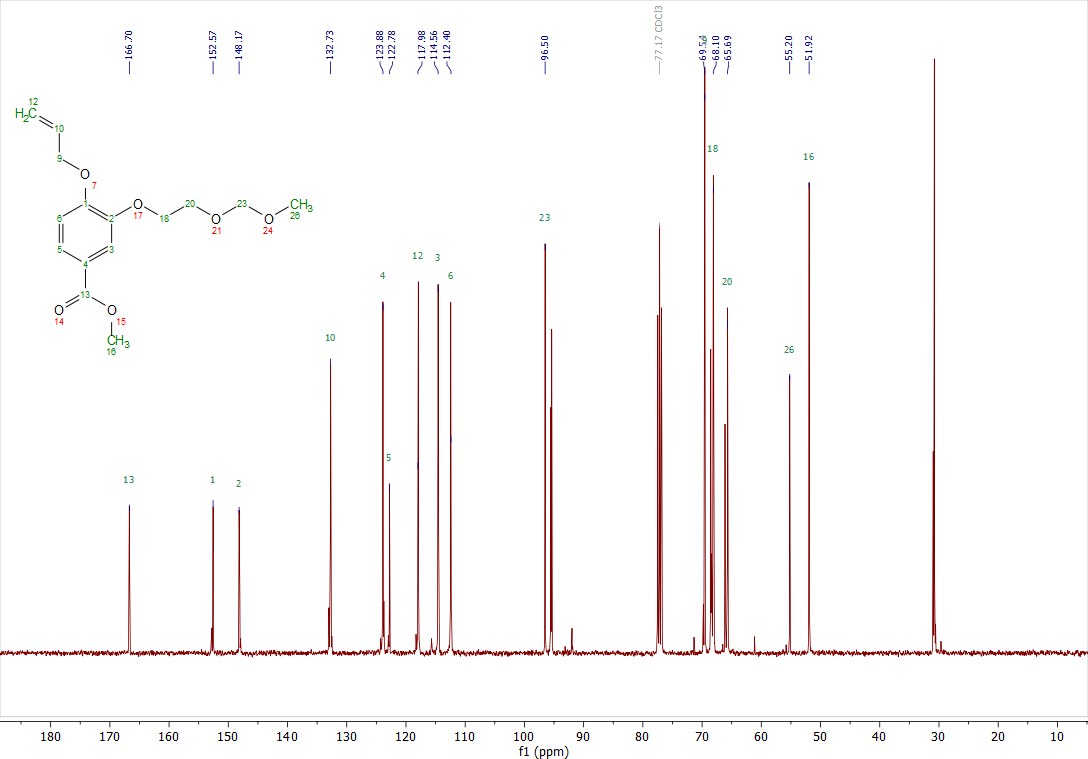
_

^13^C NMR Spectra of Compound **18** in CDCl_3_

_
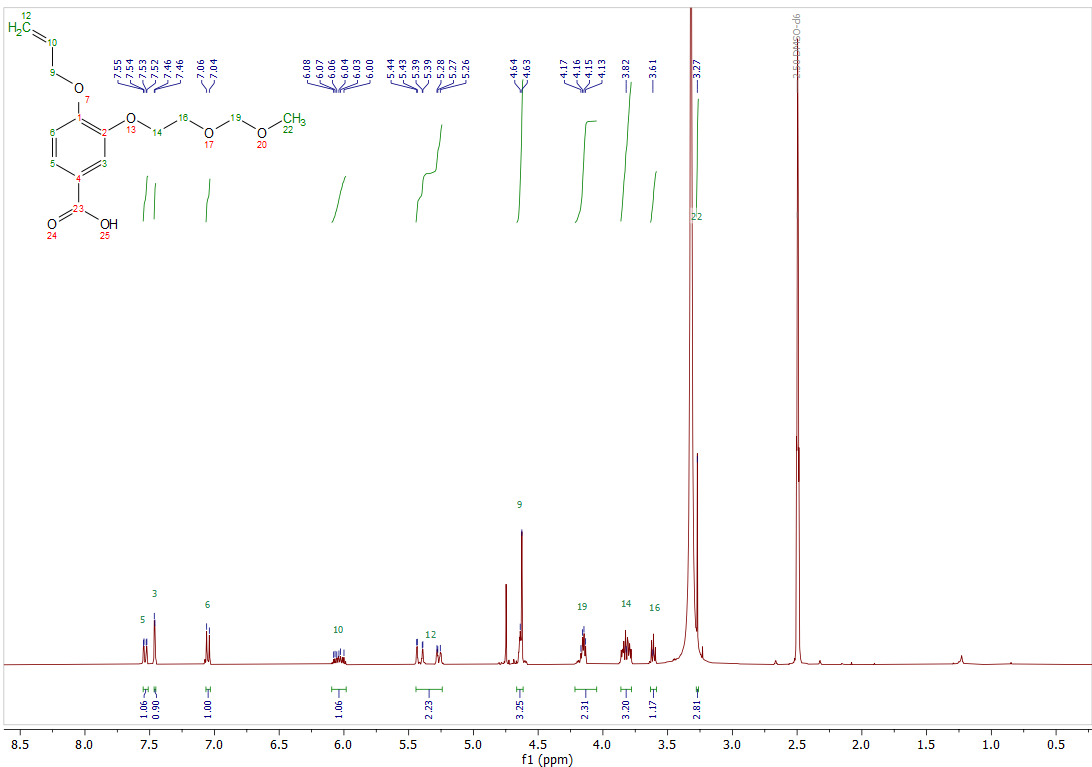
_

^1^H NMR Spectra of Compound **19** in DMSO-d_6_

_
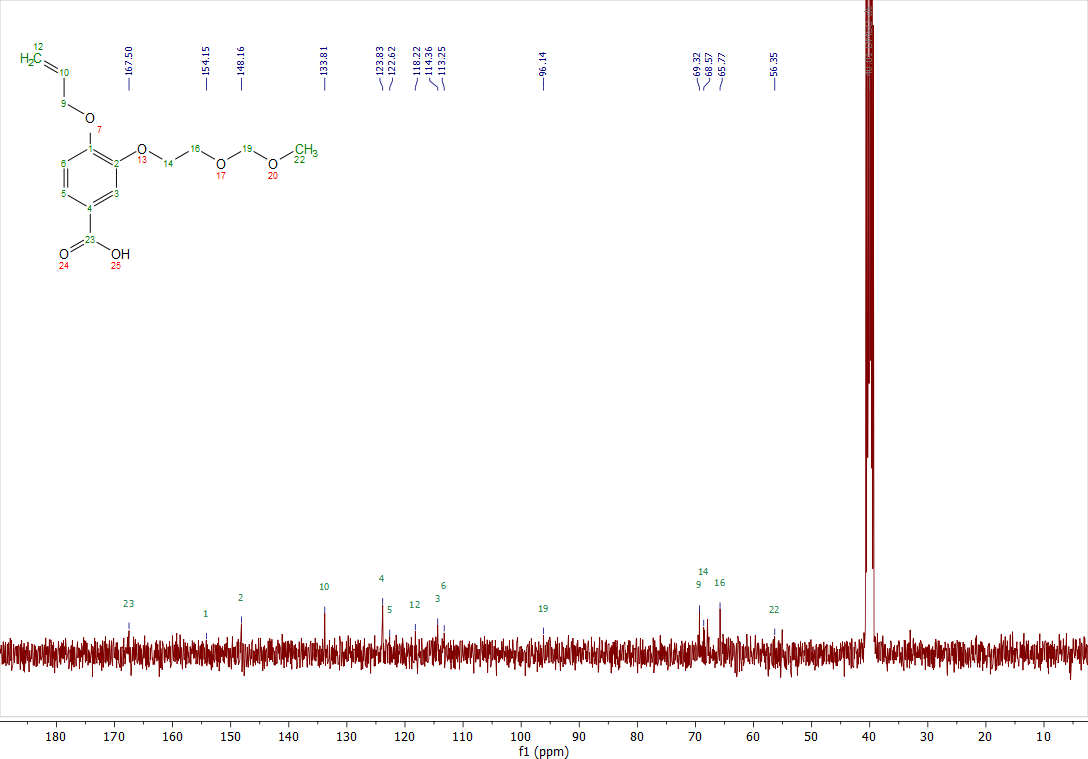
_

^13^C NMR Spectra of Compound **19** in DMSO-d_6_

_
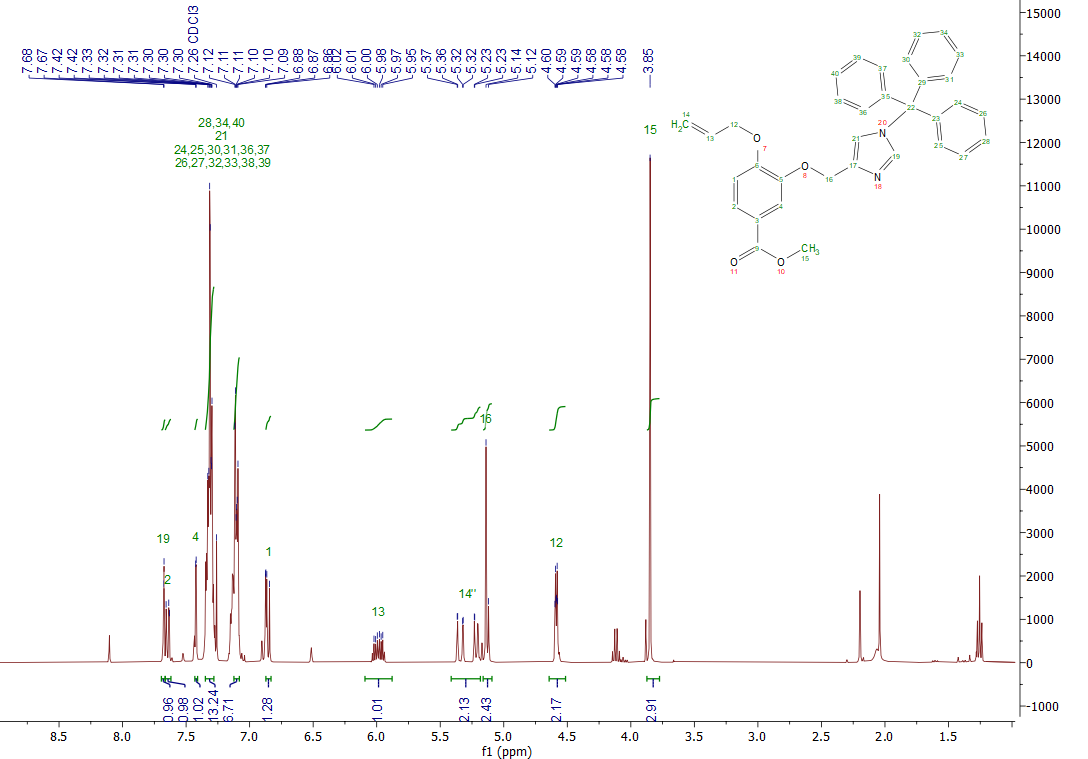
_

^1^H NMR Spectra of Compound **20** in CDCl_3_

_
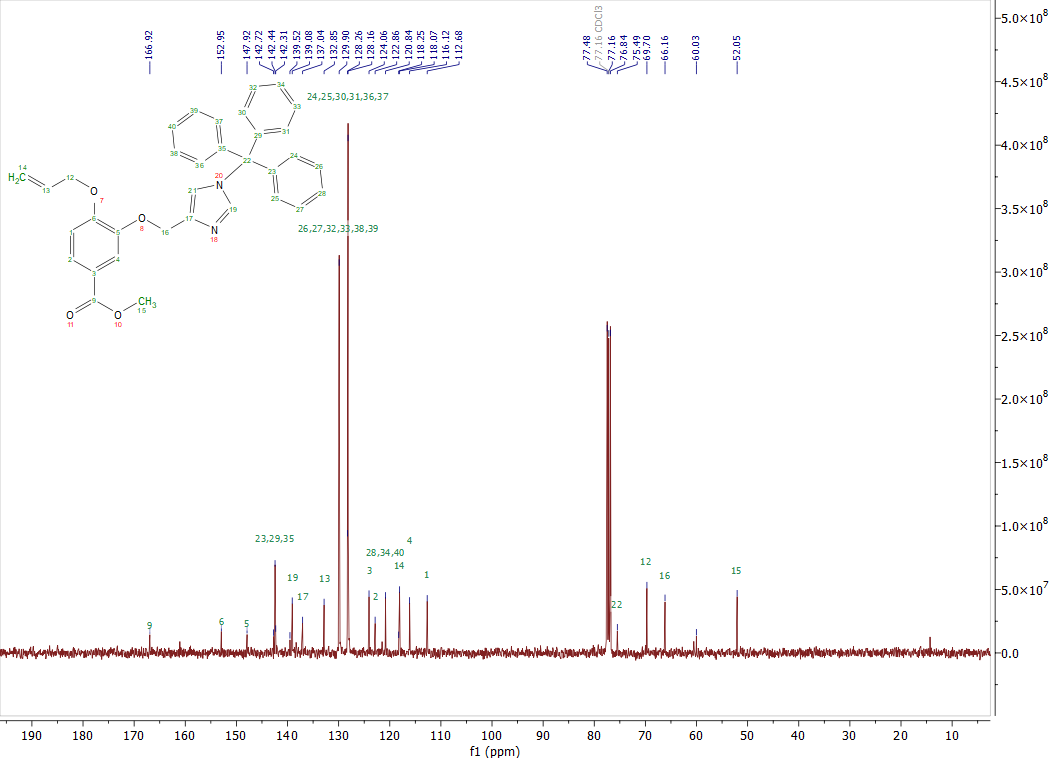
_

^13^C NMR Spectra of Compound **20** in CDCl_3_

_
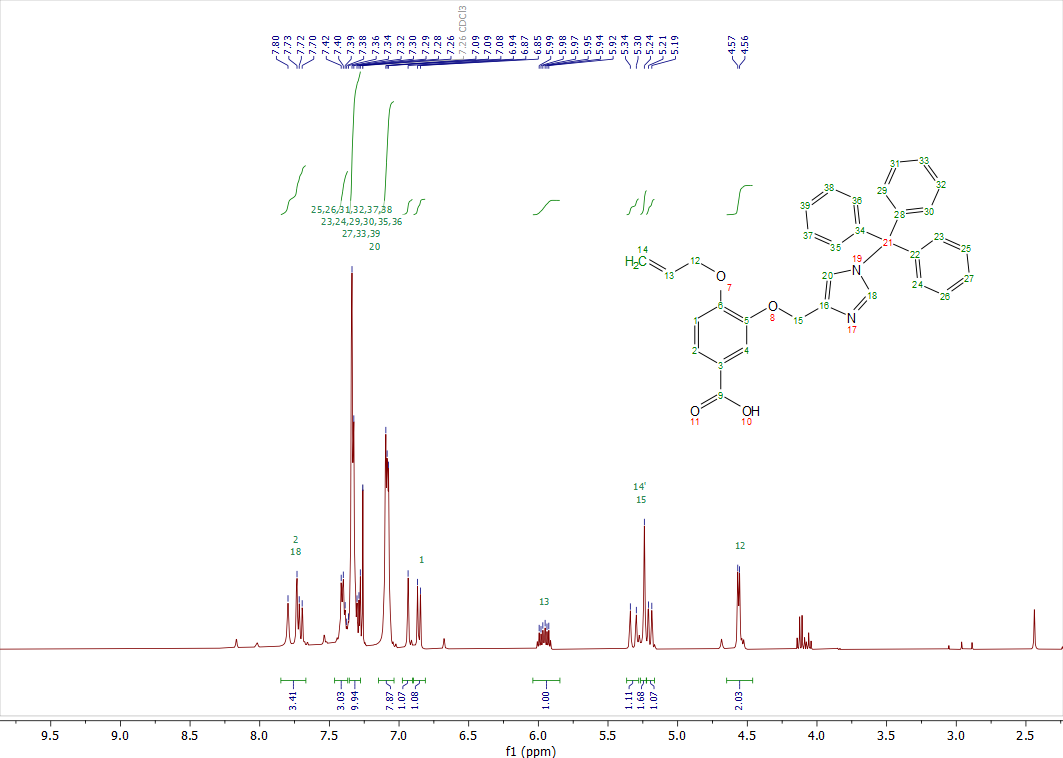
_

^1^H NMR Spectra of Compound **21** in CDCl_3_

_
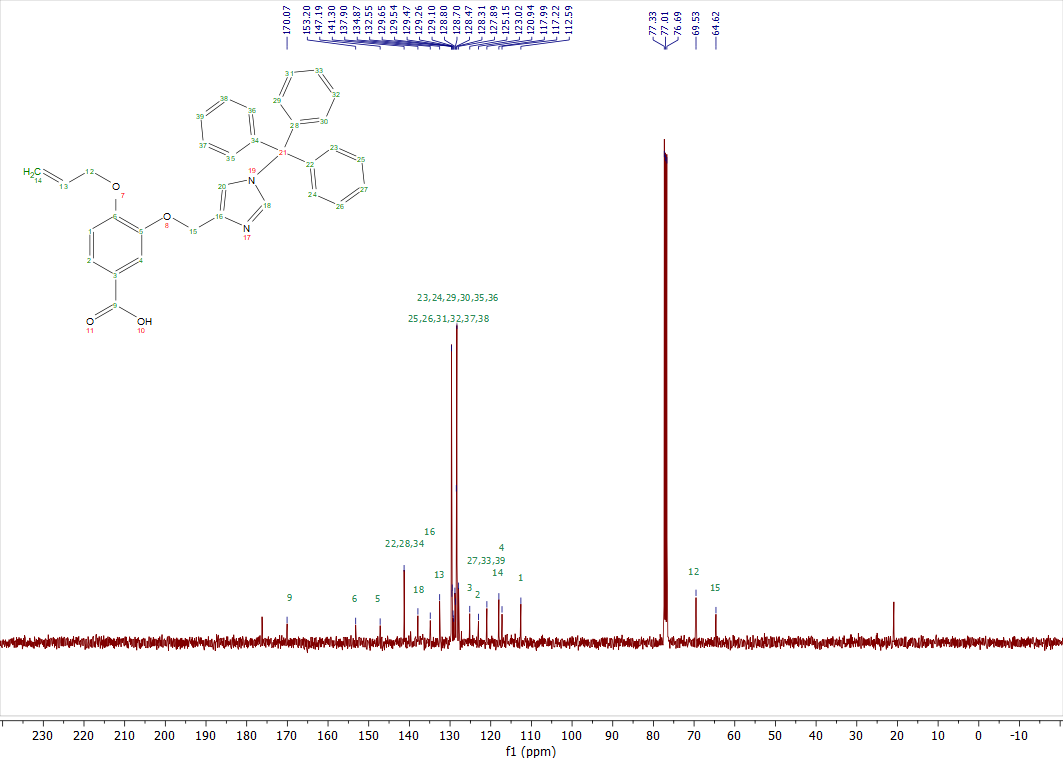
_

^13^C NMR Spectra of Compound **21** in CDCl_3_

_
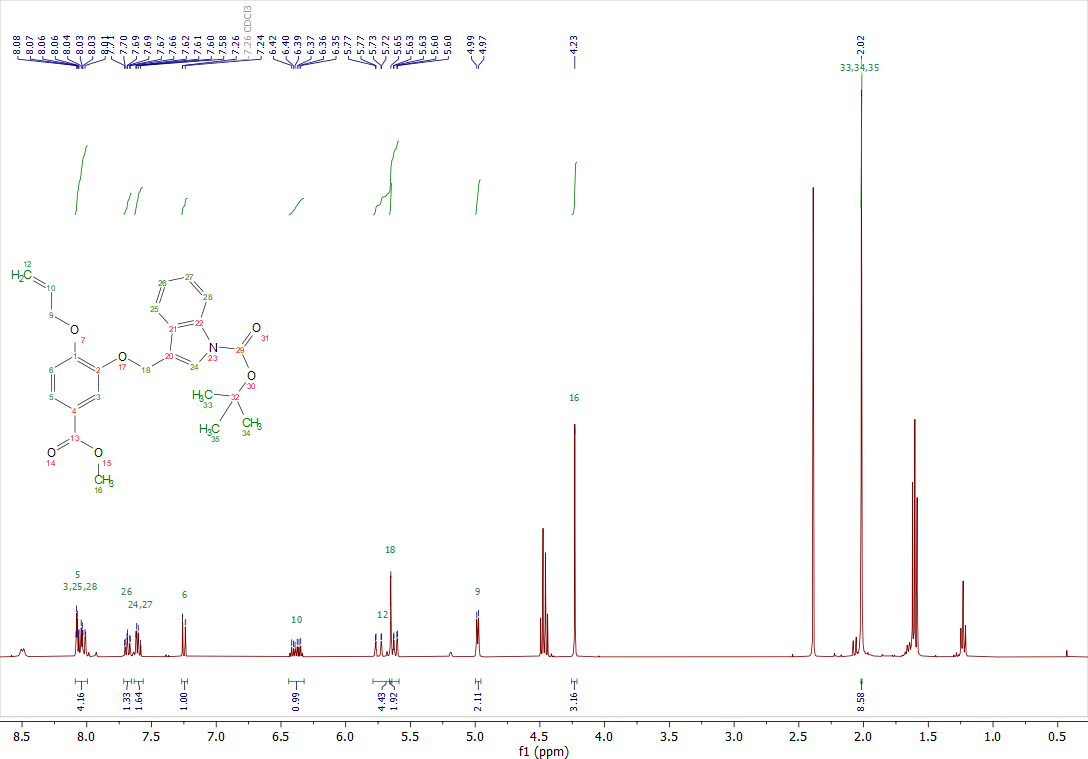
_

^1^H NMR Spectra of Compound **22** in CDCl_3_

_
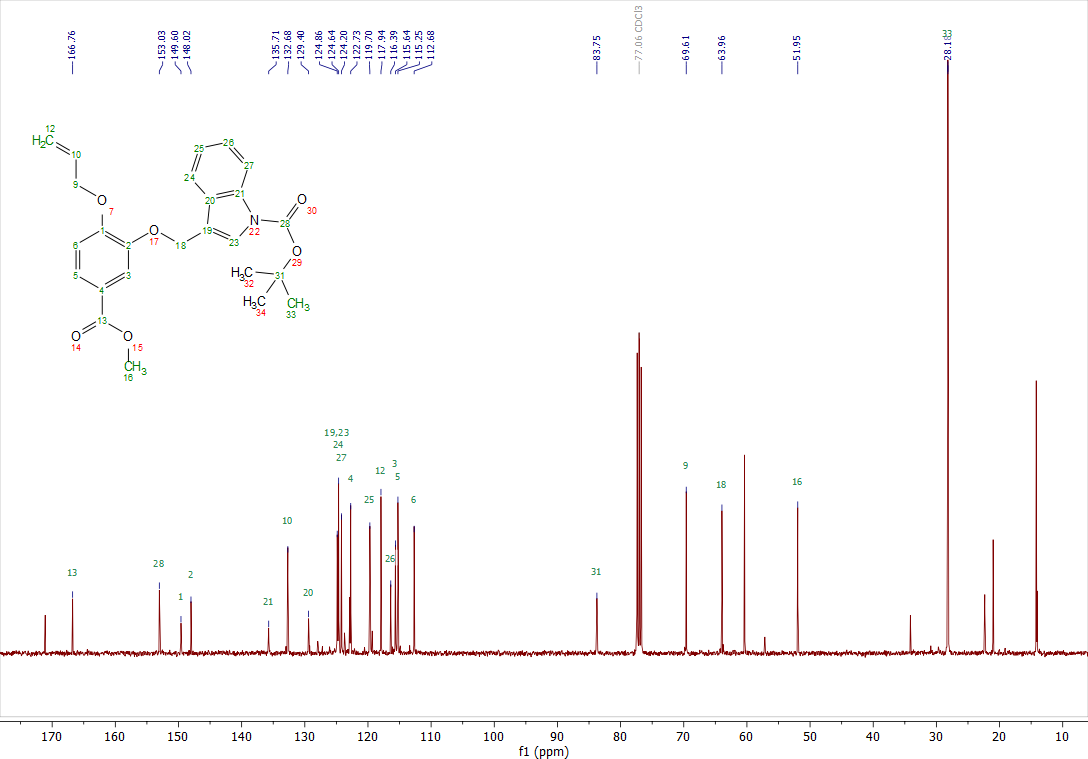
_

^13^C NMR Spectra of Compound **22** in CDCl_3_

_
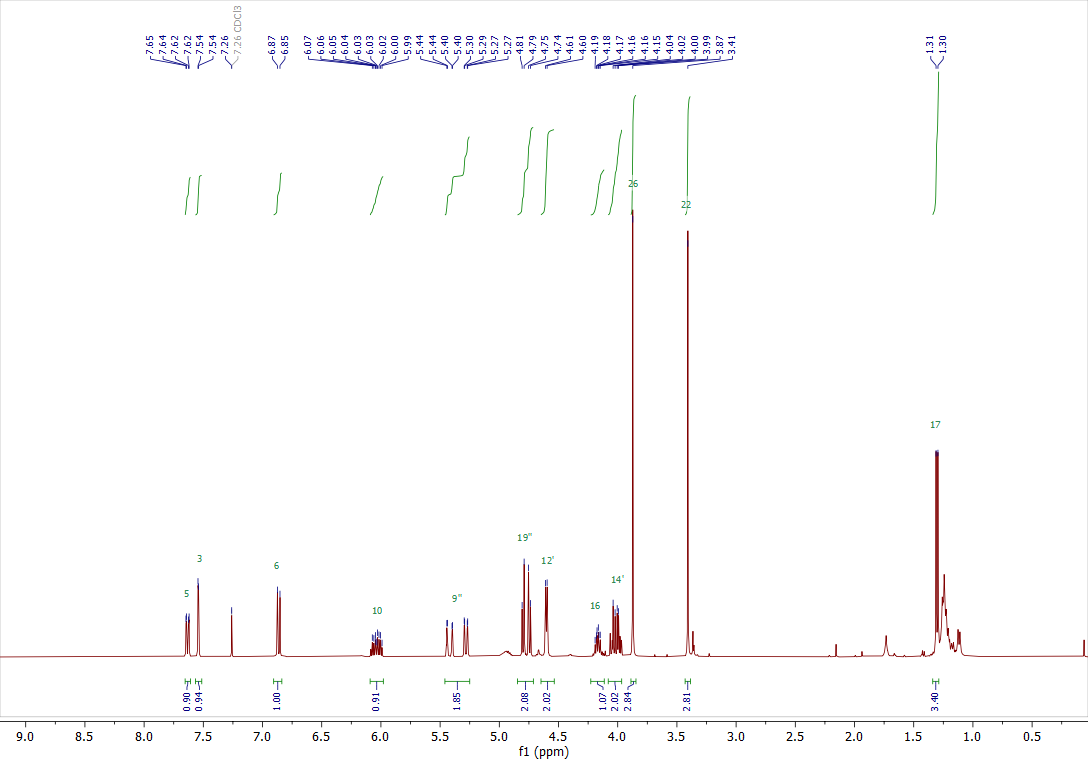
_

^1^H NMR Spectra of Compound **23** in CDCl_3_

_
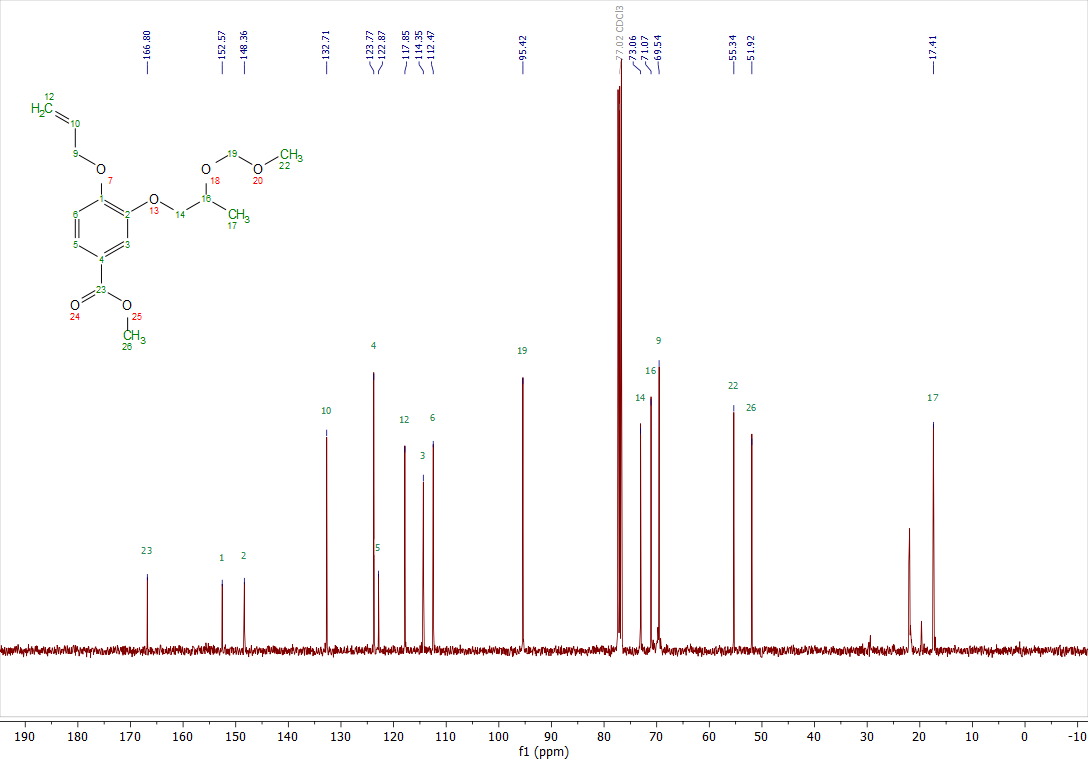
_

^13^C NMR Spectra of Compound **23** in CDCl_3_

_
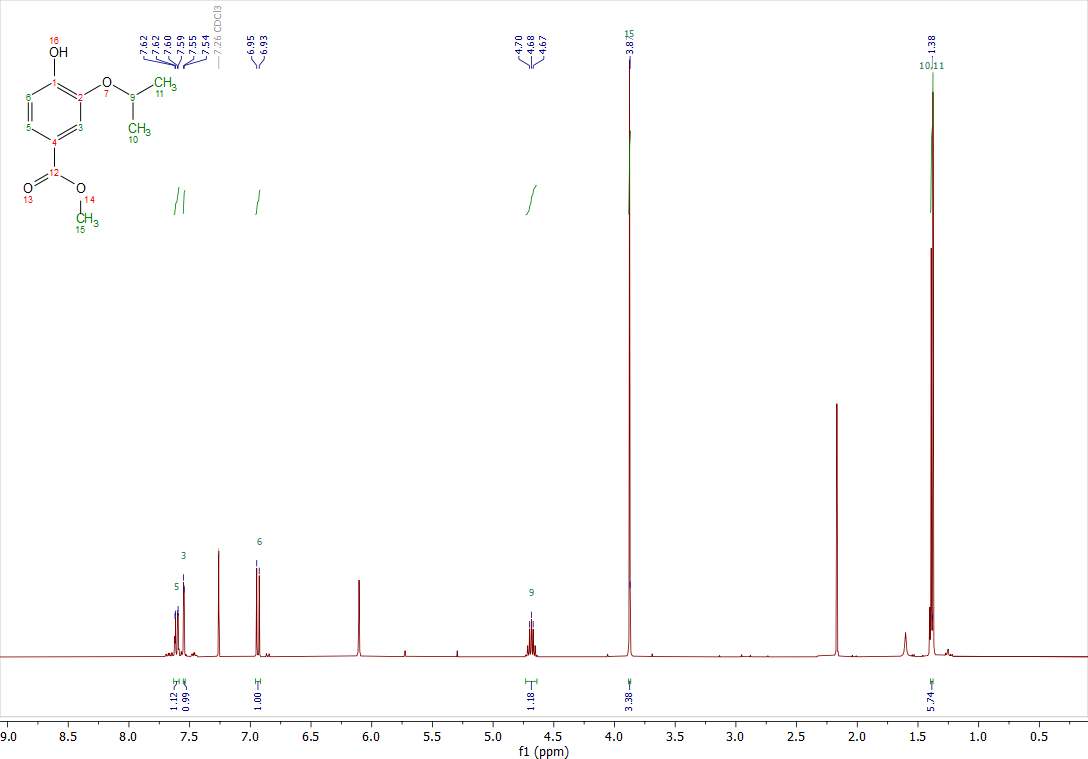
_

^1^H NMR Spectra of Compound **24** in CDCl_3_

_
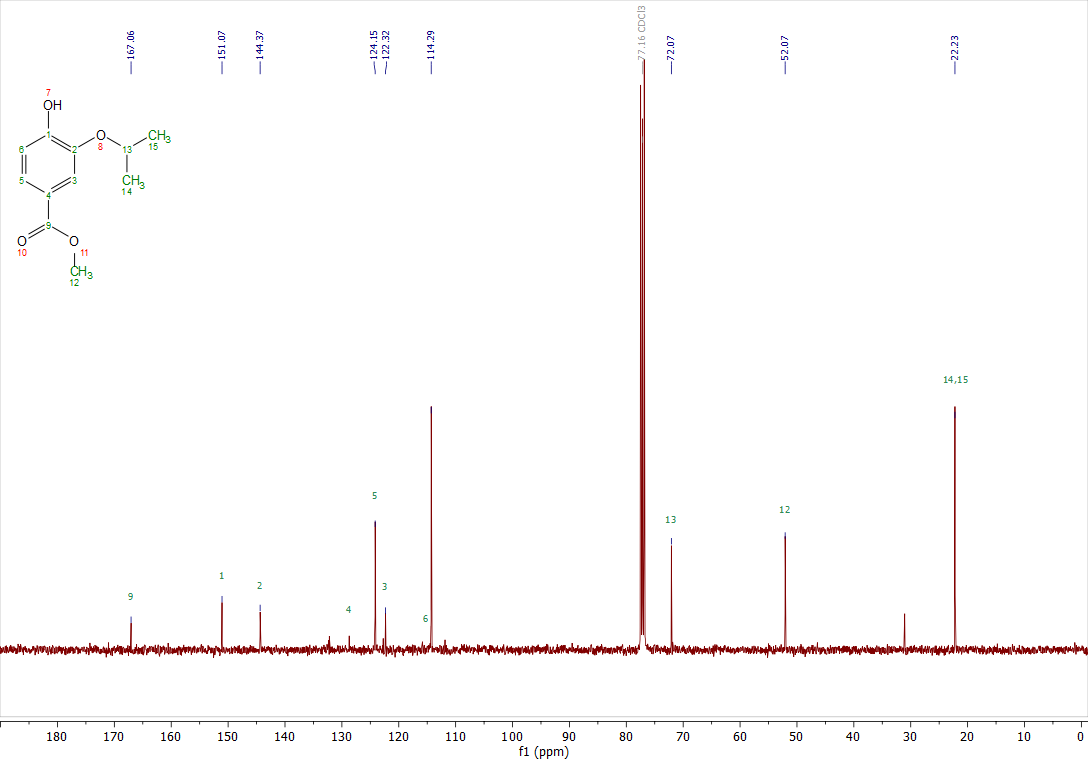
_

^13^C NMR Spectra of Compound **24** in CDCl_3_

_
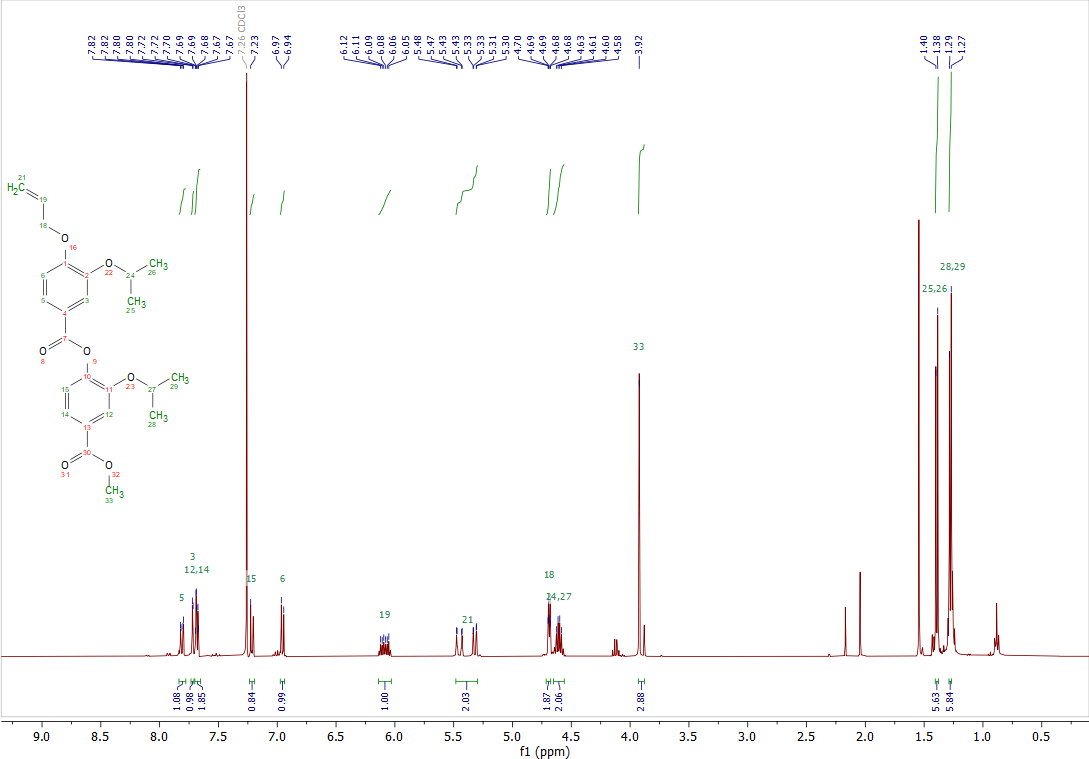
_

^1^H NMR Spectra of Compound **25** in CDCl_3_

_
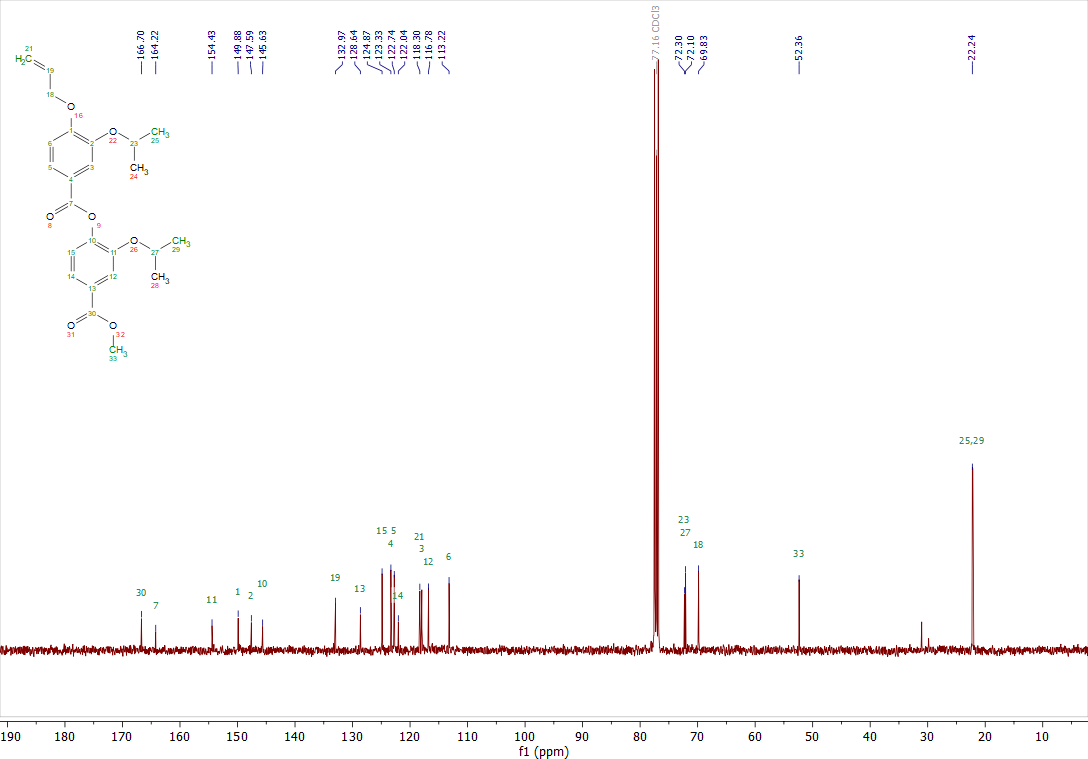
_

^13^C NMR Spectra of Compound **25** in CDCl_3_

_
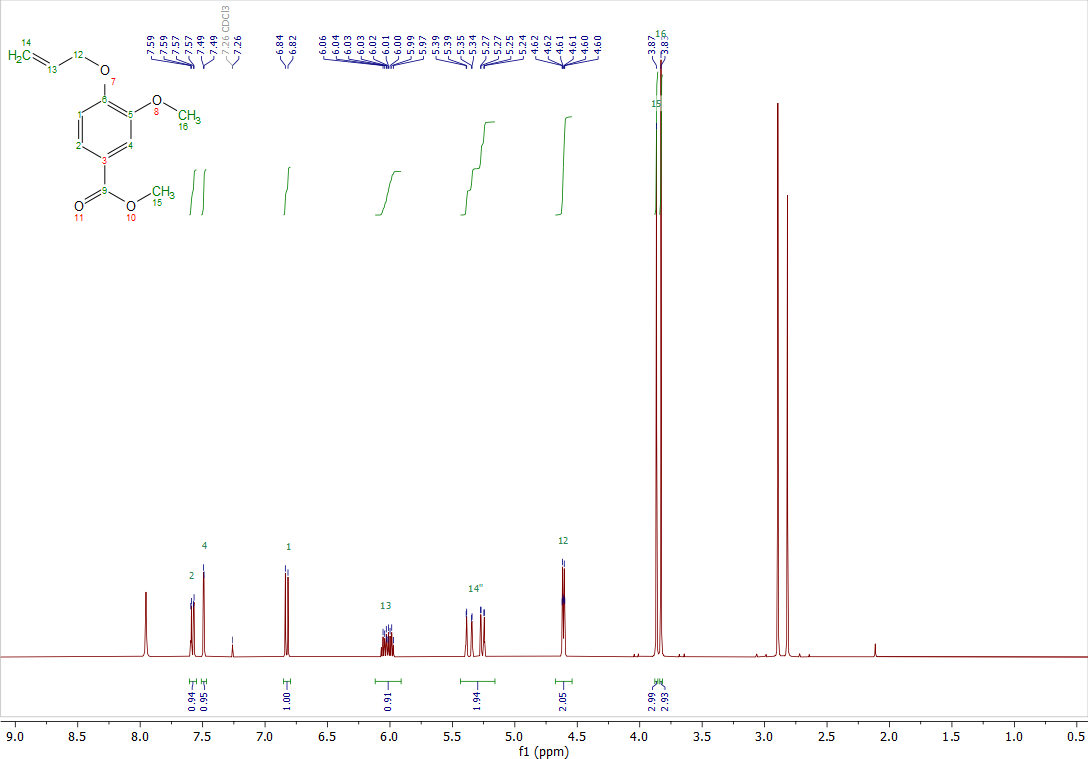
_

^1^H NMR Spectra of Compound **26** in CDCl_3_


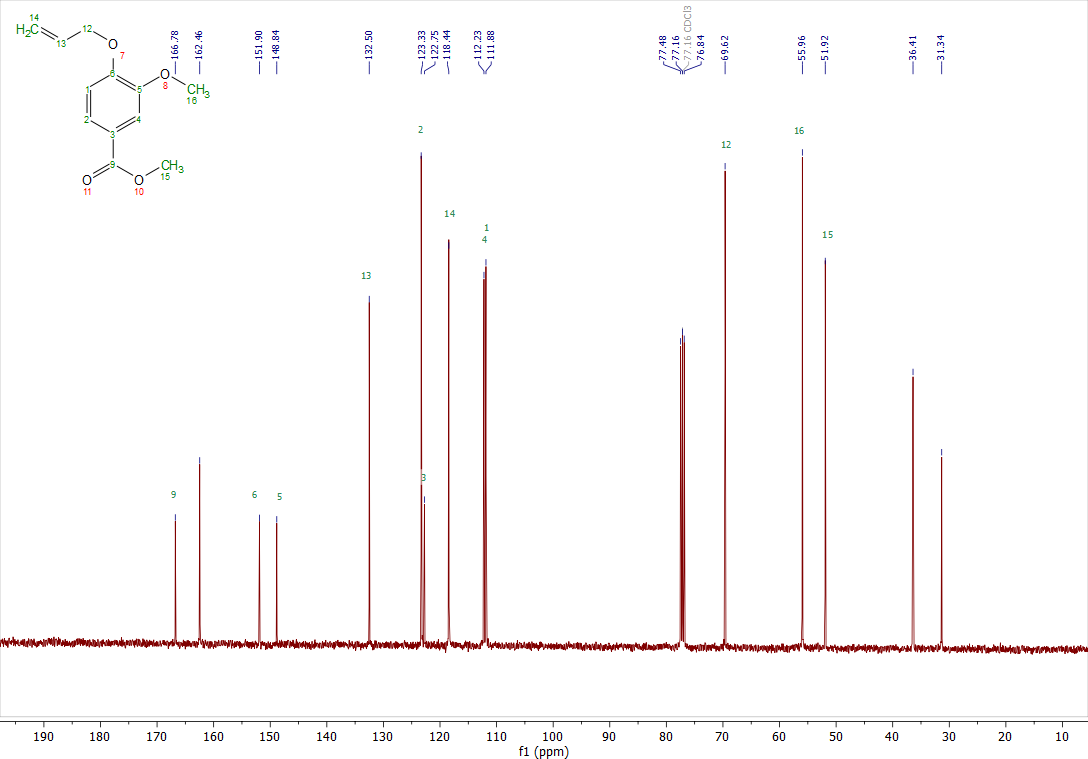


^13^C NMR Spectra of Compound **26** in CDCl_3_

_
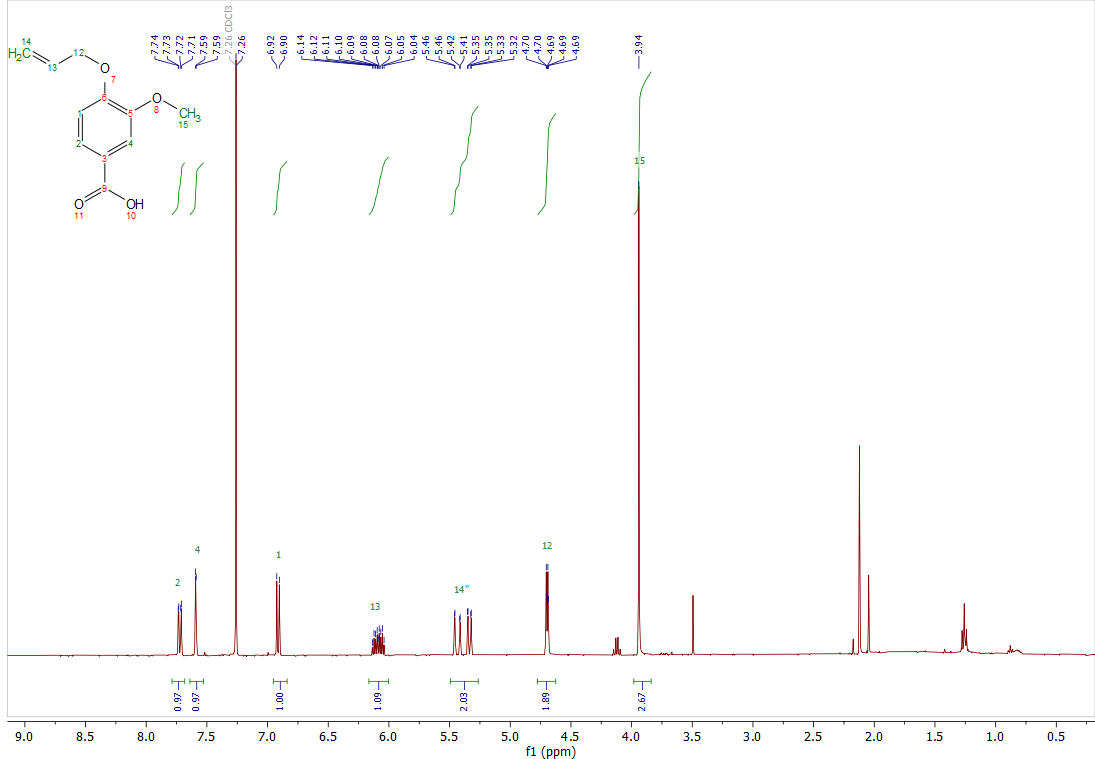
_

^1^H NMR Spectra of Compound **27** in CDCl_3_

_
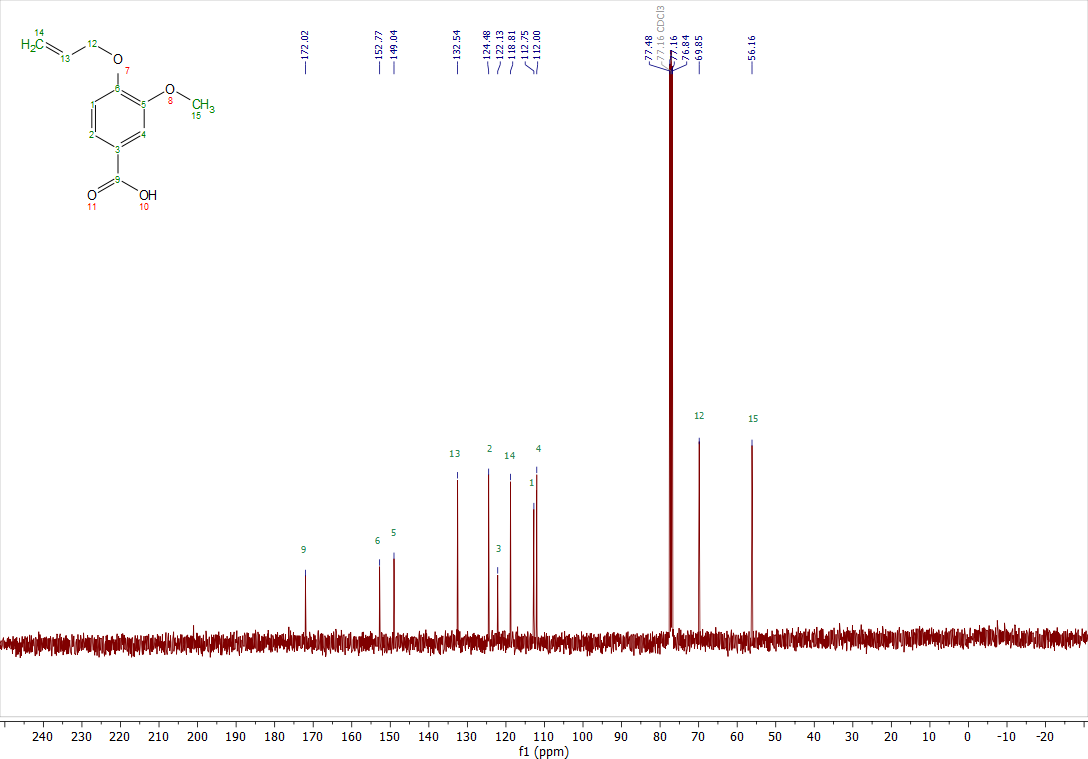
_

^13^C NMR Spectra of Compound **27** in CDCl_3_

_
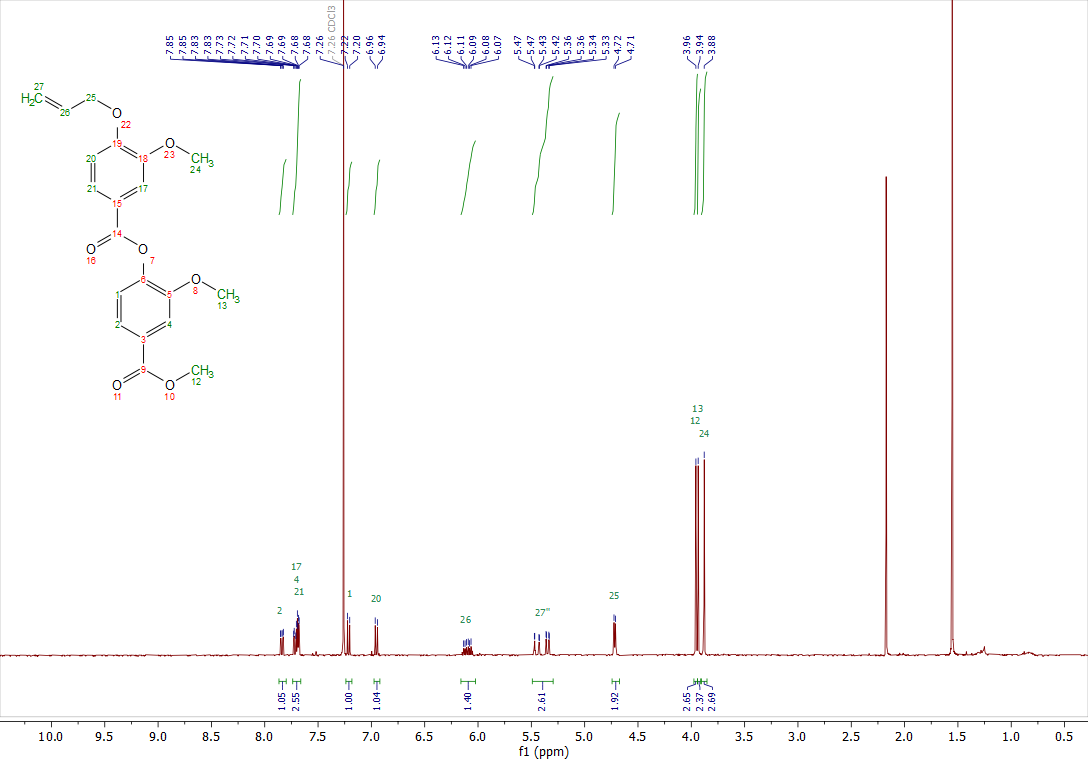
_

^1^H NMR Spectra of Compound **28** in CDCl_3_

_
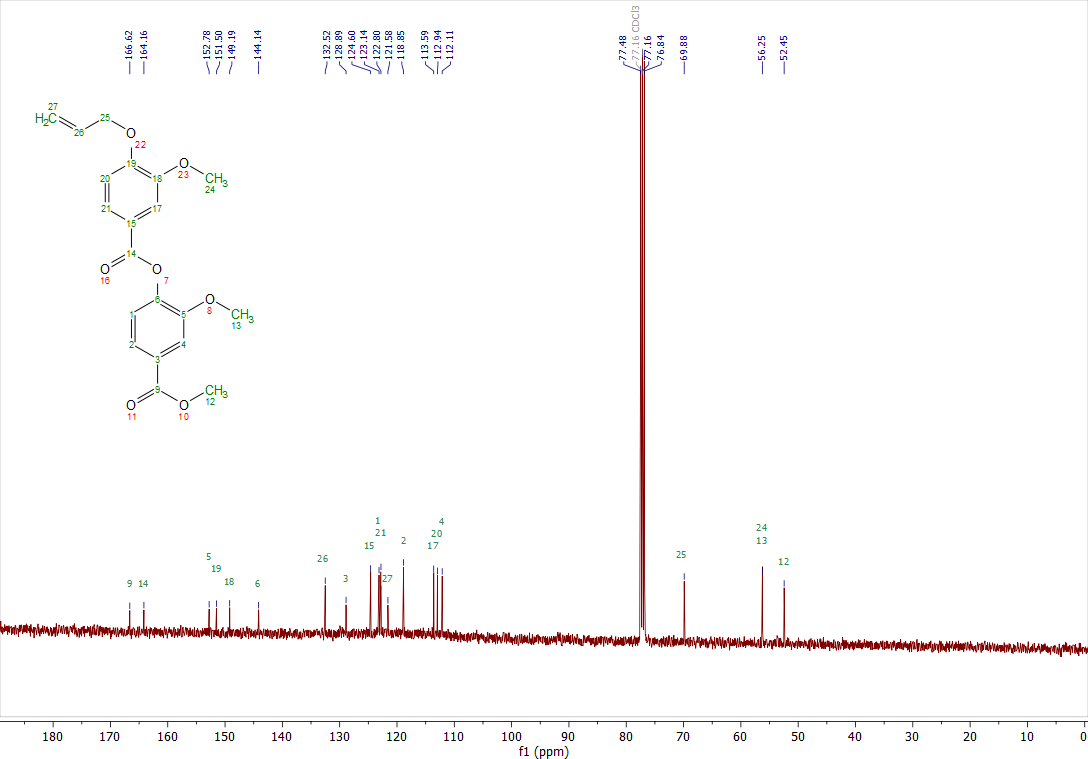
_

^13^C NMR Spectra of Compound **28** in CDCl_3_

_
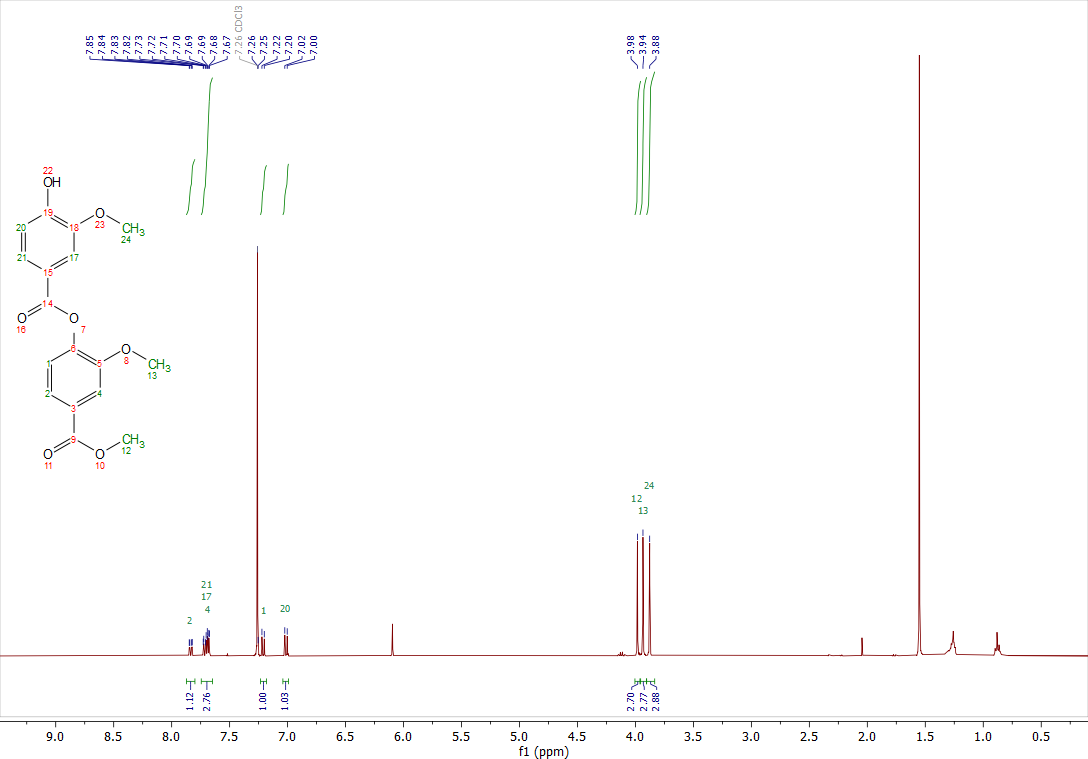
_

^1^H NMR Spectra of Compound **29** in CDCl_3_

_
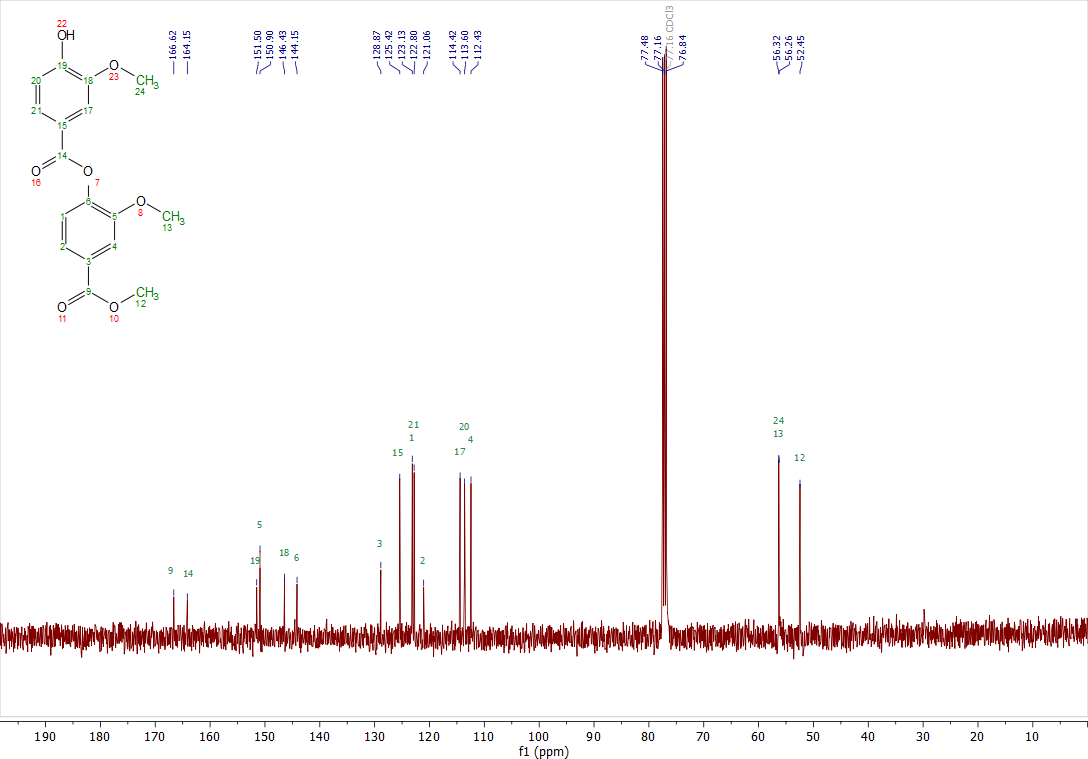
_

^13^C NMR Spectra of Compound **29** in CDCl_3_

**References**

[1] O. V. Dolomanov, L. J. Bourhis, R. J. Gildea, J. a. K. Howard, H. Puschmann, *J. Appl. Crystallogr.* **2009**, *42*, 339–341.

[2] G. M. Sheldrick, *Acta Crystallogr. Sect. C Struct. Chem.* **2015**, *71*, 3–8.
